# Polyacrylate Vitrimer Network via In Situ Isocyanide Copolymerization: Synthesis and Molecular Dynamics

**DOI:** 10.1021/jacs.5c22502

**Published:** 2026-03-06

**Authors:** Han-Li Sun, Stavros X. Drakopoulos, Lejla Čamdžić, Shawn M. Maguire, Rodney D. Priestley, Erin E. Stache

**Affiliations:** † Department of Chemistry, 6740 Princeton University, Princeton 08544, New Jersey, United States; ‡ Princeton Materials Institute, Princeton University, Princeton 08544, New Jersey, United States; § Department of Chemistry and Chemical Biology, Cornell University, Ithaca, New York 14853, United States; ∥ Department of Chemical and Biological Engineering, Princeton University, Princeton 08540, New Jersey, United States

## Abstract

Widespread plastic pollution highlights the urgent need for materials with sustainable end-of-life management. Vitrimers, cross-linked polymers containing dynamic covalent bonds, combine the durability of thermosets with their recyclability. Here, we report a one-step photocopolymerization using multifunctional isocyanides as readily accessible cross-linkers that directly introduce vinylogous urethane-like linkages into polyacrylate networks, structures difficult to obtain via amine–β-ketoester condensation. The resulting materials show good reprocessability, maintaining a comparable mechanical performance after three processing cycles. Broadband dielectric spectroscopy (BDS) reveals that the temperature dependence of the bond-exchange relaxation times evolves from a kink-like response in fresh samples to Arrhenius behavior after annealing, visualizing topological rearrangement and defect healing. A scaling relationship between bond-exchange relaxation and electrical conductivity establishes that the former is the underlying mechanism for charge transport in vitrimers. Furthermore, dipolar intermediates generated during bond exchange increase the dielectric permittivity, providing new insight into designing sustainable dielectric materials.

## Graphical abstract

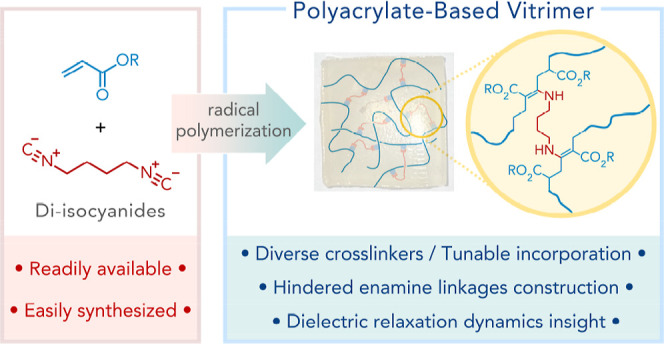

## Introduction

The demand for sustainable materials has increased due to growing concerns over the environmental impact of plastic waste accumulation. Thermosets comprise a significant proportion of all commodity polymers and are extensively used in construction, the automotive industry, and electronics. Their durable, cross-linked structures provide outstanding mechanical and thermal properties, as well as excellent chemical resistance. These benefits, however, prevent them from being easily reprocessed, thus limiting their recyclability.^1,2^ To address this challenge, the incorporation of dynamic covalent linkages has emerged as a useful strategy for constructing reprocessable polymer networks, effectively creating a more circular plastic economy. These dynamic linkages can undergo reversible exchange reactions in response to external stimuli such as heat or light, endowing the materials with thermoplastic-like behavior despite their covalent cross-linking.^3–16^ In this way, these materials can behave as both thermosets and thermoplastics in a controllable manner. This class of materials is known as covalent adaptable networks (CANs), with a subclass where bond exchange is activated at elevated temperatures, referred to as vitrimers.^17^ Vitrimers are currently being explored for a wide range of applications—not only in structural materials such as lightweight, high-strength components where recyclable thermosets are desirable, but also in specialty applications including biomedical devices, packaging materials, and soft robotics, where self-healing and shape-changing capabilities are required.^7,8,16^

To enable dynamic covalent cross-linking, vitrimers frequently employ carbonyl derivatives,^18–24^ owing to their wide availability and synthetic accessibility. Among these systems, vinylogous urethane linkages have emerged as a particularly well-studied motif due to their highly tunable exchange kinetics and robustness when reprocessed.^25^ The typical synthetic strategy involves the direct condensation of free amines with β-keto esters or amides. For instance, the Du Prez group demonstrated polycondensation between small-molecule amines and ester or amide counterparts.^26^ Alternatively, the Sumerlin group synthesized linear poly­(methyl methacrylate) (PMMA) containing β-keto ester side chains, followed by a postpolymerization cross-linking step.^27,28^ Among these strategies, the structure of the amine cross-linkers is highly tunable. However, the structural diversity of the carbonyl counterparts remains underexplored. In particular, sterically hindered carbonyl counterparts are rarely utilized primarily because the efficient condensation of such carbonyls with free amines remains challenging. This difficulty arises from the significant impact of steric hindrance on the thermodynamics of enamine formation.^29–31^ Consequently, despite the widespread availability of carbonyl compounds, this considerably restricts the design and diversification of new vitrimer networks (Figure 1a).

**Figure 1:**
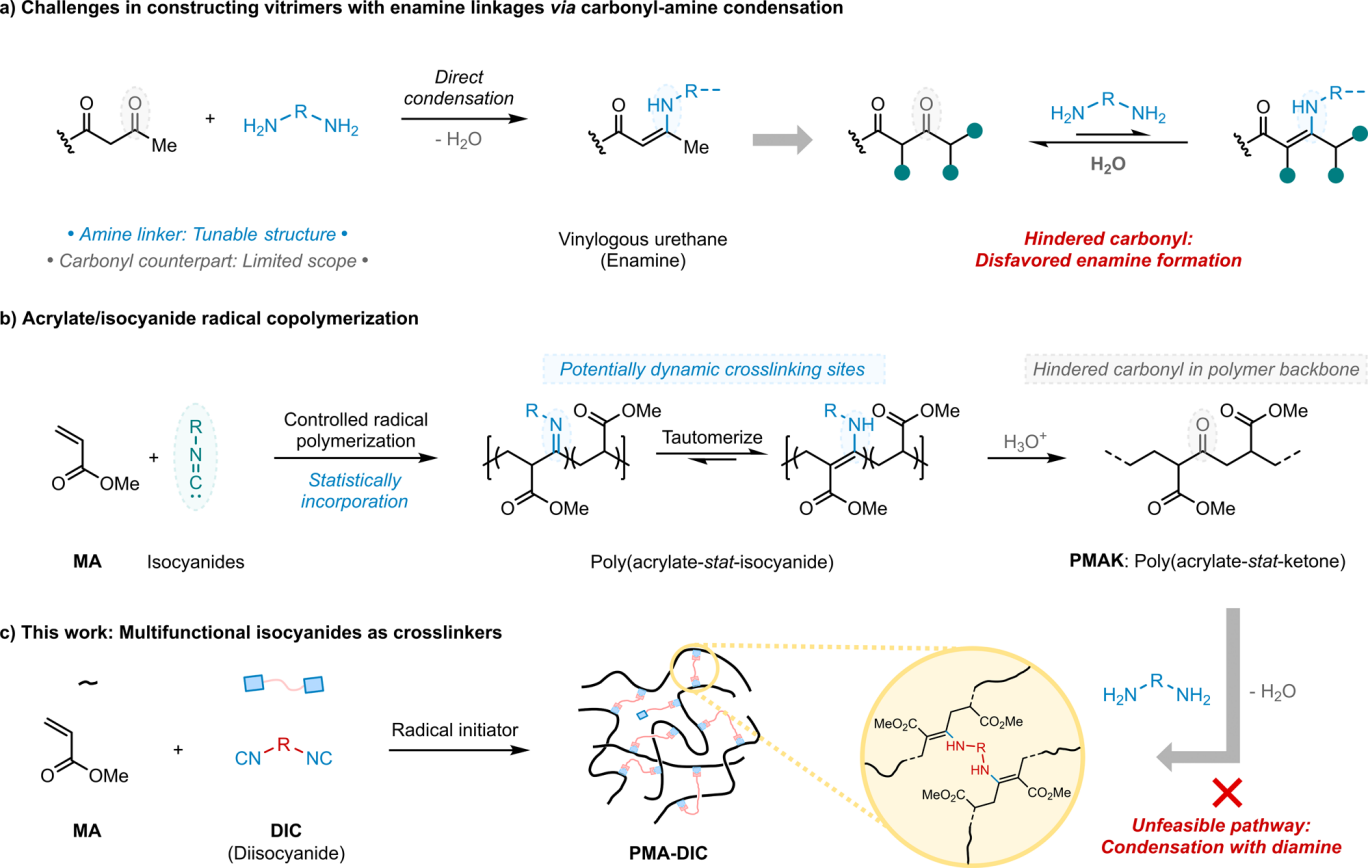
Radical copolymerization of acrylates and isocyanides. (a) Challenges of carbonyl/free amine condensation cross-linking strategy: thermodynamically unfavored enamine formation. (b) Acrylate and isocyanide copolymerization. (c) This work: directly incorporate hindered vinylogous urethane linkages into polyacrylates using multifunctional isocyanides as cross-linkers.

To expand the structure design space, we envisioned developing alternative monomers capable of forming highly dynamic enamine linkages within vitrimer networks. Recently, isocyanides have emerged as effective comonomers for directly introducing imine moieties into polymer backbones through radical copolymerization (Figure 1b).^32,33^ Notably, the resulting β-imino ester microstructures in the obtained poly­(acrylate-*co*-isocyanide) copolymer spontaneously tautomerize into vinylogous urethane-like enamine structures, which inherently feature sterically hindered configurations.

Furthermore, in the presence of a free amine, we discovered that amine exchange can still proceed within these copolymers despite substantial steric hindrance (Figure S11). Based on these observations, we propose that such readily synthesizable multifunctional isocyanides represent a promising class of enamine linkage precursors for vitrimer fabrication. Importantly, this approach facilitates the one-step formation of dynamic covalent networks and broadens the accessible microstructural design space to encompass hindered enamines, thereby providing valuable new insights into the design of this class of materials. Furthermore, we employed broadband dielectric spectroscopy (BDS) and dynamic mechanical analysis (DMA) to investigate how these hindered structures respond to bond exchange relaxation and the associated dynamic bond exchange behavior.

## Results and Discussion

We commenced our investigation by studying the solvent-free polymerization reaction of methyl acrylate (MA) and 1,4-diisocyanobutane (DIC^Bu^) under free-radical conditions. We selected phosphine oxide-based initiators, with the commercially available phenylbis­(2,4,6-trimethylbenzoyl)­phosphine oxide (BAPO) was chosen for its strong absorption above 400 nm, allowing efficient initiation under white LED irradiation. Upon photocuring MA, using 2 mol % DIC^Bu^ and 0.3 mol % BAPO, we obtained a transparent, stretchy sample with a high gel fraction exceeding 90% in tetrahydrofuran (THF), indicating a high degree of cross-linking (Table 1, entry 1). Infrared (IR) shows increasing enamine peaks with an increasing loading of DIC^Bu^, indicating higher incorporation of isocyanides (Figure 2c).

**Table 1:**
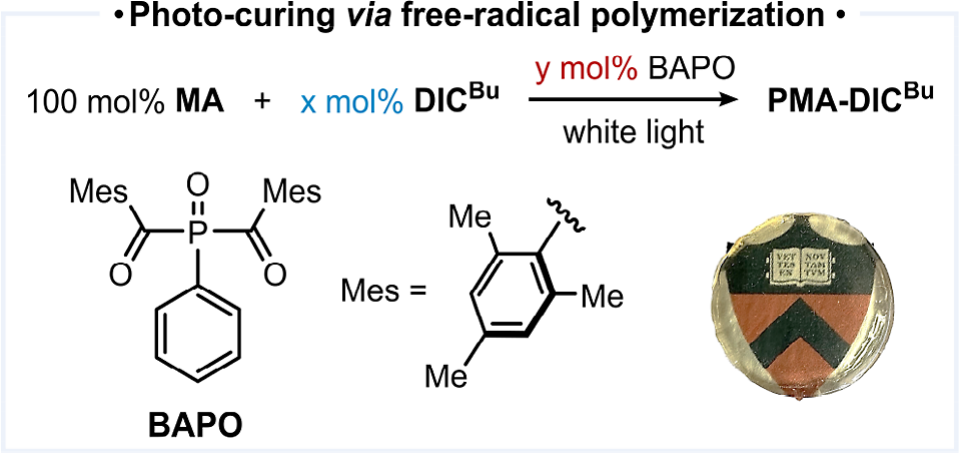
Evaluation of Free-Radical Copolymerization of MA and DIC^Bu^ ^a^

Entry	*x*	*y*	Mn (kDa)^b^	D̵^b^	Gel fraction (%)^c^
1	2	0.3	7.9	2.3	90
2	3	0.3	7.4	2.2	90
3	5	0.3	4.3	2.0	99
4	10	0.3	2.8	2.3	90
5	15	0.3	2.0	1.7	89
6	5	0.1	8.7	1.8	96
7	5	0.6	2.4	1.4	94

a Reactions were carried out using 4.0 mmol MA under N_2_ atmosphere. The photo demonstrates the film’s transparency, revealing the Princeton logo underneath.

b Number-average molecular weight (*M*_n_) and dispersity (*D̵*) were determined with samples after hydrolysis by GPC relative to polystyrene standards.

c Gel fraction in THF. (© The Trustees of Princeton University. Logo reproduced with permission).

**Figure 2:**
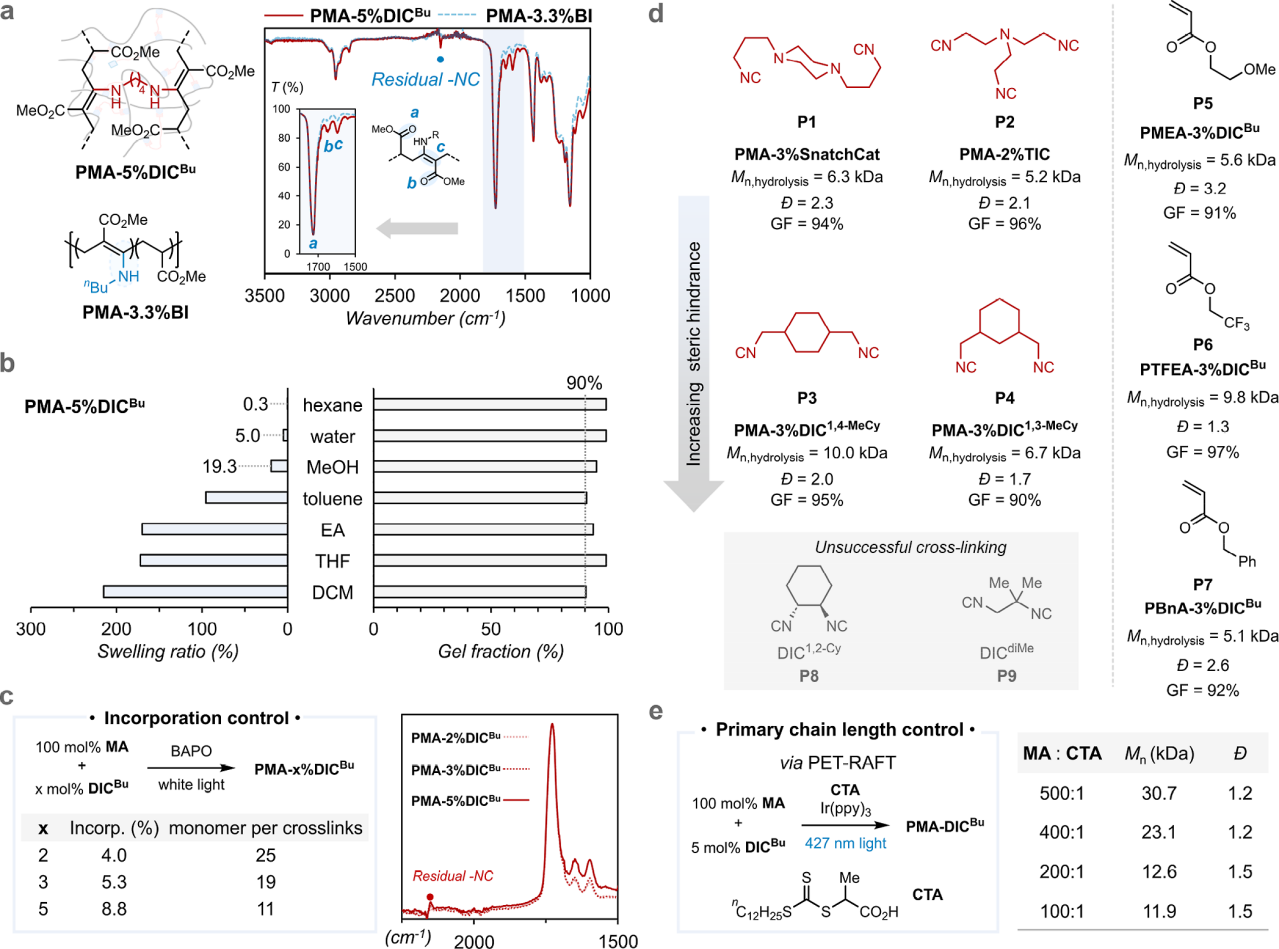
Structural analysis and reaction developments of the PMA-isocyanide network. (a) Overlaid IR spectra of **PMA-5%DIC** ^**Bu**^ (red) and **PMA-3.3%BI** (blue) (intensities were normalized against the highest carbonyl peak). Percentages represent the initial loading of multi-isocyanide for cross-linked samples. For linear polymers, this indicates precise incorporation, as determined by NMR. (b) Swelling ratio and gel fraction of **PMA-5%DIC** ^**Bu**^ in different solvents. (c) Incorporation control with different DIC^Bu^ loading (IR absorption spectra are shown on the right; intensities were normalized against the highest carbonyl peak). (d) Isocyanide and monomer scope. (condition: 4.0 mmol monomer, [monomer]/[-NC]/[BAPO] = 100/6/0.3, under white light, room temperature under N_2_) (e) Demonstration of control over primary chain length via the PET-RAFT mechanism. (condition: 4.0 mmol MA, 5 mol % DIC^Bu^, 0.1 mol % Ir­(ppy)_3_, room temperature under N_2_).

The cross-linked samples were hydrolyzed to determine the primary chain length. The molecular weights (*M*_n_) (demonstrated by the *M*_n_ of hydrolyzed samples) of each sample displayed a significant decrease with an increasing DIC^Bu^ loading (Table 1, entries 2-3). We speculate that this trend may arise from more rapid chain termination and chain transfer events, stemming from the formation of a reactive imidoyl radical during isocyanide propagation. Control experiments with increasing monomer ratios of monoisocyanide were conducted to support our hypothesis, and a similar decrease in chain length was observed (Table S2). Interestingly, further increasing the DIC^Bu^ loading resulted in a lower cross-linking density, as indicated by a reduction in the gel fraction (Table 1, entries 4-5). This reduction can be attributed to the excessive shortening of the primary chain length, which overwhelms the increase of isocyanide incorporation, resulting in fewer cross-linking sites per chain. We conducted additional studies on various ratios of BAPO to the monomers (Table 1, entries 6-7) and observed a significant impact of BAPO loading on primary chain length. As expected, higher loading resulted in shorter primary chains due to the initiation of more chains.

Although multiple linear MA-isocyanide copolymers were synthesized and systematically characterized in our previous studies,^32,33^ we conducted additional characterizations to confirm the primary structure of the cross-linked samples. Due to its low solubility, no NMR signals could be observed in common deuterated solvents. To address this issue, a linear copolymer of MA and *n*-butyl isocyanide (**PMA-3.3%BI**) was synthesized independently via controlled radical polymerization, following our previously reported procedure, serving as an IR ref 33 IR analysis revealed that the sample obtained from 5 mol % DIC^Bu^ (Table 1, entry 3, named **PMA-5%DIC** ^**Bu**^) and **PMA-3.3%BI** exhibited a remarkable matching in all peaks, supporting the presence of the same chemical structures (Figure 2a). No characteristic peak of MA was detected, confirming its complete conversion. Only trace amounts of residual –N≡C groups were observed at 2148 cm^–1^, confirming the high conversion of DIC^Bu^. Like linear **PMA-3.3%BI**, two peaks at 1650 cm^–1^ and 1595 cm^–1^ were observed, corresponding to C=O (ester) and C=C bonds in vinylogous urethanes, respectively. The wavenumbers agree with those reported for similar structures in the literature.^26,34^ The degree of cross-linking of **PMA-5%DIC** ^**Bu**^ was further evaluated by investigating the swelling ratio and gel fraction in different solvents (Figure 2b). Most solvents exhibit relatively low swelling ratios (all less than 180%), except for dichloromethane (DCM), which reaches 215%, likely due to the increased solubility of the PMA segments. Additionally, the gel fraction in all solvents exceeds 90%. Collectively, these results indicate a high degree of cross-linking.

As shown in Table 1, the cross-linking density of the network is adjustable with varying cross-linker loading (Figure 2c). Isocyanide incorporation was calculated from IR spectra with the relative intensity between the C=O bond (peak a) and the C=C bond (peak c), using **PMA-3.3%BI** as a standard (Figures S34–3S6). An incorporation ratio of 4.0% was calculated based on **PMA-2%DIC** ^**Bu**^, which coincided well with the disappearance of the –N≡C peak, confirming the accuracy of the estimation. For samples with higher DIC^Bu^ loading, the incorporation increases accordingly; however, this is accompanied by an increased amount of residual isocyanide. Despite this, the cross-linking degree remains tunable by adjusting the feed ratio.

We evaluated various isocyanides and monomers to demonstrate the versatility of cross-linkers and the primary chemical structure in this material, which is crucial for further functional design. Different isocyanide-containing cyclic structures or heteroatoms were synthesized and tested (Figure 2d). Tertiary amine and piperazine were well tolerated (**P1-2**), with the corresponding copolymers exhibiting high gel fractions (over 90%). Di-isocyanides with rigid cyclohexane linkers were also successfully cross-linked (**P3-4**); however, a more significant –N≡C peak can be observed in the corresponding IR spectra, meaning lower conversion (Figures S16 and S18). This suggests that the incorporation efficiency of isocyanides is likely sensitive to steric effects. To verify this hypothesis, we tested DIC^1,2‑Cy^, which is directly connected to secondary isocyanides (**P8**). The gel fraction was significantly reduced, indicating unsuccessful cross-linking. With this result in hand, we proposed that by controlling steric hindrance, it might be possible to distinguish the reactivity of the two different isocyanide groups in one cross-linker, thereby enabling the formation of non-cross-linked polymers. To test this, we synthesized DIC^diMe^, which contains one hindered tertiary isocyanide and one sterically accessible isocyanide (**P9**). Due to the significant steric hindrance, no crosslinking was achieved as expected. However, NMR analysis indicated 0% isocyanide incorporation. This can be attributed to steric congestion at the β-position, which destabilizes the resulting enamine and leads to its rapid hydrolysis to a ketone moiety. Additionally, various acrylates were tested (**P5-7**). Both the hydrophilic 2-methoxyethyl acrylate (MEA) and the hydrophobic 2,2,2-trifluoroethyl acrylate (TFEA) exhibited successful cross-linking. Notably, DIC^Bu^ was readily incorporated into the more electron-deficient TFEA, where no isocyanide residue was detected in the IR spectrum. This observation is consistent with previous studies.^32^

Primary chain length has been reported as an essential factor influencing the properties of vitrimers.^35–37^ Since imidoyl radicals can undergo fast chain termination and chain transfer, reducing the primary chain length under free radical curing conditions, we considered introducing dormant radical species during polymerization to suppress these undesired processes by lowering the concentration of active radical chain ends. Typical photoinduced electron transfer reversible addition-fragmentation chain transfer (PET-RAFT) polymerization conditions were used for curing at 5 mol % DIC^Bu^ loading (Figure 2e). As expected, these samples yielded significantly higher molecular weights after hydrolysis compared to free radical-cured samples. Notably, we can control the chain length by adjusting the loading of the chain transfer agent (CTA). However, longer curing times are required for PET-RAFT conditions, which is attributed to the reduction of the radical chain-end concentration.

After comprehensively characterizing the chemical structure of the vitrimers, we evaluated their reprocessability. It has been established that enamines undergo associative bond exchange.^38,39^ Therefore, external free amines are generally incorporated into the network as catalysts to facilitate bond exchange. Based on these findings, we synthesized **PMA-3%DIC** ^**Bu**^ **-0.15%BD** under the same radical copolymerization conditions by incorporating 0.15 mol % of 1,4-butyl diamine (BD) during the curing process as a bond-exchange activator.

The reprocessability of the samples was preliminarily assessed after hot-pressing at 2 MPa and 60 °C. **PMA-3%DIC** ^**Bu**^ **-0.15%BD** gives a uniform thin film, demonstrating improved reprocessability and confirming the role of BD as an activator (Figure 3a). Notably, the incorporation of BD did not alter the chemical structure, as evidenced by the identical IR spectra to those of the sample synthesized without BD (**PMA-3%DIC** ^**Bu**^). Additionally, incorporation of BD does not significantly affect the primary chain length or the gel fraction of the samples. DMA was conducted for the **PMA-3%DIC** ^**Bu**^ **-0.15%BD** sample (Figure 3b). The storage modulus (*E*′) reaches a rubbery plateau at approximately 50 °C. A peak at 19 °C is observed in the tanδ_DMA_ curve, corresponding to the segmental relaxation that correlates with the glass transition temperature, which also aligns well with the DSC results (Figure 3c). **PMA-3%DIC** ^**Bu**^ **-0.15%BD** was shredded and successfully reprocessed via compression molding at 80 °C, 5.5 MPa (Figure 3d). The IR spectra obtained after reprocessing remained unchanged compared to the original **PMA-3%DIC** ^**Bu**^ **-0.15%BD** sample, indicating excellent retention of the chemical structure under the applied hot-pressing conditions (Figure 3e). The sample was subjected to three repeated shredding-compression cycles, and the mechanical properties of each cycle were evaluated by tensile testing. Upon reprocessing, the resulting materials exhibit tensile properties comparable to those of the original sample (Figure 3f).

**Figure 3:**
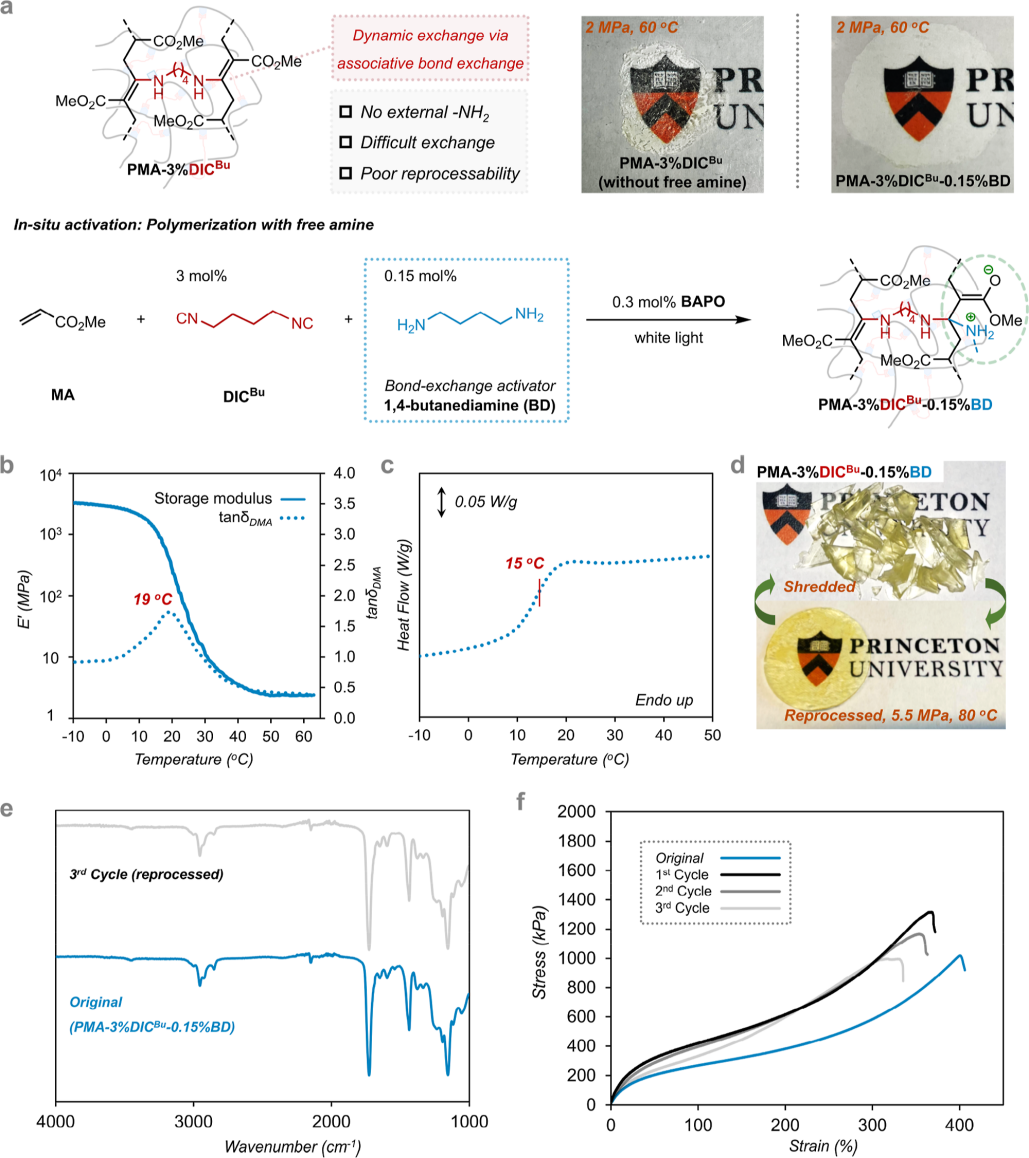
Mechanical properties, viscoelasticity, and reprocessing evaluation of **PMA-3%DIC** ^**Bu**^ **-0.15%BD**. (a) Preparation of the sample with free amine activator (**PMA-3%DIC** ^**Bu**^ **-0.15%BD**) and preliminary reprocessing attempts into a thin film (© The Trustees of Princeton University. Logo reproduced with permission). (b) Storage modulus and tanδ_DMA_ from DMA of **PMA-3%DIC** ^**Bu**^ **-0.15%BD**. (c) DSC curves of **PMA-3%DIC** ^**Bu**^ **-0.15%BD**. (d) Photos of shredded and reprocessed samples (© The Trustees of Princeton University. Logo reproduced with permission). (e) IR spectra of original and reprocessed **PMA-3%DIC** ^**Bu**^ **-0.15%BD** samples. (f) Representative stress–strain curves.

To understand bond-exchange dynamics and more comprehensively evaluate our vitrimer properties as a new material, we also performed BDS, a technique that studies the interaction of electromagnetic waves with matter over broad frequency and temperature ranges, providing information on dipolar fluctuations and other polarization and/or charge transport contributions.^40–42^ Over the past few years, research efforts have focused on deconvoluting the complicated relaxation dynamics of vitrimers, as observed through BDS, in a range of polymers and types of dynamic bonds,^43–49^ indicating a growing interest in this field.

Additionally, we observed a notable change in mechanical properties after annealing during sample preparation, so we decided to run dielectric measurements with fresh-made samples from low to high temperatures to monitor changes in the pristine network. Figure 4 focuses on the dielectric relaxation behavior of the developed vitrimers and their comparison to nondynamically cross-linked PMA, specifically **PMA-2%diacrylate**. In Figure 4a, the experimentally observed real part of dielectric permittivity as a function of temperature is shown for the vitrimer sample **PMA-2%DIC** ^**Bu**^ **-0.10%BD** at 0.1 Hz, where two steps can be observed at approximately 10 and 35 °C, corresponding to the segmental and bond exchange relaxations, respectively. The segmental relaxation (commonly called α-relaxation) originates from the glass transition process, as discussed earlier in Figure 3b for the DMA data. The bond exchange relaxation arises from the charge separation intermediate during bond exchange that generates a dipole, as highlighted in Figure 3a. Since the bond exchange relaxation is clearly visible in the real part of dielectric permittivity, we subsequently employed the derivative permittivity, ε″_der_, method to visualize the various polarization phenomena in a 3D representation as a function of both the temperature and frequency for **PMA-2%DIC** ^**Bu**^ **-0.10%BD** (Figure 4b). The derivative permittivity formalism is shown in Figure 4b and eq S3, and it allows us to remove additional contributions in ε″ (like the electrical, direct current, dc conductivity) from the dielectric spectrum, helping us to visualize the bond exchange relaxation directly.^51,52^

**Figure 4:**
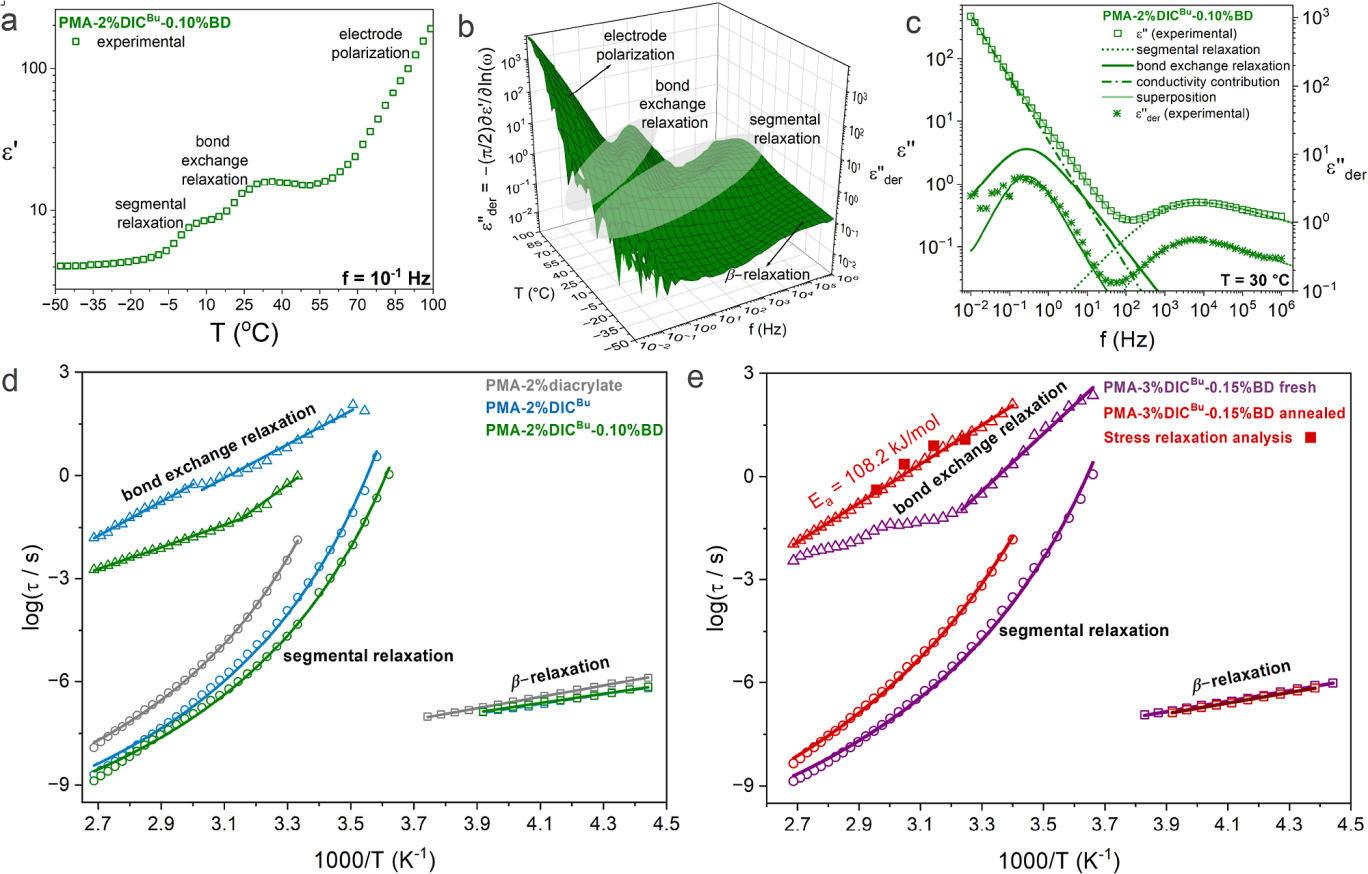
Dielectric response and relaxation dynamics of the samples under study. The dielectric analysis was completed based on the complex dielectric permittivity function, ε*(ω) = ε*′*(ω) – *i*ε″(ω)*,* where ω is the angular frequency equal to *2*π*f* with *f* being the frequency of the externally applied electric field. As ε*′* and ε″ are the real and imaginary parts of dielectric permittivity. (a) Real part of dielectric permittivity as a function of temperature at 10^–1^ Hz of **PMA-2%DIC** ^**Bu**^ **-0.10%BD** where 3 contributions to the dielectric spectrum are identified, namely segmental relaxation, bond exchange relaxation, and electrode polarization. (b) 3D representation of the ε″_der_ against frequency and temperature with the corresponding dielectric phenomena highlighted for clarity for **PMA-2%DIC** ^**Bu**^ **-0.10%BD**. (c) Imaginary part of dielectric permittivity and the ε″_der_, both as a function of frequency at 30 °C of **PMA-2%DIC** ^**Bu**^ **-0.10%BD** where the open symbols correspond to the experimental data and the lines are Havriliak–Negami (HN) fittings. The experimentally obtained isothermal dielectric spectra were fitted with the HN function in its generalized complex permittivity form^50^ as shown in eq S1, with the fitting shape parameters *m* and *n* (Table S5). The ε″_der_ values are also fitted with the HN function after derivation. (d) Relaxation map of samples with 2% cross-linker and (e) relaxation map comparing **PMA-3%DIC** ^**Bu**^ **-0.15%BD** before and after annealing. With solid squares, the stress relaxation analysis obtained by rheology (Figures S48 and S49) yielded comparable relaxation times and *E*_a_ with BDS for bond exchange relaxation.

To obtain the temperature dependence of the bond exchange relaxation, we analyzed the dielectric data via the imaginary part of the dielectric permittivity against frequency for **PMA-2%DIC** ^**Bu**^ **-0.10%BD** at 30 °C (Figure 4c). At low frequencies, we observe a straight line that ascends as the frequency decreases and is ascribed to the contribution of dc conductivity (also shown in eq S1 as a power law), especially at temperatures higher than the *T*_g_. Therefore, the dc conductivity shadows the contribution from bond exchange relaxation in the frequency domain; thus, we need to apply Havriliak–Negami fittings to deconvolute the various polarization contributions. As explained earlier (Figure 4b) the ε″_der_ mathematical treatment of the dielectric data removes the contribution of the dc conductivity; thus, it is easier to discern the bond exchange relaxation. Toward that purpose, we include the ε″_der_ data, as well as the HN fits, within the same graph for comparison reasons (Figure 4e). The ε″_der_ representation shows clearly the bond exchange relaxation peaks at approximately 0.2 Hz. At this temperature, the bond exchange relaxation appears to be very slow, indicating that around this temperature range lies the onset temperature for the bond exchange reaction at which the bond exchange is at an observable time scale and is associated with the topology freezing transition temperature (*T*_v_).^53,54^ *T*_v_ refers to the temperature where topological rearrangement is effectively frozen on an experimental time scale.^17^ Therefore, as the temperature drops, the relaxation time of the bond exchange relaxation increases significantly, indicating that the dynamic bonds form very slowly, and eventually the vitrimer behaves like a nondynamically cross-linked system.^55,56^

To understand the bond exchange relaxation behavior, the relaxation times obtained from the dielectric analysis are presented against the reciprocal temperature in the form of relaxation maps (Figure 4d,e). In Figure 4d, samples **PMA-2%DIC** ^**Bu**^ and **PMA-2%DIC** ^**Bu**^ **-0.10%BD** are compared against the nondynamically cross-linked **PMA-2%diacrylate** (synthesis described in Figures S40 and S41), which does not exhibit a bond exchange relaxation, as expected. A significant difference in relaxation times between the bond exchange relaxations of samples **PMA-2%DIC** ^**Bu**^ and **PMA-2%DIC** ^**Bu**^ **-0.10%BD** is visible, where the addition of BD appears to facilitate transamination, yielding faster relaxation dynamics. Despite the absence of BD, **PMA-2%DIC** ^**Bu**^ exhibits a bond exchange relaxation, which could be attributed to trace water that triggers a small portion of enamine hydrolysis and releases a trace amount of free amine (Figure S43).^57^ Therefore, the temperature dependence of the bond exchange relaxation times exhibits a slope change between lower and higher temperatures, which, in fresh samples, we hypothesize is related to unincorporated BD. The bond exchange relaxation in the **PMA-2%DIC** ^**Bu**^ **-0.10%BD** exhibits a reduced slope at higher temperatures, indicating a lower *E*_a_ for bond exchange. Above the breaking point, i.e., crossover temperature, the bond exchange rate increases significantly, allowing the free BD to be gradually incorporated into the vitrimer network (Table S7).

Segmental relaxation appears in intermediate temperatures and follows the Vogel–Fulcher–Tammann (VFT) eq (eq S5). The VFT fitting parameter *D* is a measure of fragility, i.e., how abruptly a glass-forming material solidifies upon supercooling.^58^ Compared to the nondynamically cross-linked PMA system (**PMA-2%diacrylate**), the vitrimer equivalent (**PMA-2%DIC** ^**Bu**^ **-0.10%BD**) shows significantly faster segmental relaxation dynamics that can be quantified by a decrease in Vogel temperature (*T*_0_) (Figure 4d). This deviation was recently studied by the Schweizer group,^59^ and was ascribed to a reduction in the jump distance of the Kuhn segments in the presence of dynamic bonds as the degree of supercooling increases. Moreover, the observed decrease in *T*_0_ becomes stronger with increased loading due to the unincorporated DIC^Bu,^ which acts as a plasticizer as the loading increases.^60^ This hypothesis is in accordance with the IR results presented earlier (Figure 2c) that indicate the increase of the unincorporated –NC group with a higher DIC^Bu^ feed ratio. DSC and DMA experimental results shown in Figures S45 and S46 support the presented dielectric analysis, where a reduction in the *T*_g_ is observed with increasing DIC^Bu^. Moreover, the increase of DIC^Bu^ is accompanied by a decrease in primary chain length, which is significantly lower than the entanglement molecular weight (*M*_e_) of PMA,^61^ which is also expected to lower the *T*_g_.

To further investigate the topological rearrangement, we switched to a sample with higher cross-linking density (**PMA-3%DIC** ^**Bu**^ **-0.15%BD**) and compared the relaxation dynamics between a fresh sample and one that underwent thermal treatment (annealing) at 50 °C (above *T*_g_ and crossover temperature) overnight (Figure 4e). The bond exchange relaxation after annealing follows Arrhenius behavior, indicative of a single activation energy (*E*_a_ = 108.2 kJ/mol) over the tested temperature range. This value is higher than those reported for classic vinylogous urethanes in the literature,^26,27^ which can be attributed to the increased steric hindrance introduced by the molecular structure. On the other hand, the bond exchange relaxation exhibited by the fresh **PMA-3%DIC** ^**Bu**^ **-0.15%BD** sample shows a plateau, i.e., relaxation time freezing, ascribed to the process of topological rearrangement, which is facilitated at temperatures above the crossover temperature (breaking point). This topological rearrangement refers to topological defects gradually resolved by bond exchange, thus increasing the cross-linking density and hindering bond exchange relaxation. At temperatures above the plateau, the relaxation time of the fresh sample converges with that of the annealed sample, which implies that within the examined temperature range, the fresh sample has not yet reached full topological rearrangement. Additional evidence can be seen in the dielectric strength, Δε, values that decrease significantly with temperature as a result of the gradual BD incorporation (Figure S57). This observation implies the transformation of free BD to enamine (incorporation), considering that free BD possesses greater polarizability due to its higher mobility as a result of more degrees of freedom. Considering that sample **PMA-2%DIC** ^**Bu**^ does not contain additional BD, it is no surprise that its Δε values are almost independent of the temperature. Moreover, we complemented the dielectric measurements with stress relaxation experiments via rheology at various temperatures to further verify that the origin behind this dielectric process is associated with the dynamic bond exchange. Indeed, there is a strong overlap between the two techniques regarding the bond exchange relaxation, as presented in the relaxation map (Figure 4e). It is also apparent that the Arrhenius fit used to describe the dielectric relaxation time also fits adequately the rheological data. Additional information regarding the stress relaxation measurements is presented in the Supporting Information (Figures S48 and S49). The temperature dependence of segmental relaxation also appears to depend strongly upon annealing. The segmental relaxation times of the fresh sample exhibit a clear shift to lower temperatures by ∼15 K compared to the annealed sample, attributed to lower cross-linking density prior to topological rearrangement. Finally, at low temperatures (in the range of −50 °C to −10 °C) in both Figure 4d,e, the β-relaxation is observed and is attributed to the rotation of the pendant carboxymethyl groups around the main chain^62^ and shows no significant variation between samples, indicating identical chemical structure.

A relationship between the overall electrical conductivity, σ_0_, and charge transport related to the bond exchange relaxation is presented for **PMA-3%DIC** ^**Bu**^ **-0.15%BD** prior and postannealing (Figure 5). It is evident that post annealing, the σ_0_ values are within an order of magnitude with the charge transport associated with the bond exchange relaxation, indicating a correlation between the two phenomena, similar to what has been observed between conductivity and interfacial polarization in the literature.^64,65^ The observed correlation suggests that the induced dipole arising from the intermediate shown in Figure 3a facilitates charge transport; i.e., the mobility of charge carriers partially relates to the bond exchange relaxation. On the other hand, the fresh **PMA-3%DIC** ^**Bu**^ **-0.15%BD** sample exhibits significant differences, also corroborated by the σ_0_ values and temperature dependence presented as an inset in Figure 5. To understand the underlying factors at play, we first need to remember that charge transport is often coupled with segmental dynamics^66^ and that electrical conductivity is the product of the mobility, μ, and number density, *n*, of charge carriers with the elementary charge (σ = *n*μq_*e*_). Therefore, the observed variation between conductivity values with annealing should be attributed to changes in the mobility of the charge carriers. Most importantly, as discussed earlier, prior to annealing, the sample has not undergone topological rearrangement, resulting in lower cross-linking density, which accelerates the segmental dynamics (Figure 4e), which in return increases the mobility of charge carriers. Moreover, it appears that the fresh sample exhibits decoupling between σ_0_ and bond exchange relaxation at high temperatures. Considering that the network’s cross-linking topology is out of equilibrium, we attribute this disparity to mobile charge carriers that move independently from the bond exchange relaxation. The proposed mechanism regarding the coupling of electrical conductivity with the bond exchange relaxation provides a new dimension toward understanding vitrimer polymers and how their physical properties relate to their unique dynamic characteristics.

**Figure 5:**
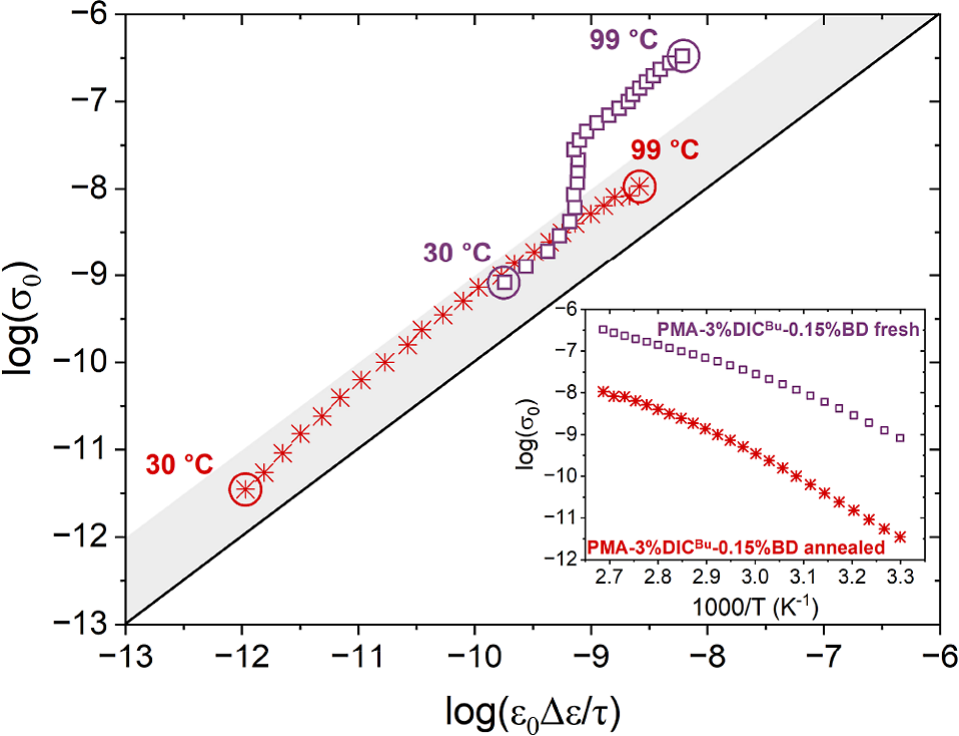
Scaling of the electrical conductivity with the bond exchange relaxation dipolar characteristics (both given in S/m). The electrical conductivity σ_0_ was calculated from the frequency-dependent dielectric response (eq S8), while the Δε and τ values of the bond exchange relaxation are presented via the Barton, Nakajima, and Namikawa (BNN) eq (eq S9).^63^ The straight black line corresponds to a 1-1 relationship between the two values, and the shaded area indicates values that are within an order of magnitude from the 1-1 relationship. The inset shows electrical conductivity as a function of reciprocal temperature.

Finally, considering the ability of vitrimers to self-heal when subjected to heat,^67^ they could offer an attractive option for dielectric materials such as capacitor devices with longer cycle life after electrical damage,^68^ electronic packaging,^69^ or dielectric elastomer actuators and energy harvesters.^70^ For that reason, the real part of dielectric permittivity and the dielectric loss tangent (tanδ_BDS_ = ε″/ε*′* ^42^) both as a function of temperature are presented at 10 kHz, i.e., a frequency related to power conditioning capacitor applications that are used in voltage stabilization^71,72^ (Figure S59). It is apparent that the vitrimer samples **PMA-2%DIC** ^**Bu**^ and **PMA-2%DIC** ^**Bu**^ **-0.10%BD** show a variation on dielectric permittivity values with temperature that ranges from 3.5 to 7.5, which is relatively high for plain polymers, highlighting that the developed poly­(acrylate-*co*-isocyanide) vitrimers are promising candidates for dielectric applications (Figure S59a). In the vicinity of the *T*_g_, a peak is observable, which is ascribed to the segmental relaxation discussed earlier. The dielectric losses we observe maintain relatively low values of tanδ_BDS_ < 0.1 even at the peak of the segmental relaxation and even lower values at higher temperatures (tanδ_BDS_ ∼ 0.03) (Figure S59b), despite that *T*_g_ generally facilitates electrical conduction and mechanical failure in amorphous polymer capacitors.^73^

## Conclusion

In this study, we successfully developed poly­(acrylate-*co*-isocyanide) vitrimers utilizing multiisocyanides as cross-linkers, offering a straightforward and cost-effective strategy for vitrimer synthesis. Our results demonstrate that these materials possess high cross-linking density, reprocessability, and superior mechanical properties. This method enables rough control over the cross-linking density by varying the loading of isocyanide cross-linkers, demonstrating its potential for tailoring desired vitrimer properties. Additionally, the structure of the cross-linkers was also shown to be tunable, although constrained by the steric hindrance of the isocyanides. Free amine was also proven to be an essential additive to facilitate associative bond exchange in this network. To elucidate the relaxation charge transport dynamics varying cross-linking density and the annealing history of **PMA-DIC** ^**Bu**^ vitrimers, BDS was employed. The temperature dependence of the segmental relaxation process showed faster dynamics, suggesting that the dynamic bonds facilitate segmental motions as well as that unincorporated DIC^Bu^ acts as a plasticizer. Dielectric measurements and the subsequent relaxation dynamics showed that after overnight thermal treatment at temperatures higher than the *Tv*, topological rearrangement increased the cross-linking density and thus slowed the segmental dynamics. At high temperatures, a dipolar process associated with dynamic bonds (bond-exchange relaxation) was identified, exhibiting a relatively high activation energy (*E*_a_ = 108.2 kJ mol^–1^) compared with common vinylogous urethane bond exchange. This higher value is attributed to steric hindrance at the reactive centers, which retards the bond-exchange process. Finally, the electrical conductivity was found to correlate with bond exchange relaxation, scaling over a broad temperature range, thus indicating that dynamic bonds are the underlying mechanism behind charge transport. Consequently, the incorporation of dynamic linkages enhances the dielectric permittivity of the material. The aforementioned mechanisms indicate the complex nature of dynamic bonds in vitrimer polymers and how the underlying molecular processes affect their physical properties.

## Supplementary Material



## Notes

The Supporting Information contains experimental methods, data, and photographs of this work. The Supporting Information is available free of charge at https://pubs.acs.org/doi/10.1021/jacs.5c22502.
