# Supplementary material for: Polyacrylate Vitrimer Network via In Situ Isocyanide Copolymerization: Synthesis and Molecular Dynamics
Source: J Am Chem Soc. 2026 Mar 6;148(10):11047–57. doi: 10.1021/jacs.5c22502 (PMC13003504; doi:10.1021/jacs.5c22502)
Supplement: Supplementary file 1 [file ja5c22502_si_001.pdf]

Polyacrylate Vitrimer Network via In-Situ Isocyanide Copolymerization: Synthesis and Molecular Dynamics

Han-Li Sun[1§], Stavros X. Drakopoulos[2§], Lejla Čamdžić[1,3], Shawn M. Maguire[4], Rodney D. Priestley*[2,4], Erin E. Stache*[1]

[1]Department of Chemistry, Princeton University, Princeton, New Jersey 08544, United States

[2]Princeton Materials Institute, Princeton University, Princeton, New Jersey 08544, United States

[3]Department of Chemistry and Chemical Biology, Cornell University, Ithaca, New York 14853, United States

[4]Department of Chemical and Biological Engineering, Princeton University, Princeton, NJ, 08540 United States

[§]The authors contributed equally

I. General Information 3

Materials 3

Instrumentation 3

II. Synthesis of isocyanides and monomers..5

Isocyanides synthesis 5

III. Synthesis and characterization of vitrimer samples 10

Procedures 10

Supplementary results 12

Characterization of isocyanide-acrylate copolymer networks (Figure 2d) 14

Incorporation analysis 25

Synthesis of linear PMA and PMA-2%diacrylate 29

Synthesis of PMA-DIC^{Bu}-BD 31

IV. Thermal, viscoelastic, and tensile properties..33

TGA analysis 33

Evaluation of Plasticizer Effect 33

Supplementary DMA analysis 35

Tensile test results 37

V. Supplementary BDS data 40

VI. References 52

I. General Information

Materials

Methyl acrylate (MA; Sigma-Aldrich, 99%) for photocuring: purified by drying over calcium hydride overnight, followed by distillation under nitrogen and degassing by three cycles of freeze-pump-thaw before being transferred to the glovebox; For 1-dram reaction: purified by passing through a column of basic alumina. $CDCl_3$ (Cambridge Isotope Laboratories Inc., 99.8%); Tetrahydrofuran (THF, ACS grade, Sigma-Aldrich); Hexanes (ACS grade, Fisher chemical); MeOH (ACS grade, Fisher chemical); Dichloromethane (ACS grade, Fisher chemical); Tris(2-phenylpyridine)iridium ($Ir(ppy)_3$, ChemScene, > 99%); 2,2'-Azobis(2-methylpropionitrile) (AIBN, Sigma-Aldrich, 97%): purified by recrystallization; Phenyl-bis(2,4,6-trimethylbenzoyl)phosphine oxide (BAPO, TCI, 98%), 2,6-Bornanedione (CQ, Sigma-Aldrich, 99%); n-Butyl isocyanide (BI, Fischer Scientific); Phosphorus oxychloride ($POCl_3$, Sigma-Aldrich, 99%); 1,4-Diaminobutane (Sigma-Aldrich, 97%); 1,3-Bis(aminomethyl)cyclohexane (cis/trans mixture, CAS: 2579-20-6, Oakwood); 1,4-Bis(aminomethyl)cyclohexane (cis/trans mixture, CAS: 2549-93-1, Oakwood); (1R,2R)-1,2-Diaminocyclohexane (Oakwood); Tris(2-aminoethyl amine) (Sigma-Aldrich, 97%); 1,1-Dimethylethylenediamine (Sigma-Aldrich, 97%); Ethyl formate (Sigma-Aldrich, 97%), *p*-Toluenesulfonic acid monohydrate (Acros Organics, 97.5%), 1,4-diaminobutane (BD, Sigma-Aldrich, 98%).

Instrumentation

Free radical polymerization reactions were irradiated using Chanzon LED chips equipped with TX Aluminum Heatsink Cooling Fan and wired with a 59.5 W band connector purchased from Mean Well. Each piece of the light set up was individually purchased from Amazon and wired manually. Light intensity was measured to be ~10 W at 575 nm by a THORLABS photometer with a 2.5 cm diameter round probe. PET-RAFT reactions were carried out under 427 nm (50 W) light irradiation using a Kessil PR160. Photo-strengthening of the materials were carried out under 370 nm (50 W) light irradiation using a Kessil PR160. Molecular weight (M_n) and dispersity ($Đ$) of the polymers were measured using gel permeation chromatography (GPC). A Waters ACQUITY Advanced Polymer Chromatography (APC) system equipped with a quaternary solvent manager and three APC columns in series (ACQUITY APC XT BEH column, 45 Å, 1.7 µm, 4,6 mm x 150 mm; ACQUITY APC XT BEH Column, 125 Å, 2.5 µm, 4.6 mm × 150 mm; ACQUITY APC XT BEH Column, 450 Å, 2.5 µm, 4.6 mm × 150 mm) using an ACQUITY RI detector with tetrahydrofuran as the eluent (flow rate = 0.5 mL/min) at 35°C was used. The GPC instrument was calibrated using polystyrene (PS) narrow standards. PMEA (Figure S21) sample was performed using an Agilent 1260 Infinity II Isocratic Pump with a 1260 Infinity II Refractive Index detector, equipped with two SDV Linear M columns (8 × 300 mm, 3 µm)

and one SDV guard column (8 × 50 mm, 3 μm). *N*, *N*-Dimethylacetamide (DMAc) was used as the eluent at a flow rate of 1 mL/min and a column temperature of 35 °C. Nuclear magnetic resonance (NMR) spectra were recorded on a Bruker 500 MHz at room temperature using *d*-chloroform ($CDCl_3$) as a solvent. Solid-state NMR spectra were recorded on a Bruker 600 MHz. The samples were packed into 3.1 mm zirconia rotors and measured under magic-angle spinning at 10 kHz. Infrared (IR) spectra were acquired on an Agilent Cary 630 FTIR spectrometer. Dynamic mechanical analysis (DMA) results were tested on a PerkinElmer DMA-8000 dynamic mechanical analyzer, with a rectangle test bar in tension mode. Each rectangular-shaped sample was heated from -10 to 100 °C at a rate of 3 °C/min. Sample dimensions were kept consistent as length x width x thickness measured approximately (20 mm x 5 mm x 0.60 mm). All experiments were run at a frequency of 1 Hz. All shear rheology tests were carried out using an Anton Paar MCR 501 Rheometer in parallel plate configuration. For stress relaxation experiments, 0.25 N normal force was applied to begin each run. The sample was allowed to equilibrate at the given temperature for >5 minutes before data were collected. Stress relaxation experiments were executed at 0.5% strain. Differential Scanning Calorimetry (DSC) was employed to measure the glass transition temperature of the samples. The measurements were performed using a differential scanning calorimeter 2500 provided by TA Instruments, equipped with an RCS90 cooler, under constant flushing with nitrogen. The DSC protocol included a sequence of heating – cooling – heating rates of 10 °C/min, in the temperature range of -50 °C to 100 °C. Thermogravimetric analysis (TGA) measurements were performed on PerkinElmer TGA-GC/MS: TGA-8000 thermogravimetric analyzer. Samples were heated under a nitrogen atmosphere at a rate of 10 °C/min from 25 to 500 ºC. The dielectric response of the PMA-based sampes was measured by means of Broadband Dielectric Spectroscopy (BDS). The BDS system was provided by Novocontrol Technologies (Germany) and consists of a frequency analyzer, a temperature controller, and a dielectric cell. The frequency analyzer is an Alpha-A high performance modular measurement system. The temperature control is achieved by the PHECOS system which has an integrated dielectric cell in which the sample is inserted in a parallel-plate capacitor configuration. Isothermal frequency scans were performed from -48 °C to 99 °C at 3 °C intervals with a ±0.1 °C accuracy. The applied voltage V_{rms} was maintained at 1 V and examined in a frequency range from 10^{-2} to 10^{6} Hz. Shredded samples were reprocessed on a DABPRESS hot-press with a steel compression mold at 80 ºC, 55 atm for 18 hours. Uniaxial tensile measurements were performed with a 3 mm/min strain rate at 25 °C, using a dumbbell-shaped film with an effective gauge dimension: 22 mm (L) × 5 mm (W), the thickness of 0.4~0.6 mm. Each result was the average value from testing of at least three samples.

II. Synthesis of isocyanides and monomers

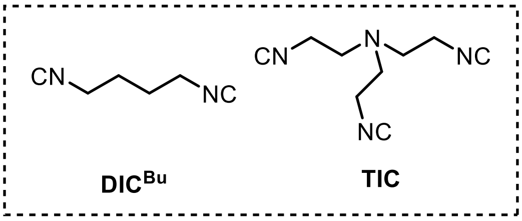

1,4-diisocyanobutane (**DIC^{Bu}**) [1] and Tris(2-isocyanoethyl)amine (**TIC**) [2] were prepared according to literature procedures.

Isocyanides synthesis

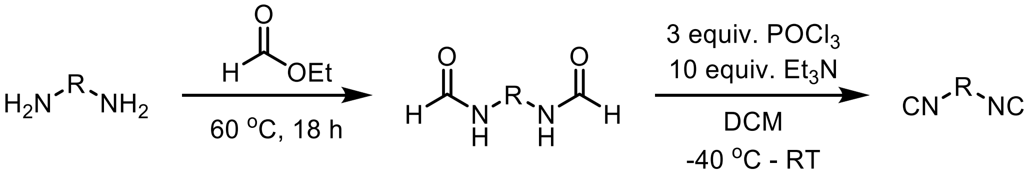

General procedure A: A solution of diamine (10 mmol) in 5 mL of ethyl formate was refluxed for 18 h and then concentrated to dryness under reduced pressure. The resulting product (formamide) was suspended in 30 mL of dry CH_2Cl_2, then NEt_3 (10 equiv., 100 mmol, ~18 mL) was added and the solution was cooled to -40 °C. $POCl_3$ (30 mmol, 3 equiv., 4.2 mL) was added dropwise *via* syringe during 30 minutes under N_2 atmosphere and the resulting reaction mixture was stirred overnight at room temperature. The mixture was poured into cold water (100 mL) (**CAUTION: This step is highly exothermic and can cause violent splashing of liquids!**) and extracted with CH_2Cl_2 (2 x 50 mL). The organic layer was washed with sat. aq. $NaHCO_3$, dried over Na_2SO_4 and concentrated under reduced pressure to give a crude product, which was purified by column chromatography (hexanes/EtOAc = 1:1) to afford pure diisocyanide product. The products need to be sealed and stored at -10 °C.

DIC$^{1,4\text{-}MeCy}$: 1,4-bis(isocyanomethyl)cyclohexane (mixed isomer)

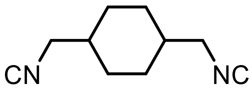

Following general procedure A. 1,4-Bis(aminomethyl)cyclohexane were used as starting material. 1.09 g product was obtained as a pale-yellow oil, overall yield 67%. ^{1}H NMR (500 MHz, $CDCl_3$) δ 3.64 – 3.22 (m, 4H), 2.10 – 0.95 (m, 10H). ^{13}C NMR (126 MHz, $CDCl_3$) δ 156.6, 47.6, 36.8, 25.3 (one isomer). ^{13}C NMR (126 MHz, $CDCl_3$) δ 156.6, 45.3, 34.7, 29.1 (another isomer). FTIR (cm^{-1}): 2933, 2863, 2147, 1454, 1359, 1217, 974, 952, 936, 922. MS-QTOF: $[M-H]^+$ calculated for $C_{10}H_{13}N_2$: 161.1073; Found: 161.1083.

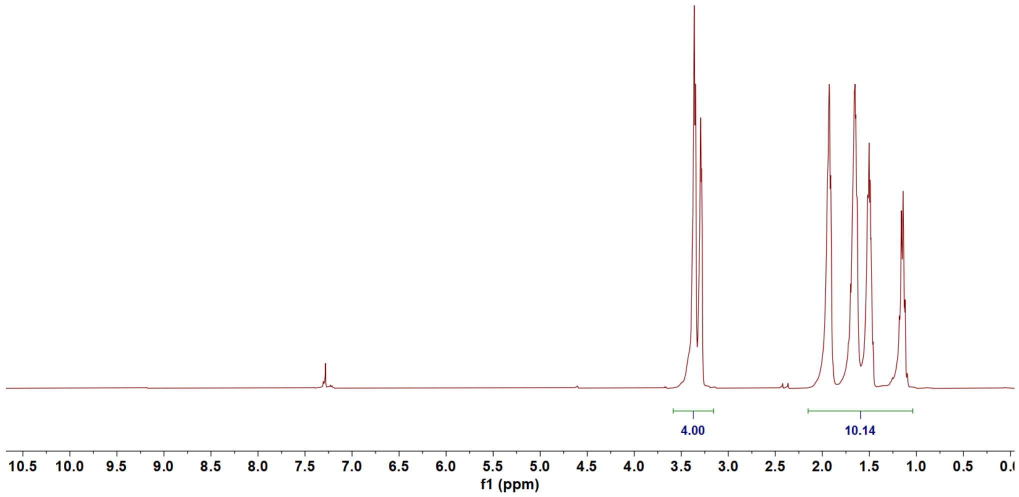

Figure S1. ^{1}H NMR of 1,4-bis(isocyanomethyl)cyclohexane (**DIC$^{1,4\text{-}MeCy}$**)

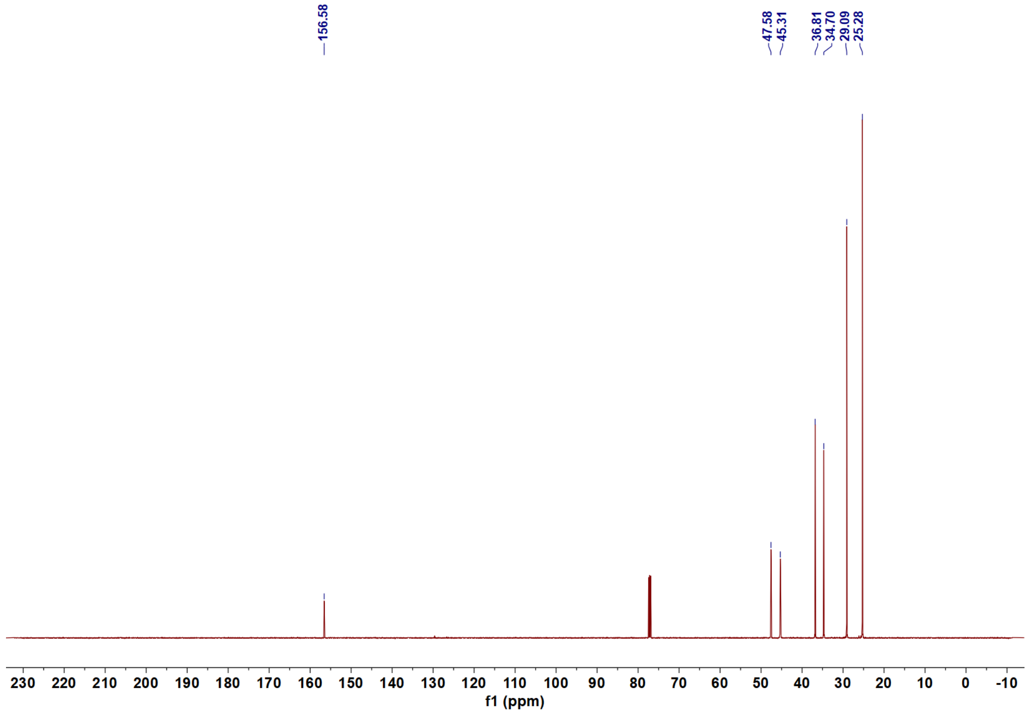

Figure S2. ^{13}C NMR of 1,4-bis(isocyanomethyl)cyclohexane (**DIC$^{1,4\text{-}MeCy}$**)

DIC$^{1,3\text{-}MeCy}$: 1,3-bis(isocyanomethyl)cyclohexane (mixed isomer)

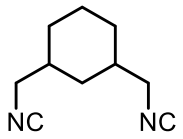

Following general procedure A. 1,3-Bis(aminomethyl)cyclohexane were used as starting material. 0.70 g product was obtained as a pale-yellow oil, overall yield 43%. ^{1}H NMR (500 MHz, $CDCl_3$) δ 3.46 – 3.20 (m, 4H), 2.13 – 0.70 (m, 10H). ^{13}C NMR (126 MHz, $CDCl_3$) δ 156.8, 47.7, 36.7, 33.4, 29.4, 24.6 (one isomer). ^{13}C NMR (126 MHz, $CDCl_3$) δ 156.8, 45.7, 32.4, 30.9, 28.2, 19.9 (another isomer). FTIR (cm^{-1}): 2931, 2859, 2148, 1450, 1360, 1349, 948, 934, 870, 855. MS-QTOF: $[M-H]^+$ calculated for $C_{10}H_{13}N_2$: 161.1073; Found: 161.1079.

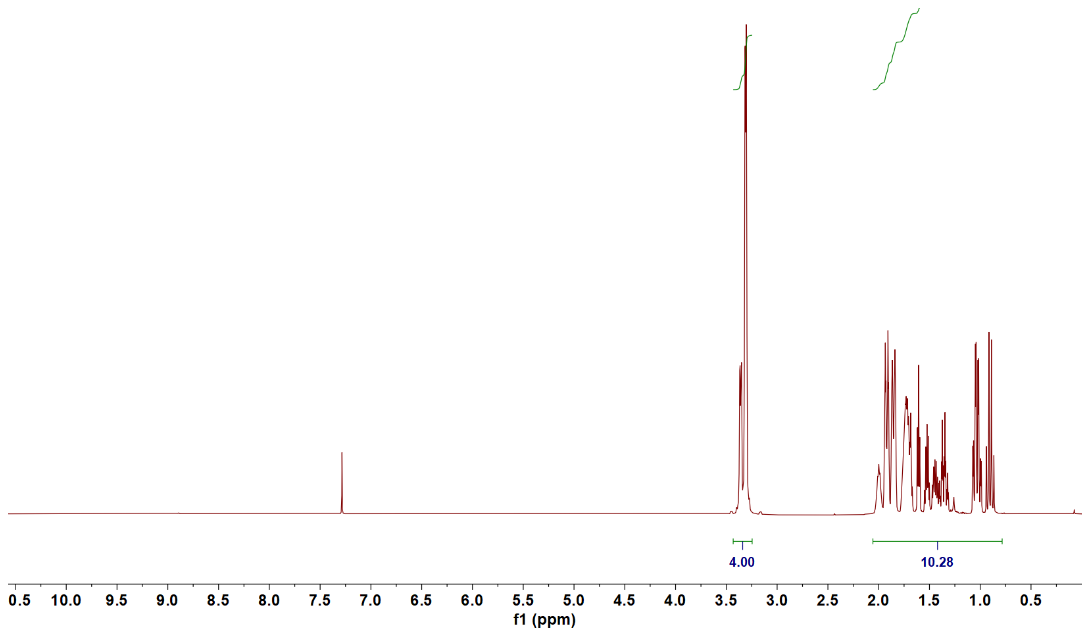

Figure S3. ^{1}H NMR of 1,3-bis(isocyanomethyl)cyclohexane (**DIC**$^{\mathbf{1,3\text{-}MeCy}}$)

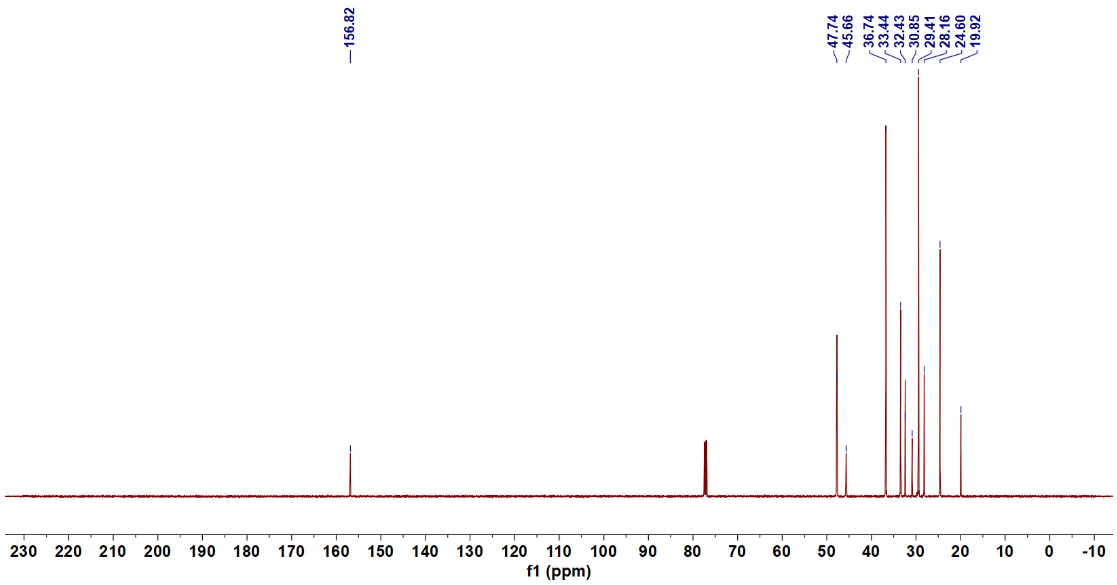

Figure S4. ^{13}C NMR of 1,3-bis(isocyanomethyl)cyclohexane (**DIC**$^{\mathbf{1,3\text{-}MeCy}}$)

DIC$^{1,2\text{-}Cy}$: (1R,2R)-1,2-diisocyanocyclohexane

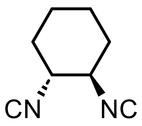

Following general procedure A. (1R,2R)-1,2-Diaminocyclohexane were used as starting material. 0.86 g product was obtained as a pale-yellow oil, overall yield 64%. ^{1}H NMR (500 MHz, $CDCl_3$) δ 3.70 (dd, J = 7.1, 3.4 Hz, 2H), 2.25 – 2.14 (m, 2H), 1.81 – 1.74 (m, 2H), 1.72 – 1.65 (m, 2H), 1.41 (m, 2H). ^{13}C NMR (126 MHz, $CDCl_3$) δ 158.9, 55.4, 30.1, 22.0. FTIR (cm^{-1}): 2949, 2870, 2142, 1452, 1370, 1166, 1049, 980, 909, 840. MS-QTOF: [M-H]$^+$ calculated for $C_8H_9N_2$: 133.0760; found 133.0768.

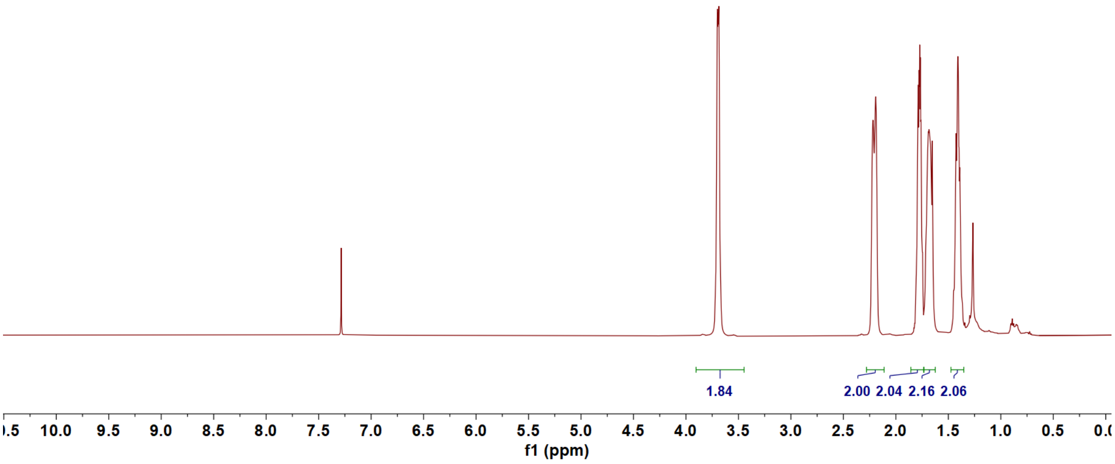

Figure S5. ^{1}H NMR of (1R,2R)-1,2-diisocyanocyclohexane (**DIC$^{1,2\text{-}Cy}$**)

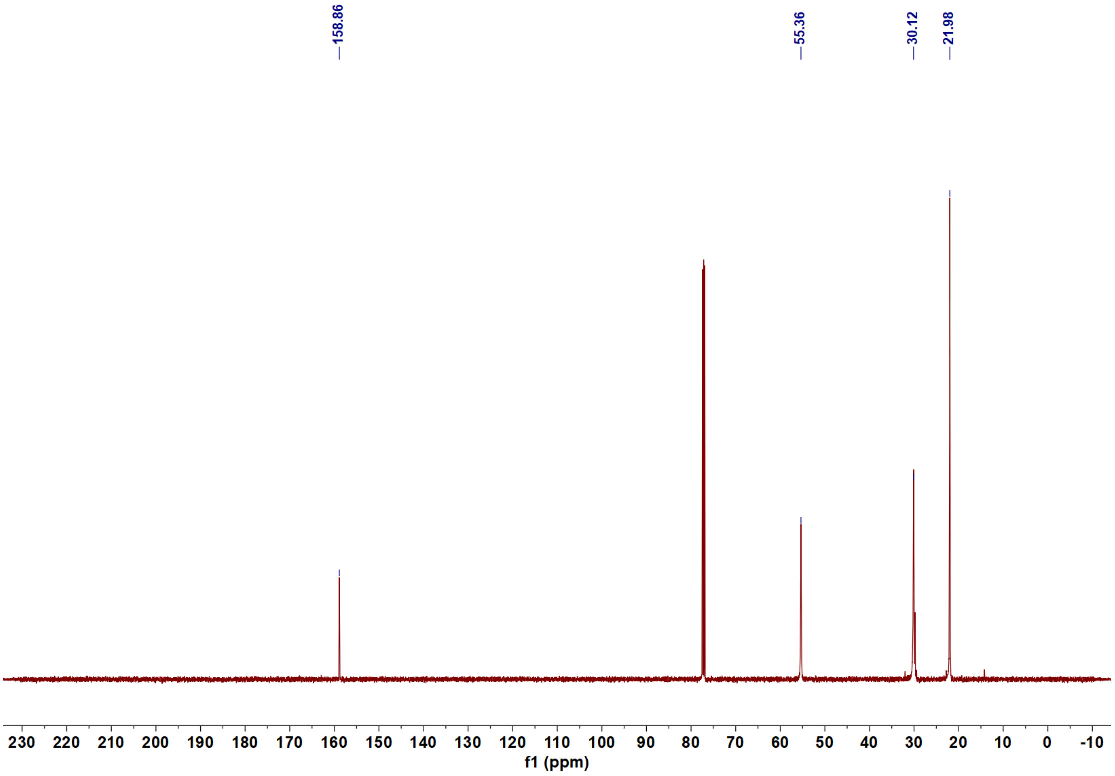

Figure S6. ^{13}C NMR of (1R,2R)-1,2-diisocyanocyclohexane (**DIC$^{1,2\text{-}Cy}$**)

DIC^{diMe}: 1,2-diisocyano-2-methylpropane

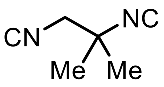

Following general procedure A. 2-Methylpropane-1,2-diamine were used as starting material. 108.1 mg product was obtained as a yellow oil, overall yield 10%.^{1}H NMR (500 MHz, $CDCl_3$) δ 3.56 (s, 1H), 1.58 (s, 4H). ^{13}C NMR (126 MHz, $CDCl_3$) δ 160.8, 157.9, 56.0, 52.0, 26.6. FTIR (cm^{-1}): 2995, 2154, 2136, 1747, 1739, 1730, 1470, 1453, 1399, 1293. MS-QTOF: [M-H]$^+$ calculated for $C_6H_7N_2$: 107.0604; found 107.0607. (*The low yield is due to the cyclization in formylation step, shown below*)

Possible side reaction:

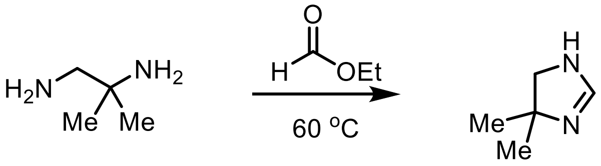

Found in crude GC-QTF

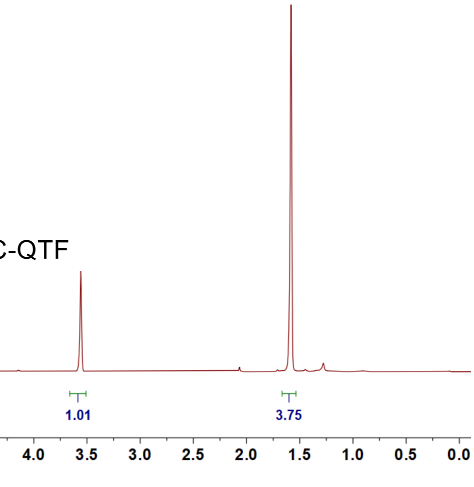

Figure S7. ^{1}H NMR of 1,2-diisocyano-2-methylpropane (**DIC^{diMe}**)

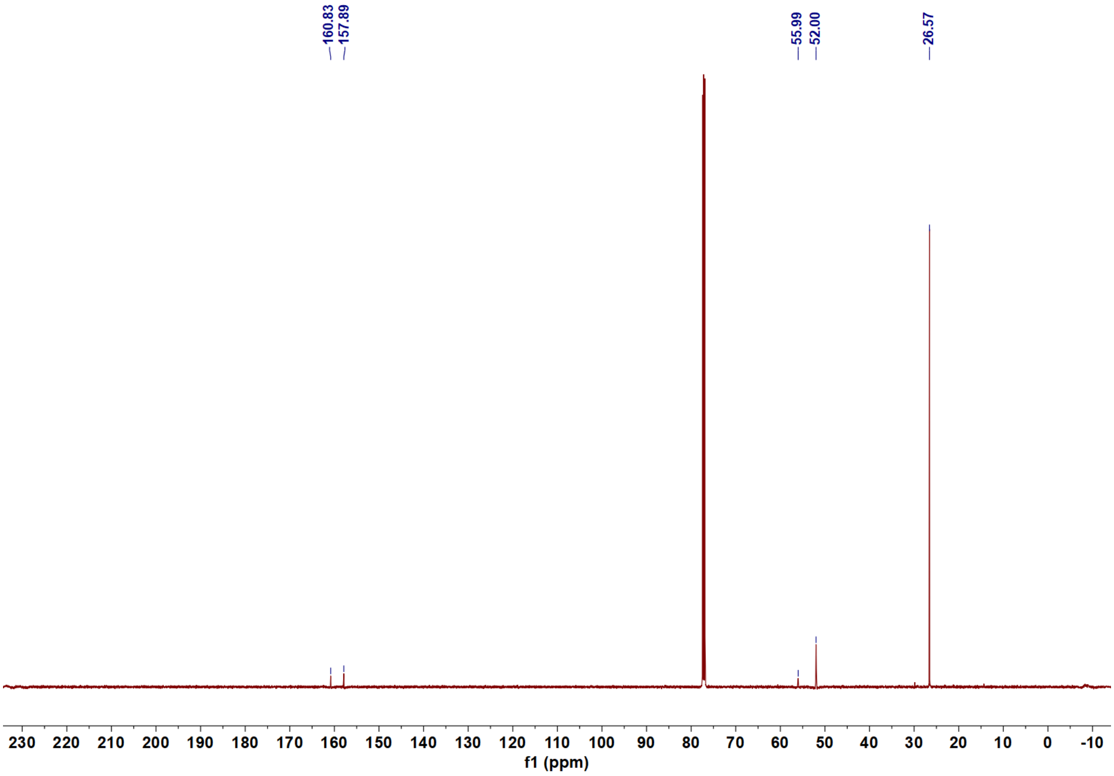

Figure S8. ^{13}C NMR of 1,2-diisocyano-2-methylpropane (**DIC^{diMe}**)

III. Synthesis and characterization of vitrimer samples

Procedures

MA + x mol% **DIC^{Bu}** $\xrightarrow[\text{white light}]{\text{y mol\% BAPO}}$ **PMA-DIC^{Bu}**

General procedure B (1-dram reaction): An oven-dried 1-dram vial was filled with BAPO, then isocyanide and acrylate monomer (4.0 mmol) were sequentially added using a micropipette. The vial was sealed with a rubber septum and subjected to three freeze-pump-thaw cycles at -78°C under nitrogen. After the reaction mixture was brought back to room temperature, the vial was irradiated from the bottom using a 50 W white LED for 10 hours. Once the reaction was finished, the cured sample was transferred out using spatulas and tweezers. A pale yellow, transparent, free-standing elastic disc can be obtained. The sample can be cut into small pieces for further analysis. This procedure was used for **Table 1** and **Figure 2**.

a)

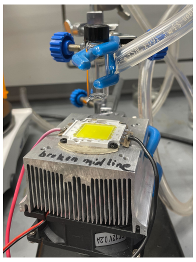

b)

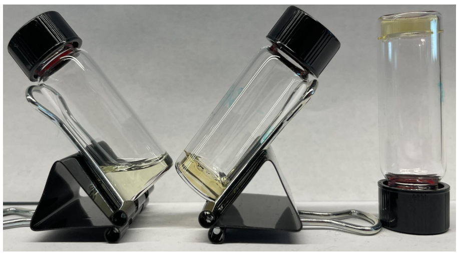

Figure S9. a) Set-up for 1-dram reaction. (~2.5 cm from LED chip to vial bottom)
b) Reaction vial before (left one) and after (right two) photo-curing.

General procedure C (for PET-RAFT): Adopted from **General procedure B**. The 1-dram vial was filled with CTA and $Ir(ppy)_3$ (0.25 mg, 0.0040 mmol, 0.1 mol%) instead of BAPO. The vial was irradiated from the bottom using a 50 W 427 nm Kessil LED.

This procedure was used for **Figure 2e**.

Procedure for determining swelling ratio (SR) / gel fraction (GF): 30-50 mg one-piece-sample was weighed ($\boldsymbol{M_o}$) into a vial and soaked in 5 mL organic solvent. The vial was then stirred on Vortex mixer. After 24 hours, the liquid phase was removed via syringe, then the sample can be taken out. After the surface solvent was wiped carefully, the swollen sample was weighed ($\boldsymbol{M_s}$). Then the sample was dried at 40 °C for 24 hours to a constant weight ($\boldsymbol{M_d}$). (For toluene, 70 °C was used)
Swelling ratio (**SR**) and gel fraction (**GF**) were calculated according to the following formula:

$$\mathbf{SR} = (M_s - M_o)/M_o$$

$$\mathbf{GF} = M_d/M_o$$

Procedure for hydrolysis/decrosslinking: A stir bar, 30-50 mg sample and ~10 mg $TsOH{\cdot}H_2O$ were transfer into a vial. 1 mL THF and 50 μL deionized water were the added via micropipette. The mixture was stirred at room temperature for 18 h, resulting in a clear solution. The solution was precipitated in cold MeOH (- 20 °C) once. Then the obtained polymer sample was dried under vacuum and then analyzed by gel permeation chromatography. (Table 1, entries 4, 5, 7, GPC were tested without precipitation)

General procedure D (for thin film sample): In glovebox, to a 2-dram vial, BAPO (25.1 mg, 0.06 mmol, 0.3 mol%), isocyanide, MA (1721.8 mg, 20.0 mmol, 1.0 equiv.) and free amine (if any) were added sequentially and mixed. After BAPO was completely dissolved, the reaction mixture can be transferred into an aluminum weighing plate (4.6 cm in diameter) via pipette. Then the reaction mixture was irradiated under white light for 10 hours, and a thin film (~0.6 mm thickness) can be obtained. The film can be cut into any desired shape using a punch cutter or a razor blade.

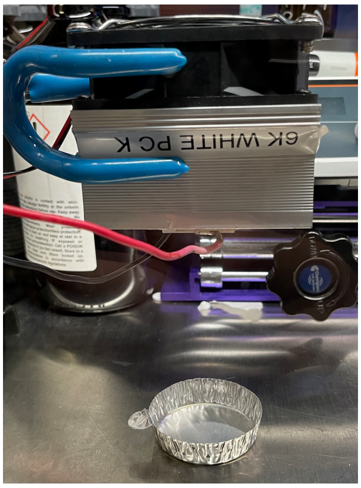

Figure S10. Set-up for thin film preparation (~8 cm from LED chip to sample plate).

Supplementary results

Table S1. Supplementary results of different initiators.

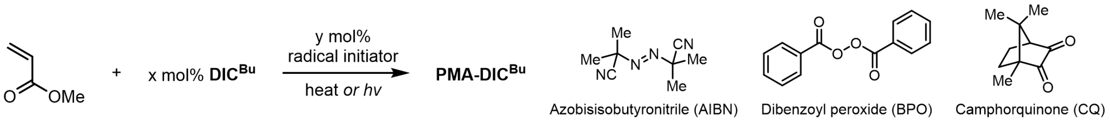

Entry	x	y	Initiator	*hv* (nm)	Temperature (°C)	$M_{n,\ hydrolysis}$ (kg/mol)	$Đ_{hydrolysis}$	Gel Fraction (%)
1	5	6	AIBN	-	70	6.1	1.9	91
2	10	1	BPO	-	70	4.8	1.4	49
3	5	0.3	CQ	456	RT	7.2	2.2	97
4	10	2	BAPO	390	RT	-	-	0

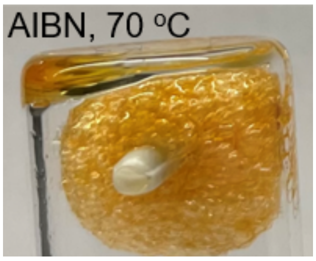

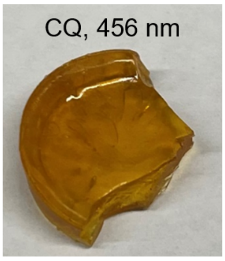

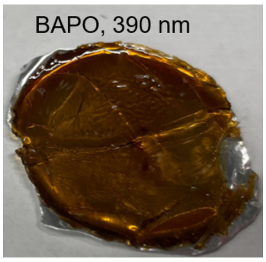

The sample initiated by AIBN exhibits porosity because of nitrogen gas evolution during polymerization. Samples prepared using camphorquinone (CQ) display no significant changes in chemical structure; however, they exhibit a darker appearance. Polymerization under 390 nm irradiation with BAPO does not lead to formation of cross-linked networks, but instead yields a dark, viscous gel.

Table S2. Control experiments with mono-isocyanides.

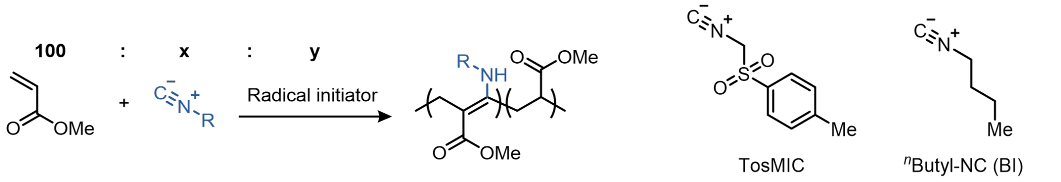

Entry	Isocyanide	ratio	Initiator	Condition	$M_{n,\ hydrolysis}$ (kg/mol)	$Đ_{hydrolysis}$	Gel Fraction (%)
1	TosMIC	100:10:6	AIBN	70 °C	7.0	1.6	0
2	TosMIC	100:30:6	AIBN	70 °C	4.7	1.6	0
3	TosMIC	100:60:6	AIBN	70 °C	<1.0	-	0
4	^{n}Butyl-NC (BI)	100:10:0.3	BAPO	White LED	11.8	2.3	0
5	^{n}Butyl-NC (BI)	100:6:0.3	BAPO	White LED	49.4	18	0
6	-	100:0:0.3	BAPO	White LED	221.8	1.7	0

Based on the results in Table S2, mono-isocyanide does not induce chain cross-linking. Moreover, the observed decrease in molecular weight with increasing isocyanide loading

supports the hypothesis that imidoyl radicals formed through isocyanide propagation are of higher energy, and thus more likely to undergo chain termination or transfer.

Transamination result

PMA-13%MEI was synthesized according to the reported method.[4] 258.2 mg of PMA-13%MEI and benzylamine ($BnNH_2$, 84.0 mg, 0.78 mmol, ~2.0 equiv. to enamine incorporation) were dissolved in dioxane and stirred at 60 °C for 24 hours. Then the solvent was evaporated under reduced pressure, and the crude product was precipitated three times with cold methanol. The resulting product was characterized by ^{1}H NMR spectroscopy.

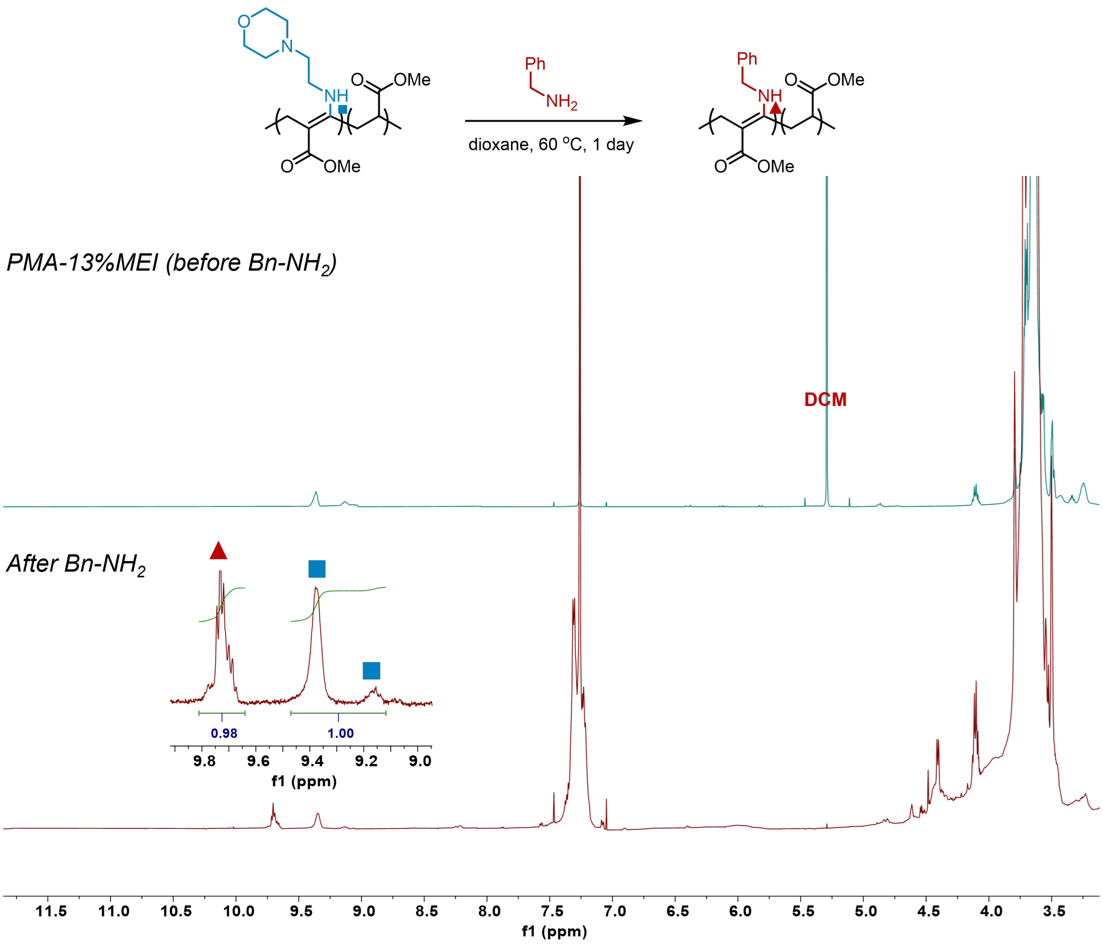

Figure S11. Stacked ^{1}H NMR before and after amine exchange.

Characterization of isocyanide-acrylate copolymer networks (Figure 2d)

Distinct signals corresponding to vinylogous urethane microstructures were observed in the IR spectra of all samples from P1 to P7, although the wavenumbers varied depending on the chemical structures of the respective monomers or cross-linkers. Additionally, the IR spectra of P3 and P4 exhibited more prominent signals attributable to unincorporated isocyanide.

PMA-3%SnatchCat (P1)

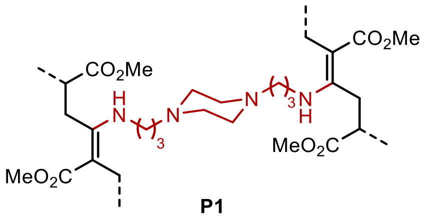

P1

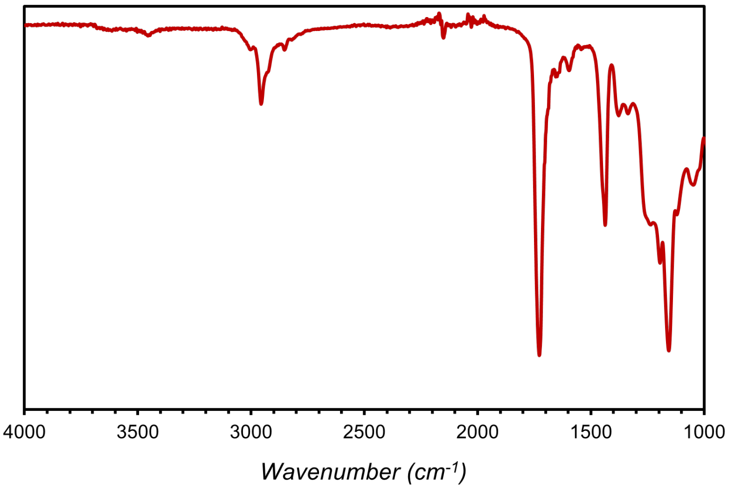

Figure S12. IR spectrum of P1.

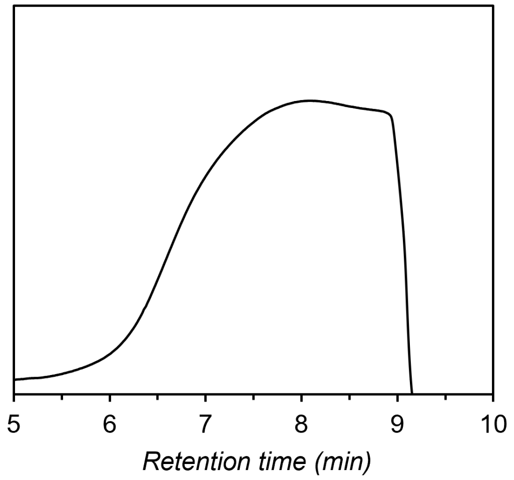

Figure S13. GPC trace after P1 hydrolysis.

PMA-2%TIC (P2)

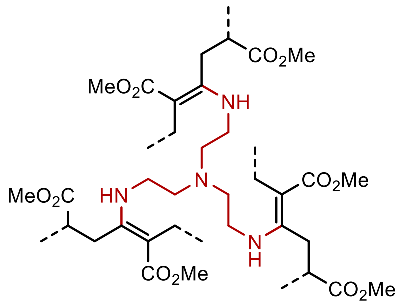

P2

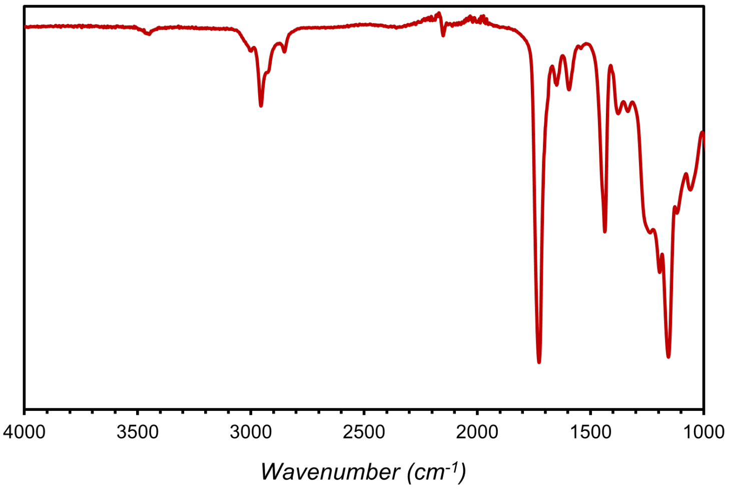

Figure S14. IR spectrum of P2.

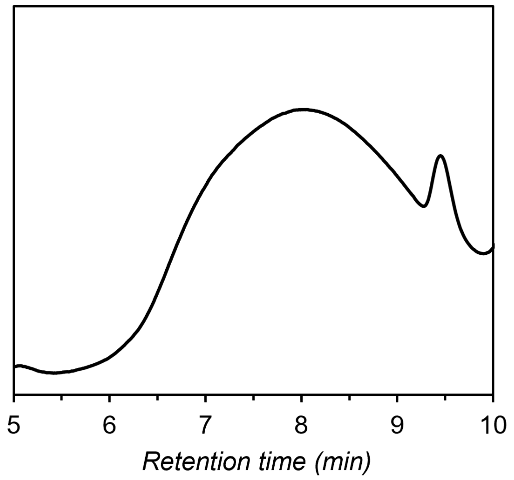

Figure S15. GPC trace after P2 hydrolysis.

PMA-3%DIC$^{1,4\text{-MeCy}}$ (P3)

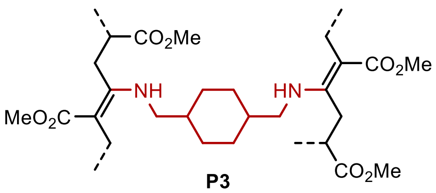

P3

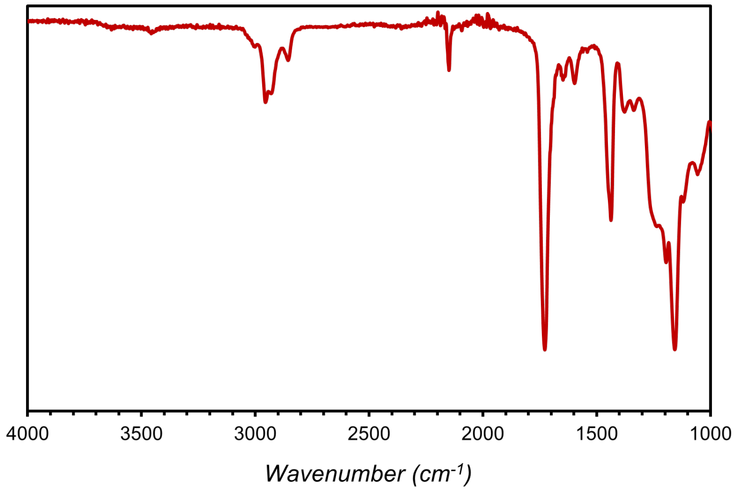

Figure S16. IR spectrum of P3.

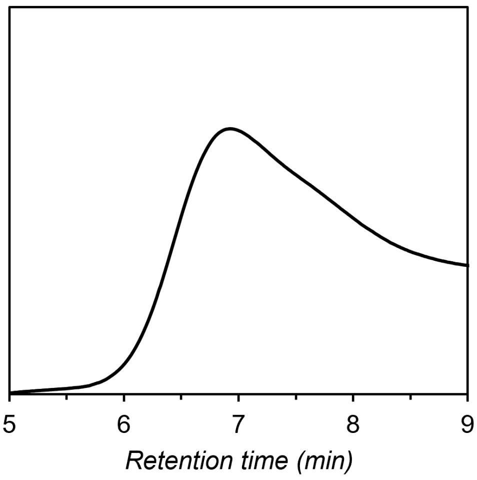

Figure S17. GPC trace after P3 hydrolysis.

PMA-3%DIC$^{1,3\text{-MeCy}}$ (P4)

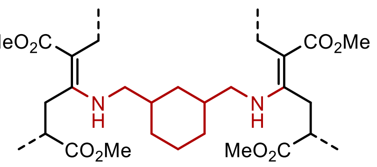

P4

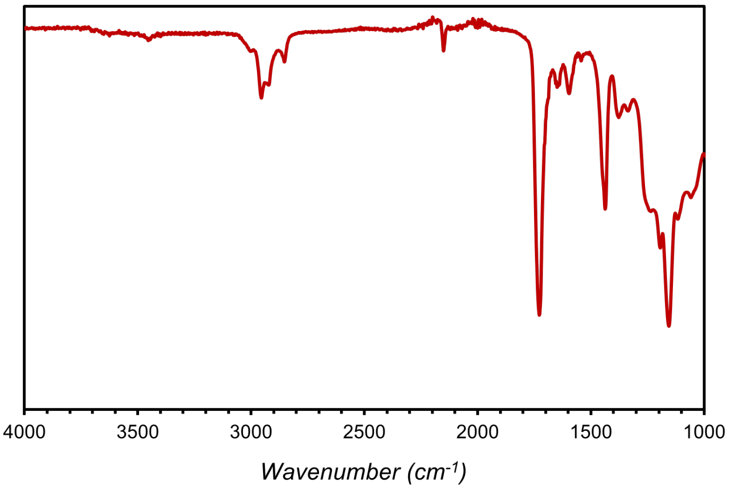

Figure S18. IR spectrum of P4.

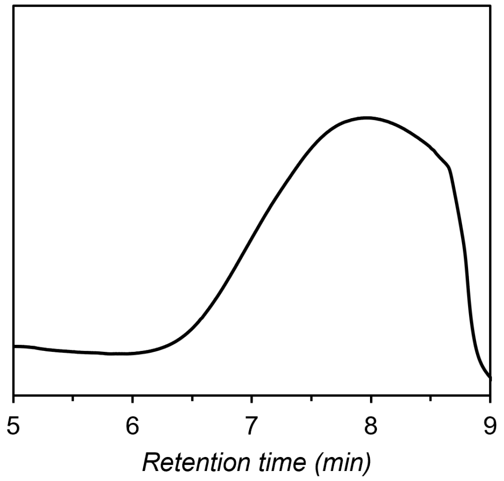

Figure S19. GPC trace after P4 hydrolysis.

PMEA-3%DIC^{Bu} (P5)

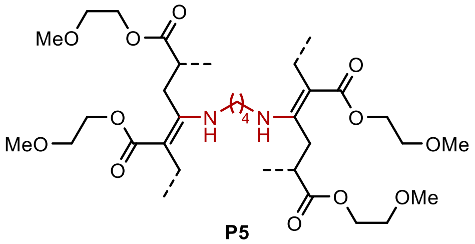

P5

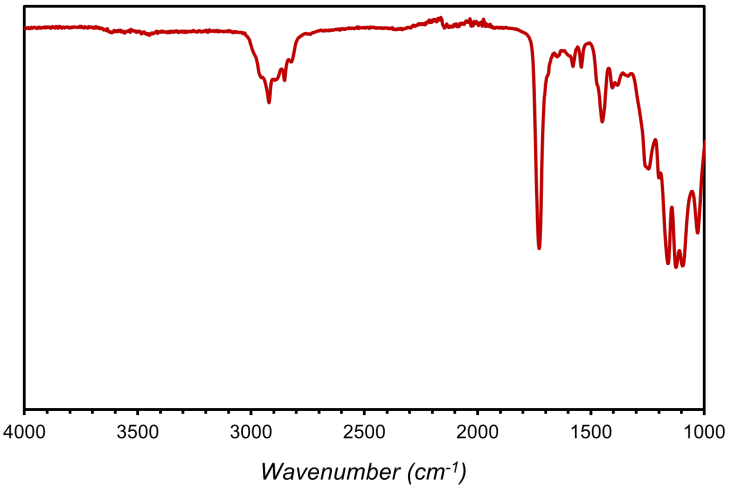

Figure S20. IR spectrum of P5.

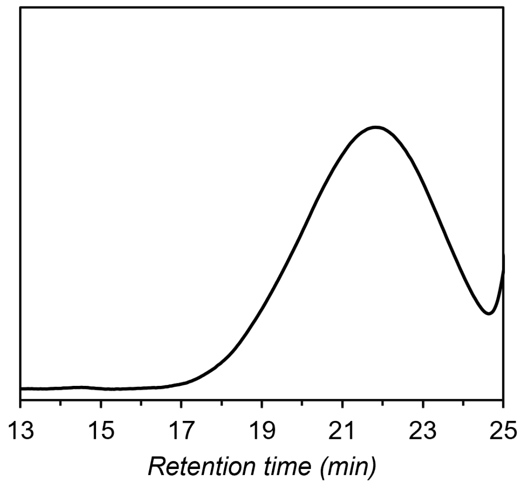

Figure S21. GPC trace after P5 hydrolysis.

PTFEA-3%DIC^{Bu} (P6)

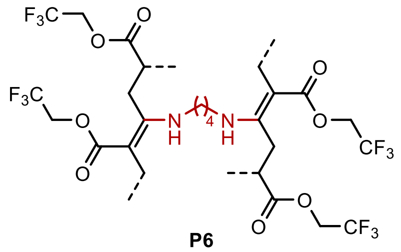

P6

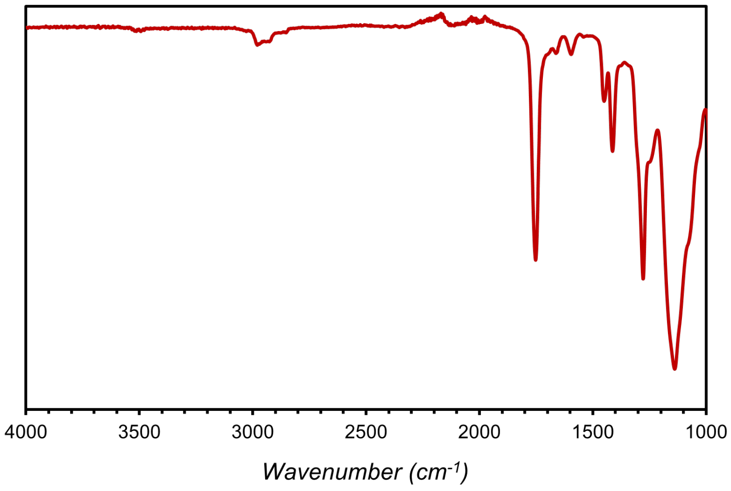

Figure S22. IR spectrum of P6.

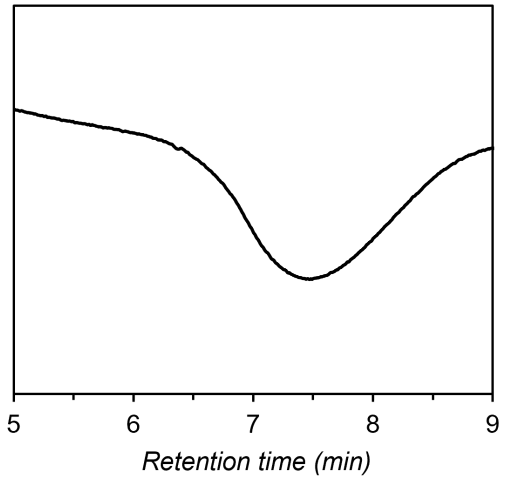

Figure S23. GPC trace after P6 hydrolysis.

PBnA-3%DIC^{Bu} (P7)

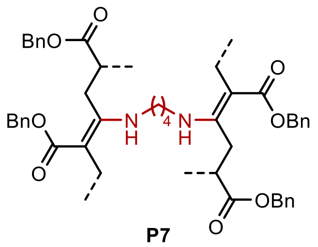

P7

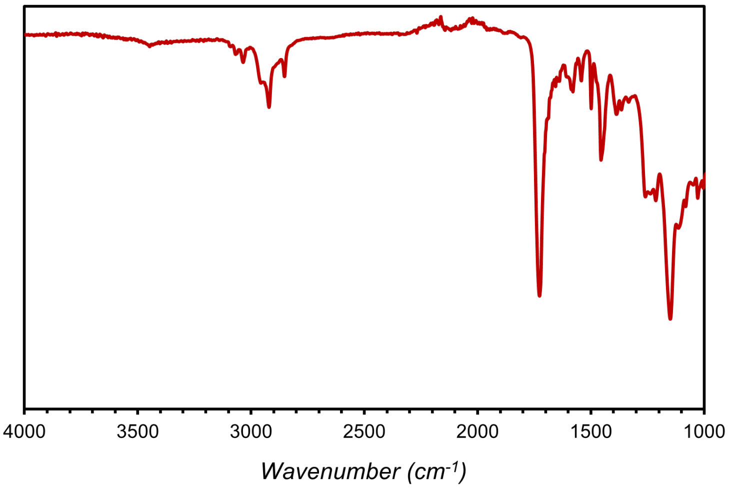

Figure S24. IR spectrum of P7.

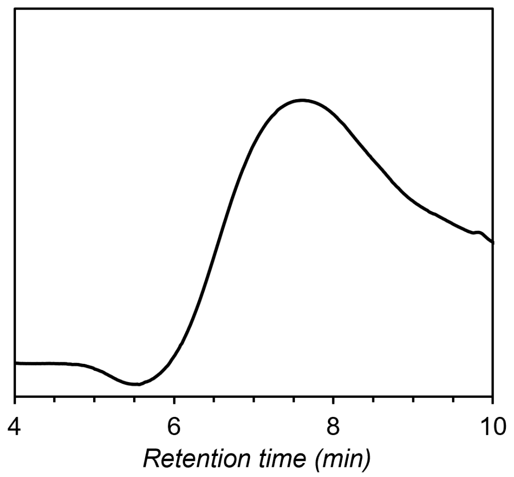

Figure S25. GPC trace after P7 hydrolysis.

Unsuccessful result (P8, P9)

The gel fractions of both P8 and P9 were found to be 0% in THF, DCM, and $CHCl_3$, suggesting the absence of crosslinked network formation. In the IR spectrum of P8, only weak enamine signals were observed alongside a strong residual isocyanide signal, implying that the incorporation efficiency of $DIC^{1,\,2\text{-}Cy}$ was significantly hampered by steric hindrance, thereby preventing the formation of network.

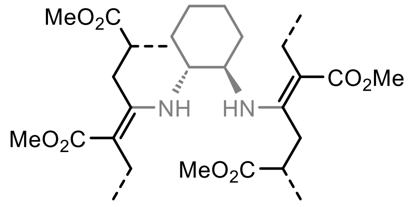

P8

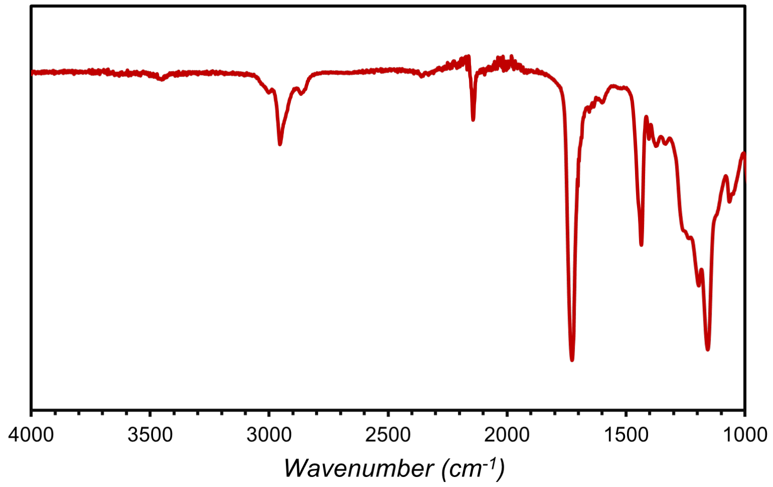

Figure S26. IR spectrum of P8

For P9, isocyanide peak was detected in IR or $^{1}H/^{13}C$ NMR spectra. Although DIC^{diMe} contains a spatially more accessible isocyanide group, after the formation of the enamine linkage, the β-position of the nitrogen remains sterically bulky. Such steric hindrance renders the enamine structure less stable and more likely to undergo hydrolysis in $CDCl_3$ (which typically contains water), leading to the formation of a polyketone species. Consequently, the isocyanide signal was not observed. In addition, a noticeable band appeared at 1600–1700 cm^{-1} in the IR spectra, consistent with the characteristic absorption of a polyketone structure. To further support this interpretation, DFT calculations on a small-molecule model (Section VI) indicate that the formation of an analogous enamine structure is thermodynamically unfavorable (endothermic by 3.3 kcal/mol).

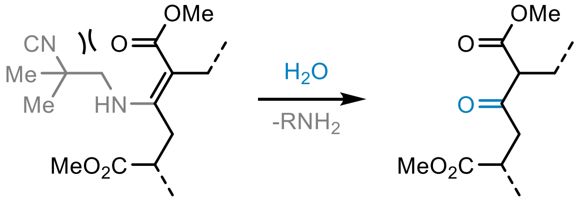

P9

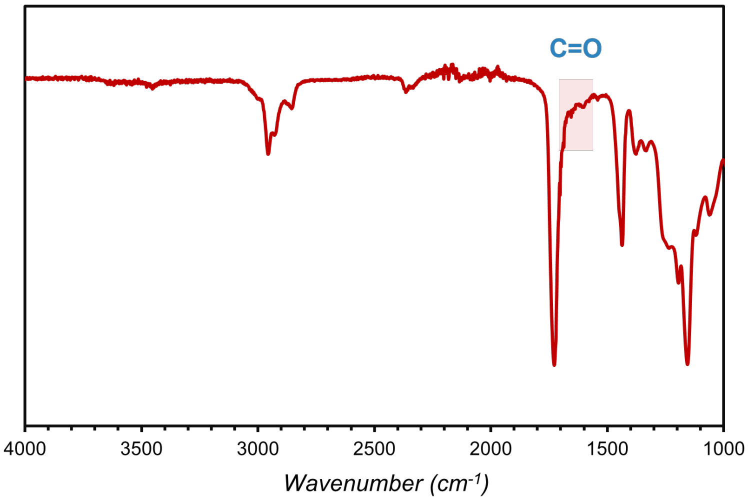

Figure S27. IR spectrum of P9.

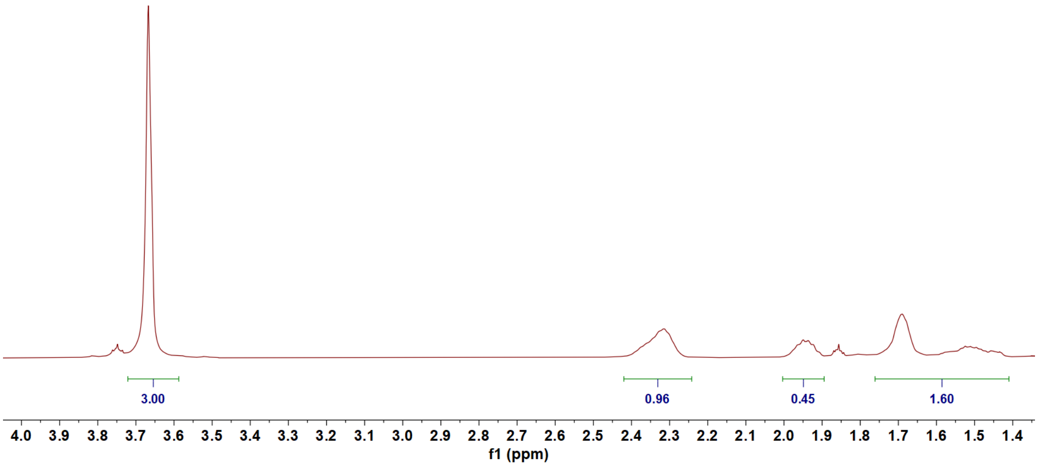

Figure S28. ^{1}H NMR of P9.

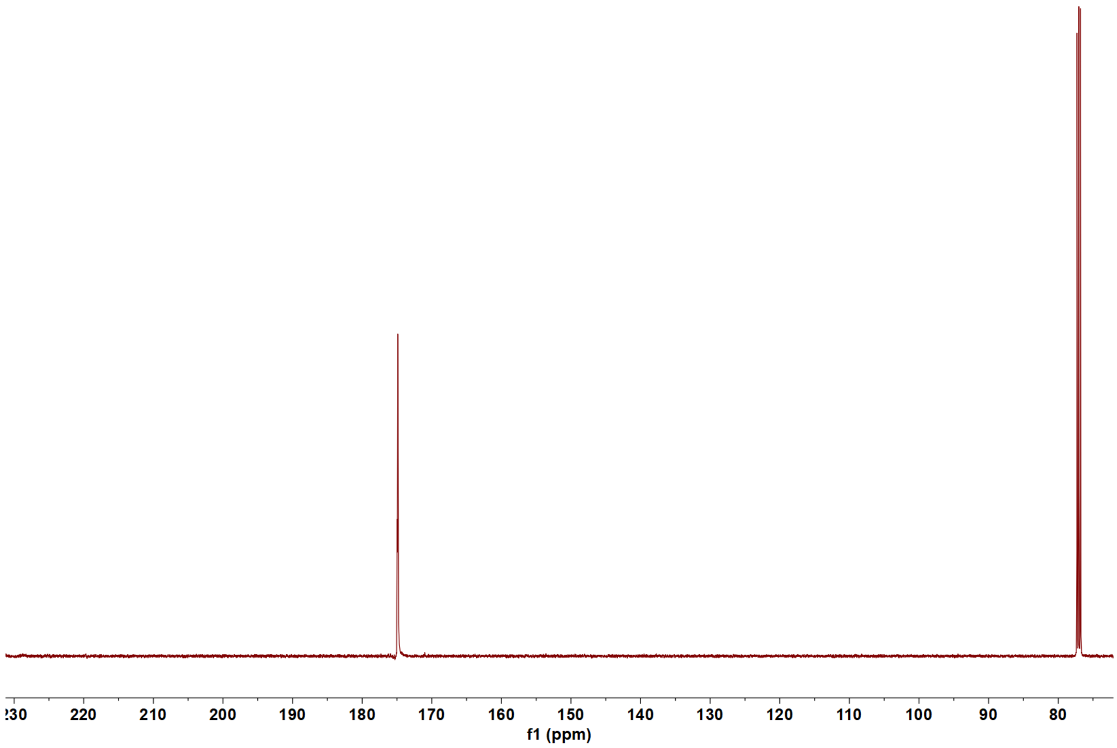

Figure S29. ^{13}C NMR of P9.

Photoinduced electron transfer-reversible addition-fragmentation chain transfer (PET-RAFT) polymerization (Figure 2e), following general procedure C for preparation.

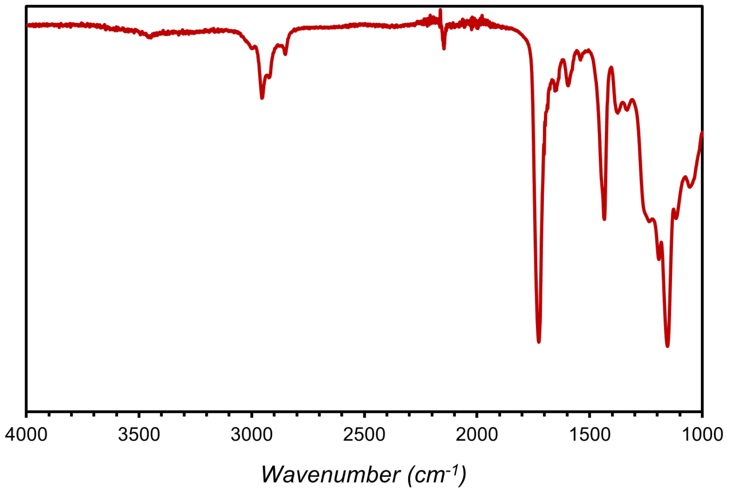

Figure S30. IR spectrum of PMA-5%DIC^{Bu} under PET-RAFT polymerization (CTA:MA = 200:1).

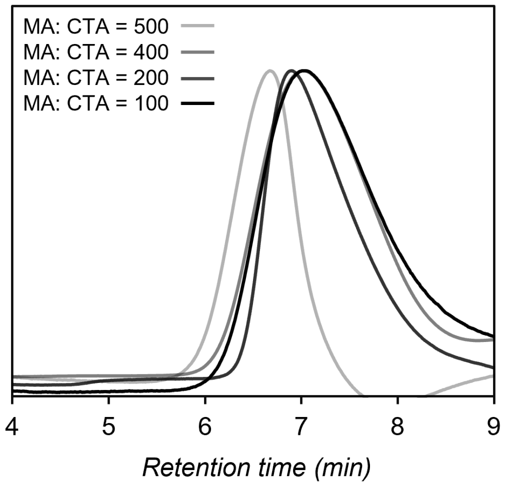

Figure S31. GPC traces PET-RAFT PMA-5%DIC^{Bu} sample after hydrolysis.

The cyclic voltammogram (CV) curve of DIC^{Bu} (ferrocene as external standard) is shown below. This electrochemical behavior suggests that DIC^{Bu} is not readily reduced by the excited state of Ir(ppy)$_3$* (**Ir(III)*/Ir(IV)= -1.73 V**) and can therefore be considered as electrochemically innocent under this PET-RAFT conditions.

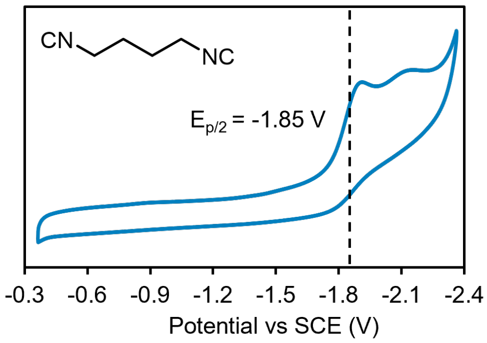

Figure S32. CV curve of DIC^{Bu}.

^{1}H NMR after hydrolysis

Hydrolysis was carried on with 50 mg sample. (**table 1, entry 1** as an example) The resulting solution was precipitated in cold MeOH and then re-dissolved in THF and precipitated in hexanes. The purified PMAK was dried under vacuum overnight. ^{1}H NMR spectroscopy was in good agreement with pervious literature.[3]

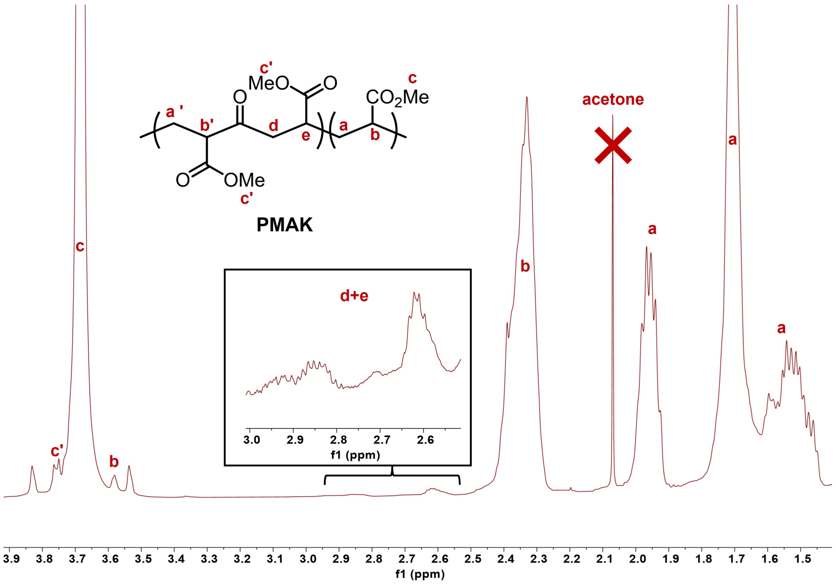

Figure S33. ^{1}H NMR after hydrolysis.

Incorporation analysis

IR reference: PMA-3.3% BI

PMA-3.3%BI was synthesized exactly as reported in the previous study under PI-RAFT conditions, and the obtained NMR spectra were consistent with the literature.[4] The incorporation was calculated based on the CH_3 (a) peak, as part of the CH_2 (d) peak of overlapped with the CH_3 (CO_2Me) peak. Additionally, HMBC experiments were performed to confirm the assignment of the CH_2 (b) signal, and its integration showed good correlation with that of the Me (a) signal.

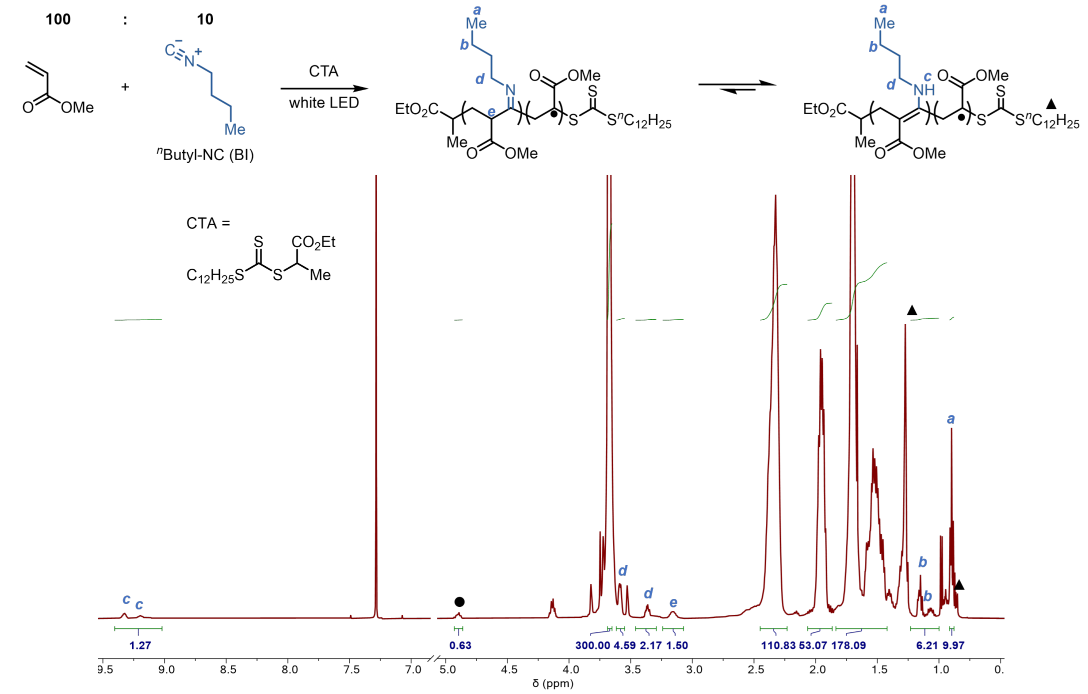

Figure S34. ^{1}H NMR of PMA-3.3%BI.

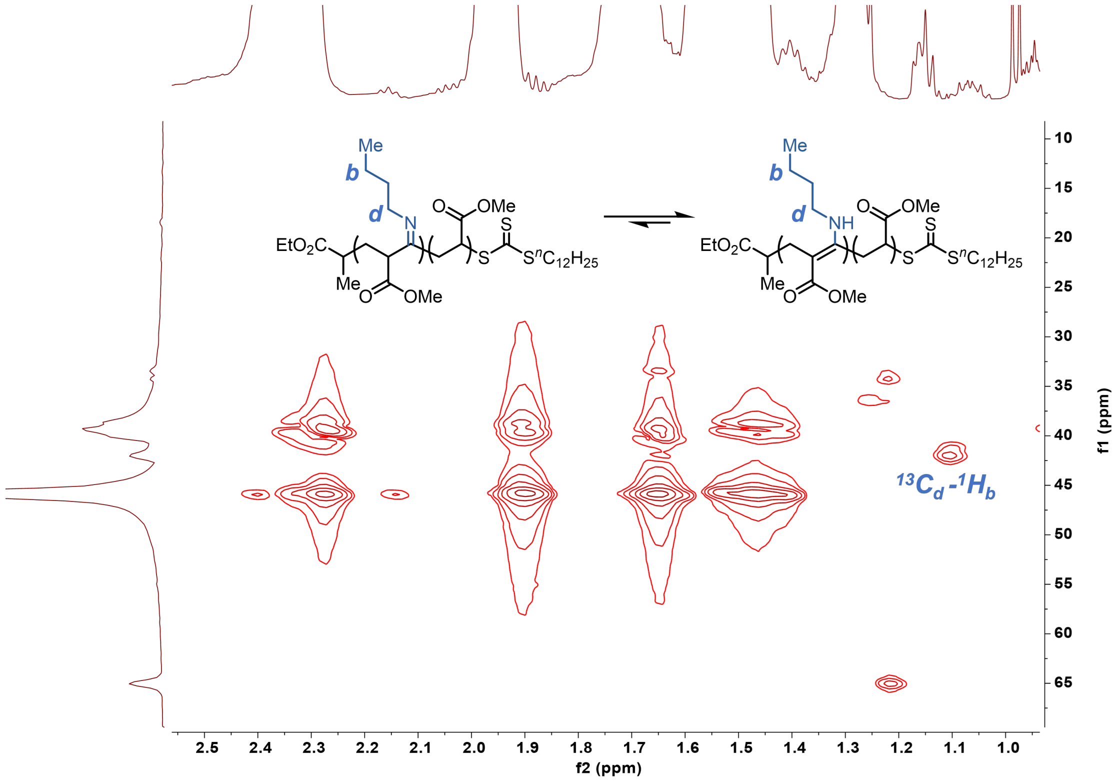

Figure S35. ^{1}H-^{13}C HMBC of PMA-3.3%BI.

The calculation of isocyanide incorporation was based on IR absorption intensity. Each IR spectrum was first normalized to the carbonyl (C=O) absorption of the CO_2Me group (a), and the relative intensity of the C=C bond (a) was used for isocyanide incorporation quantification.

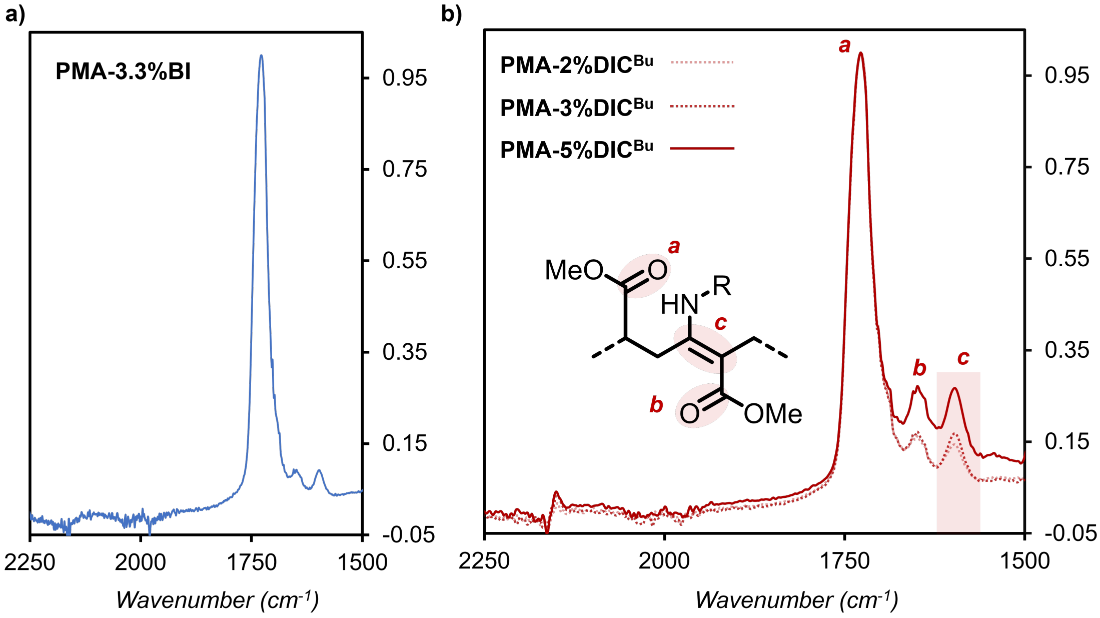

Figure S36. Zoomed IR spectra. a) Normalized IR absorption spectrum of PMA-3.3%BI; b) Normalized IR absorption spectra of PMA-DIC^{Bu}.

Table S3. Incorporation calculation.

Sample	Intensity of peak c	Incorporation
PMA-3.3%BI	10.05%	3.3%
PMA-2%DIC^{Bu}	12.57%	4.1%
PMA-3%DIC^{Bu}	16.86%	5.3%
PMA-5%DIC^{Bu}	26.74%	8.8%

The isocyanide consumption was quantified based on the IR absorption intensity. Pure PMA sample was synthesized according to general procedure D without the addition of DIC^{Bu}. PMA and DIC^{Bu} were then dissolved in dichloromethane (DCM) at different molar ratios, and each solution was deposited onto the IR crystal by solvent casting for individual spectral measurements. The spectra were normalized to the carbonyl stretching vibration of PMA (peak b), and the isocyanide absorption intensity was recorded for comparison. A calibration curve correlating the IR absorption intensity with the molar ratio can be obtained (Figure Sx).

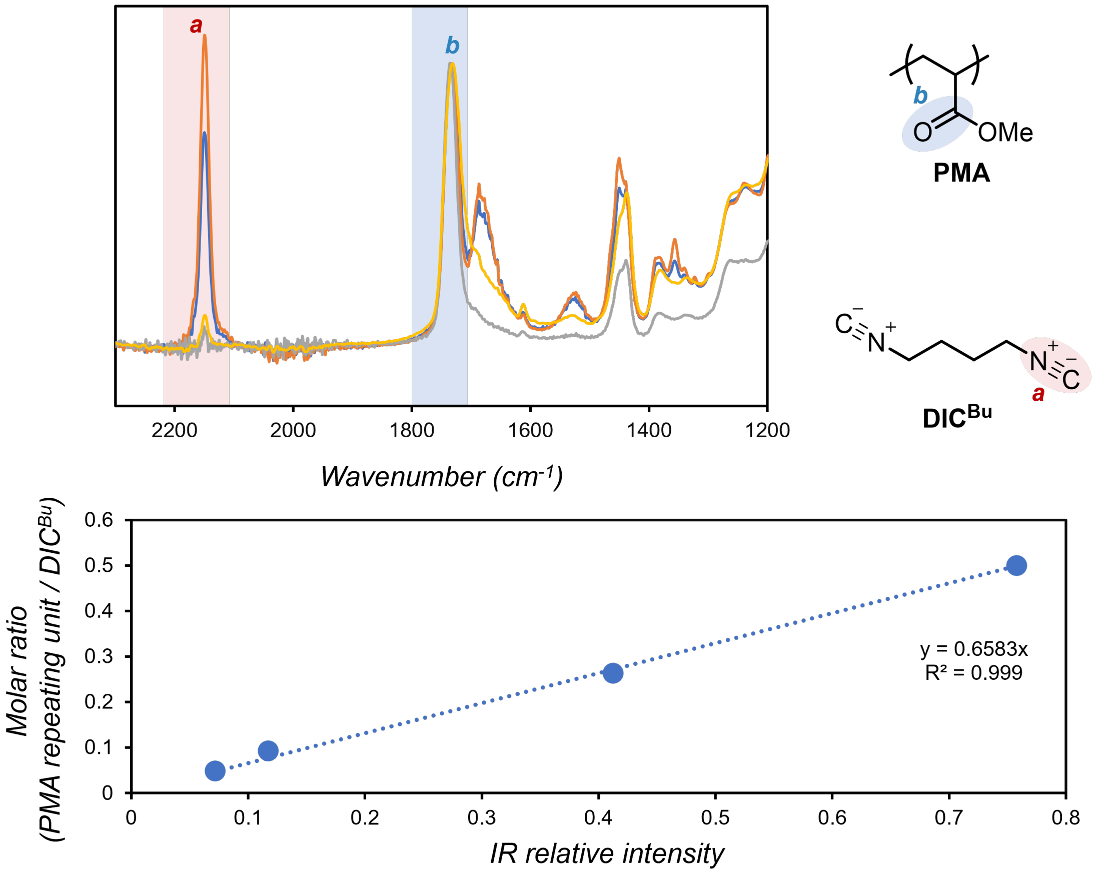

Figure S37. Stacked IR spectra and IR intensity calibration curve.

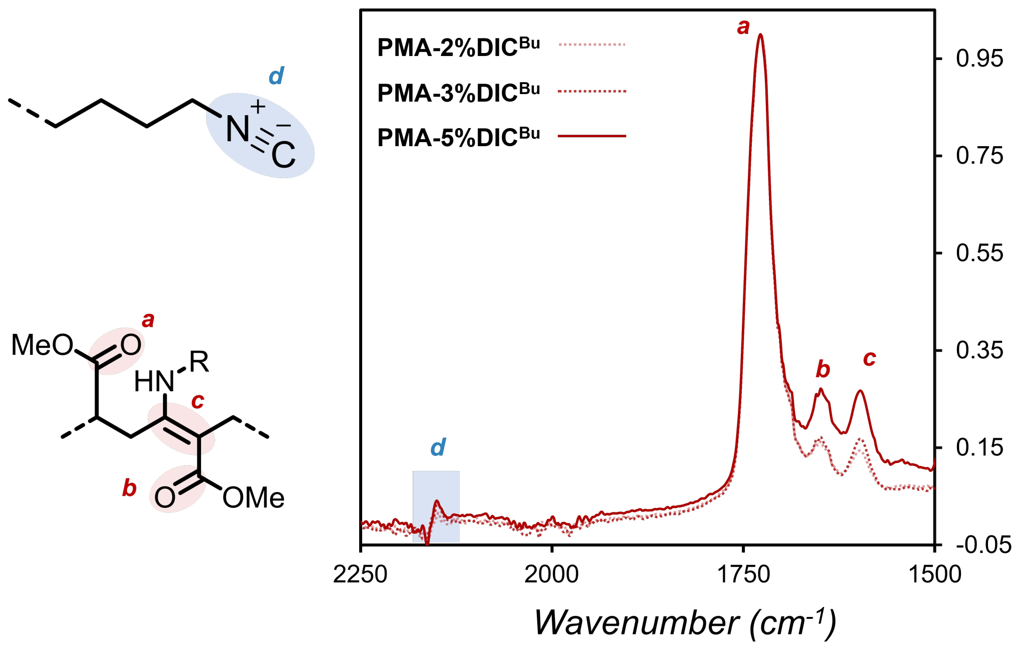

Sample	Intensity of peak d	-NC residual	-NC consumption
PMA-3%DIC^{Bu}	3.01%	1.9%	1-(1.9/6) = 68%
PMA-5%DIC^{Bu}	3.48%	2.3%	1-(2.3/10) = 77%

Figure S38. Isocyanide consumption calculation.

The isocyanide consumption estimation closely agrees with the incorporation calculated from the enamine signal, supporting the validity of the incorporation analysis. However, because the residual isocyanide concentration is very low and the isocyanide peaks partially overlap with diamond/ATR noise, the accuracy of this calculation is limited. Therefore, the incorporation was ultimately determined based on the enamine signal, which provides a more reliable quantitative reference.

Solid state ^{1}H NMR

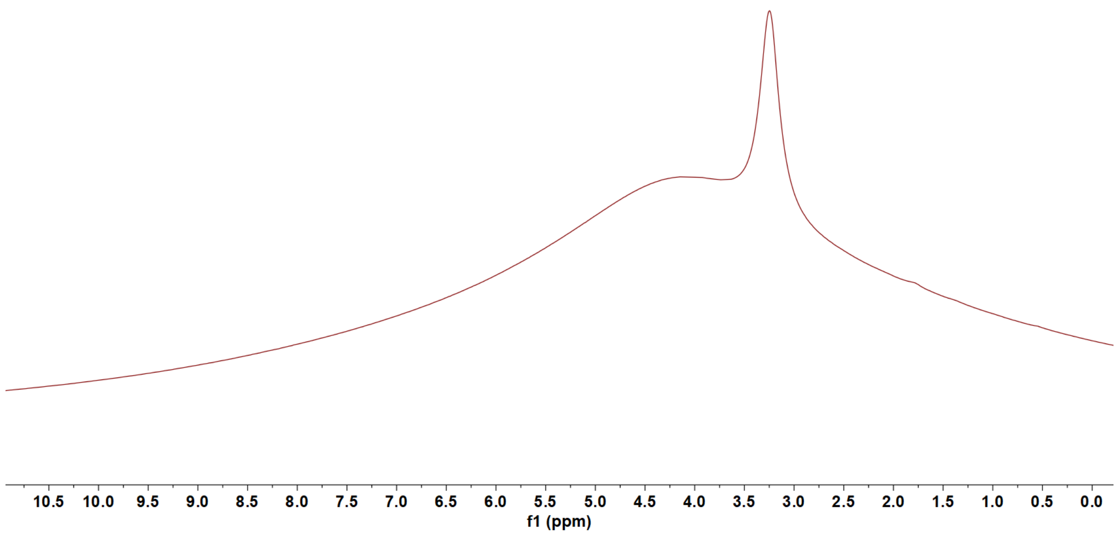

Figure S39. Solid-state ^{1}H NMR of PMA-3%DIC^{Bu}-0.15%BD.

Unfortunately, we were unable to obtain any microstructure information from the solid-state NMR spectrum. This is because solid-state NMR is generally not suitable for cross-linked polymer networks, where the extremely restricted chain mobility causes severe line broadening and results in featureless spectra with poor resolution.

Synthesis of linear PMA and PMA-2%diacrylate

The preparation of PMA and PMA-2%diacrylate thin-film samples was adapted from **General Procedure D**, using the same MA scale, photo initiator loading, set-up, and irradiation time. ^{1}H NMR spectrum of the resulting PMA sample showed no discernible defects. Comparative IR spectroscopy indicated that PMA-2%diacrylate exhibited no spectral differences relative to PMA, whereas PMA-2%DIC^{Bu} displayed an additional set of signals attributed to vinylogous urethane microstructures. These findings collectively suggest that these samples possess well-defined and regular PMA segments.

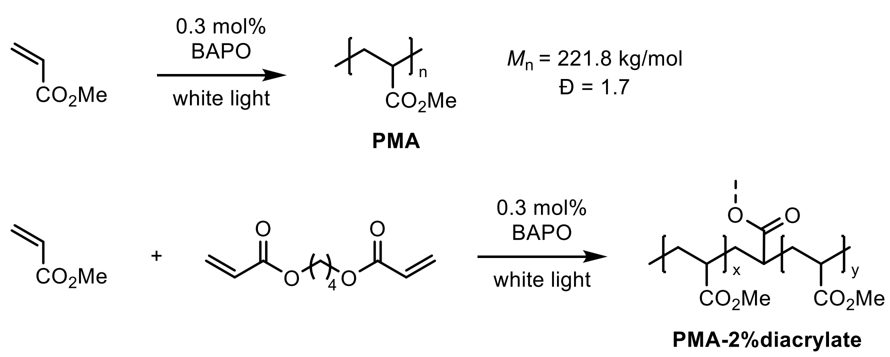

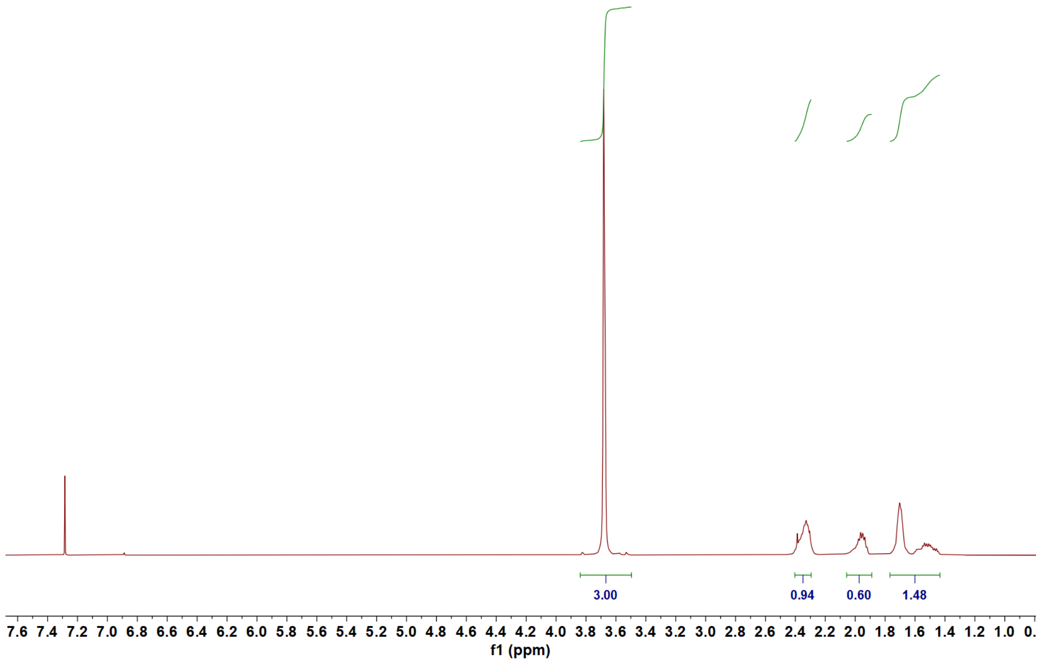

Figure S40. ^{1}H NMR of PMA prepared under photo-initiation with BAPO.

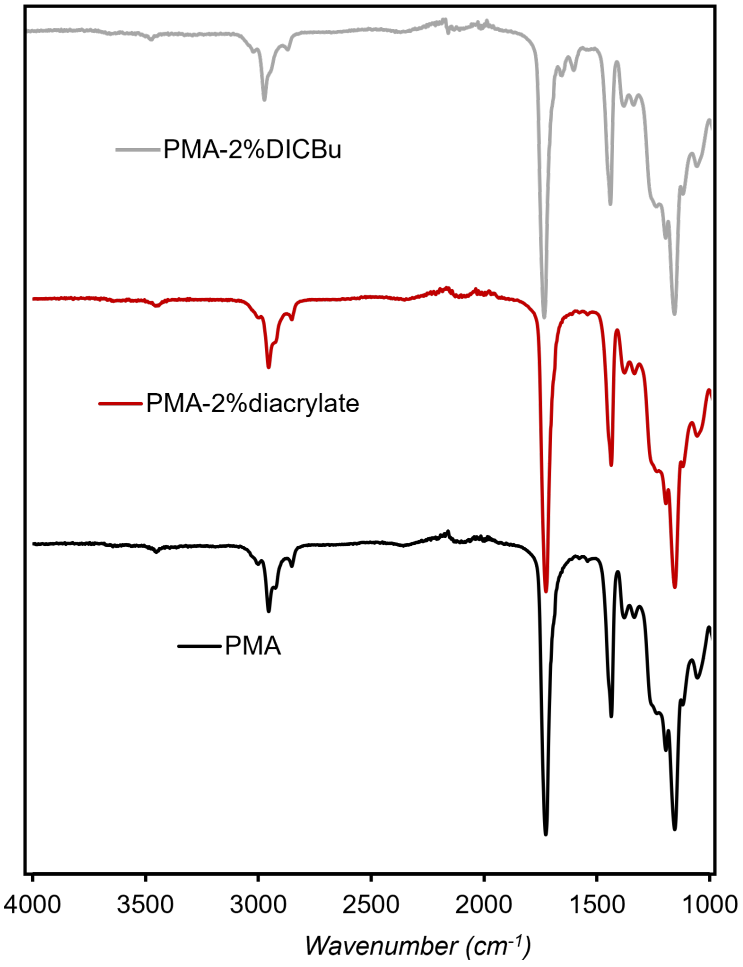

Figure S41. Stacked IR spectra of PMA-2%DIC^{Bu}, PMA-2%diacrylate and PMA.

Synthesis of PMA-DIC^{Bu}-BD

The preparation of PMA-DIC^{Bu}-BD thin-film samples was adapted from **General Procedure D**, using the same MA scale, photo initiator loading, set-up, and irradiation time. IR spectra showed no significant differences compared to the BD-free samples with the same DIC^{Bu} feed loading. The primary chain lengths were also found to be comparable.

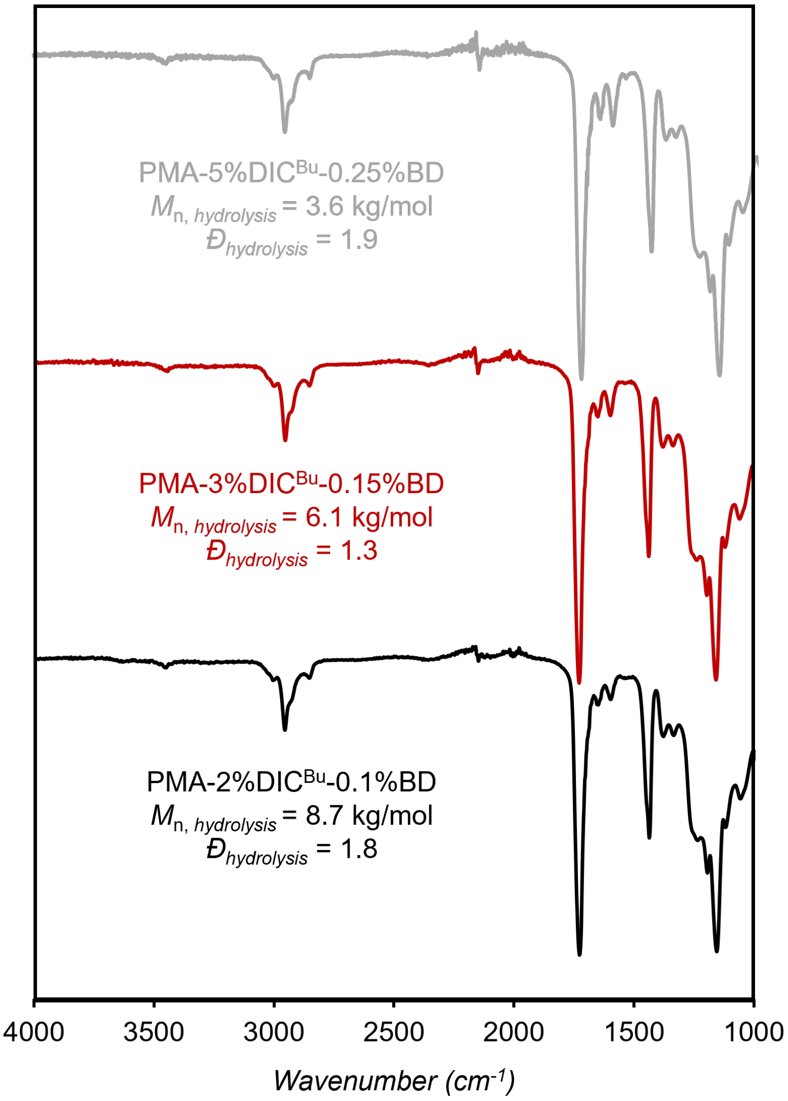

Figure S42. Stacked IR spectra of PMA-DIC^{Bu}-BD samples.

IV. Thermal, viscoelastic, and tensile properties

TGA analysis

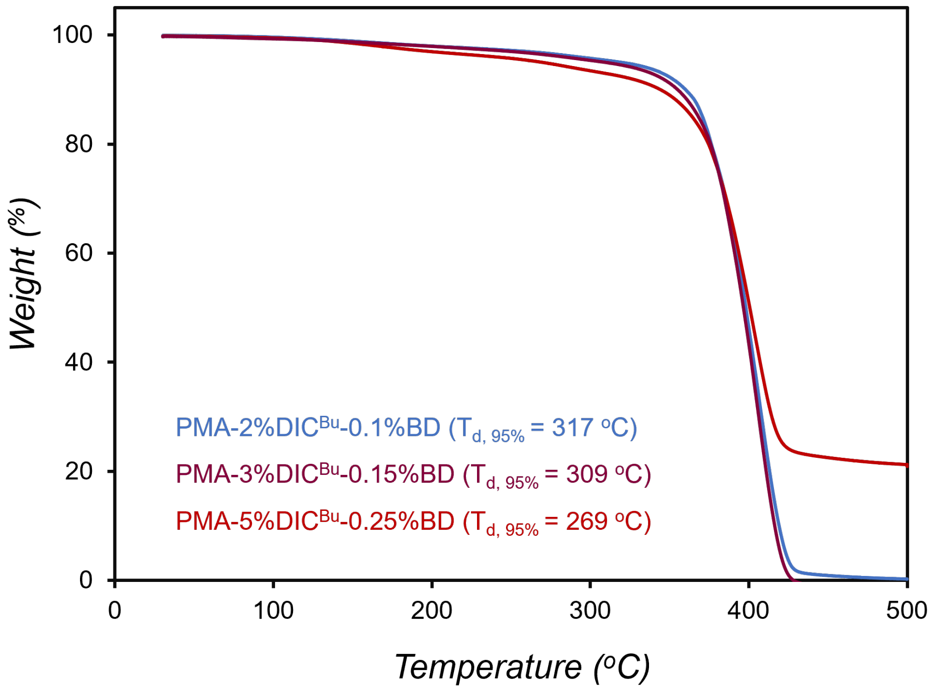

Figure S43. TGA curves.

Evaluation of Plasticizer Effect

200.0 mg vitrimer sample was weighed into a vial, soaked in 5 mL of THF, and stirred using a vortex mixer for 24 hours. The liquid phase was collected, the solvent was evaporated, and the residue was redissolved in 20 mL of DCM, which 2.0 mL solution was taken for GC-FID analysis. A more pronounced free DIC^{Bu} signal was detected in the GC-FID from PMA-2%DIC^{Bu}-0.1%BD to PMA-5%DIC^{Bu}-0.25%BD, suggesting that higher DIC^{Bu} feed loadings result in greater amounts of unincorporated DIC^{Bu}, thereby result in more significant plasticizing effect. TGA and DMA analysis of the samples showed similar trend in T_g variation with BDS, further supporting the plasticizing effect of DIC^{Bu}.

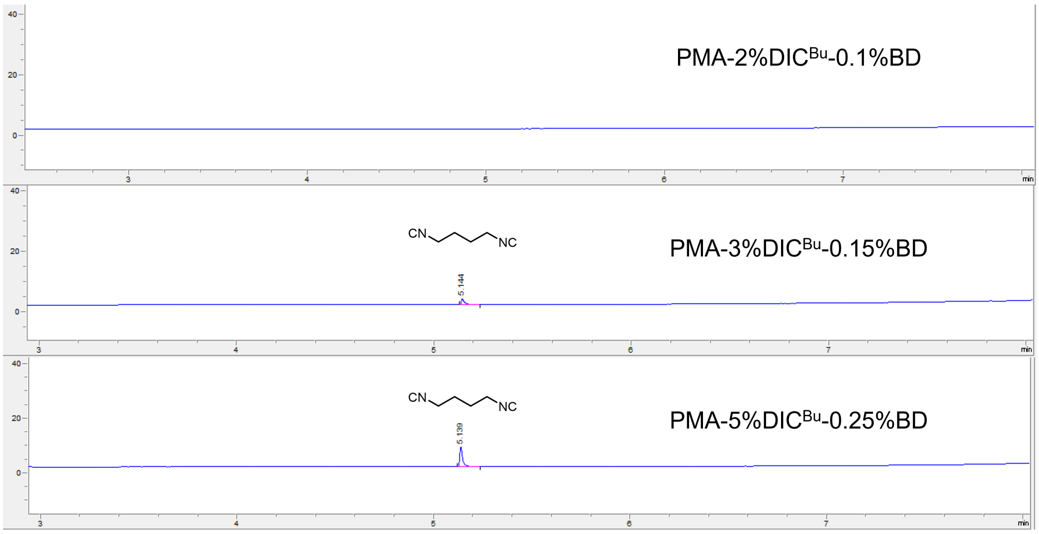

Figure S44. GC-FID signal of residual DIC^{Bu} in different vitrimer samples.

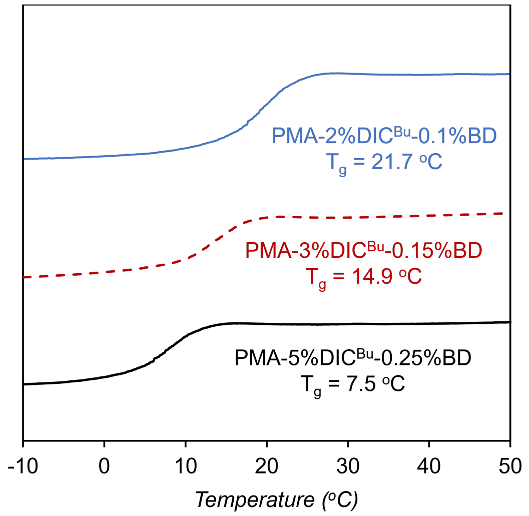

Figure S45. DSC curves of PMA-DIC^{Bu}-BD samples.

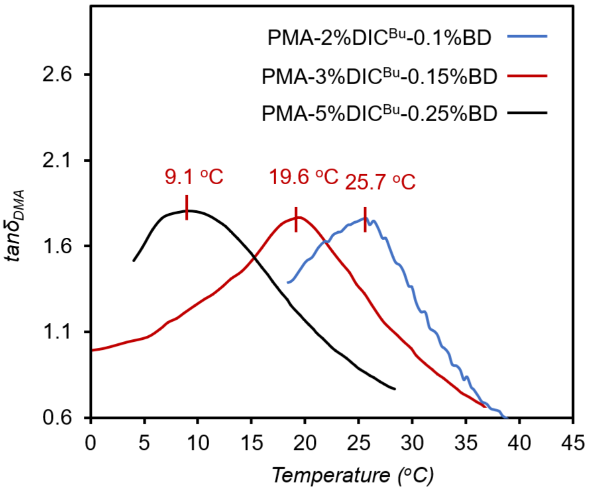

Figure S46. $\tan\delta_{DMA}$ curves of PMA-DIC^{Bu}-BD samples.

Supplementary DMA analysis

DMA for molecular weight between crosslinks (M_x) calculation

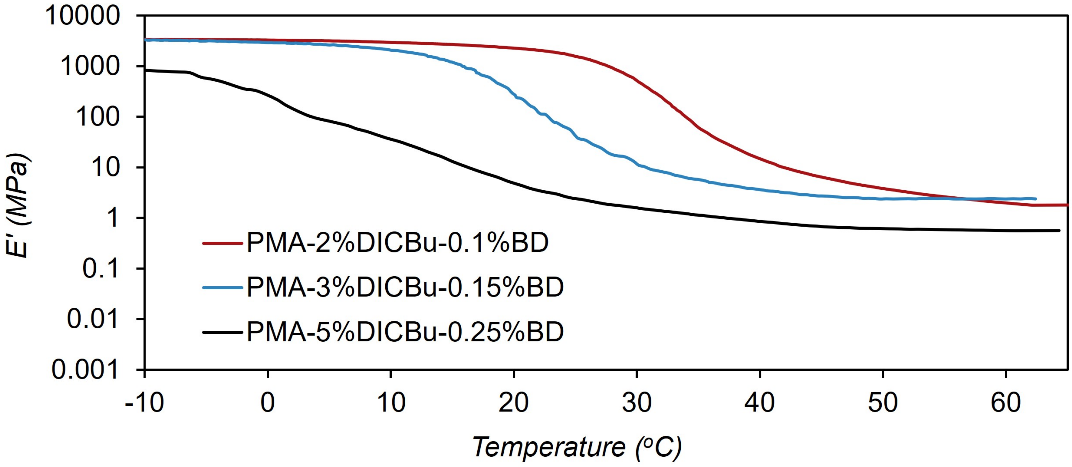

Figure S47. E′ curves of PMA-DIC^{Bu}-BD samples with different DIC^{Bu} loadings.

The calculation of M_x follows the rubber elasticity equations, using the rubbery plateau modulus obtained at 62 °C, which is more than 30 °C above T_g. (ρ ~ 1.20 g/cm^3)

$$v_e = \frac{E'}{3RT}$$

$$M_x = \frac{\rho}{v_e}$$

Table S4. M_x of vitrimers.

Sample	E′ (MPa)	v_e (mol/m³)	M_x (g/mol)
PMA-2%DIC^{Bu}-0.1%BD	1.79	214.3	5600
PMA-3%DIC^{Bu}-0.15%BD	2.36	282.4	4250
PMA-5%DIC^{Bu}-0.25%BD	0.55	65.9	18200

For samples PMA-2%DIC^{Bu}-0.1%BD and PMA-3%DIC^{Bu}-0.15%BD, the M_x values obtained from rubber-elasticity calculations are comparable to the statistical molecular weights between crosslinks as estimated by the incorporation. However, PMA-5%DIC^{Bu}-0.25%BD shows a deviation from this correlation. This deviation is due to a reduction in the primary chain length and the relatively broad molecular-weight distribution (Table 1, entry 5, M_n ≈ 4.3 kDa, Đ ≈ 2.0). Statistically, a significant fraction of chains therefore do not contain a sufficient number of crosslinking points. Even if enamine motifs are incorporated into these chains, they do not contribute effectively to the formation of the elastic network. As a result, the network departs from the ideal affine network assumptions and leads to a decrease in crosslink density. This incomplete network formation is the rationale for excluding PMA-5%DIC^{Bu}-0.25%BD from BDS, DMA, and rheological characterization.

Stress relaxation analysis

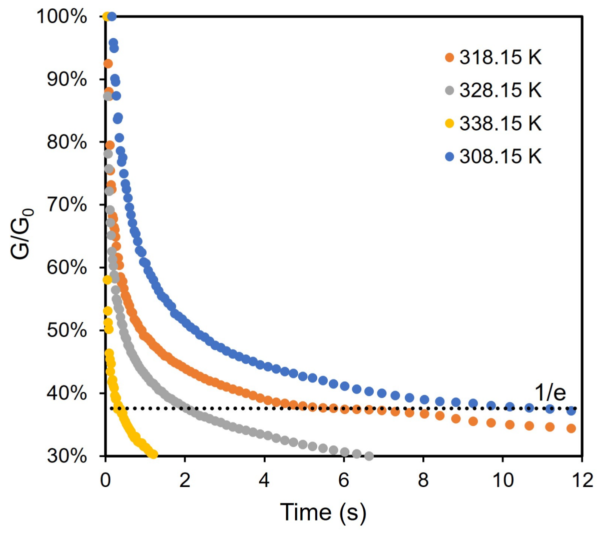

Figure S48. Stress relaxation to G/G_0 =1/e (~37%, dotted line).

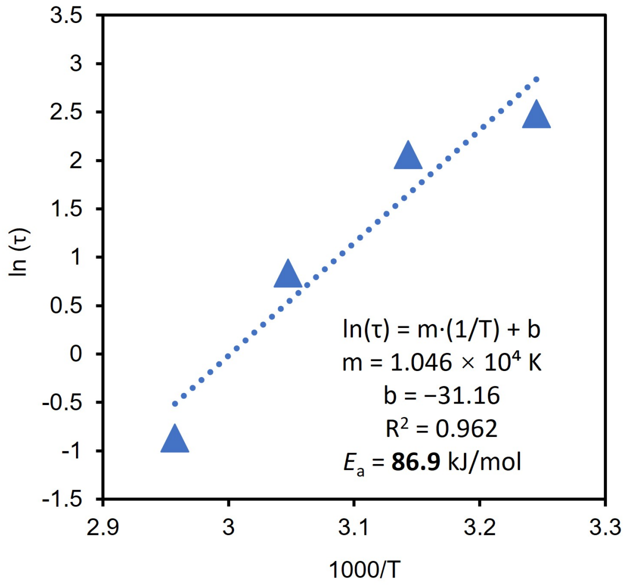

Figure S49. Arrhenius plot with fittings.

The topology freezing transition temperature (T_v) was determined using the Maxwell relation ($\tau = \eta/G$), where η is the viscosity and G is the shear modulus.[5] The temperature at which the viscosity reaches 10^{12} Pa·s was taken as T_v.

$$\tau(T_v) = \frac{\eta(T_v)}{G'}$$

Where G' equals to E'/3, where E' = 2G'(1+μ) with μ being to Poisson's ratio determined equal to ~0.5 for PMA.[6] The E' was obtained from Figure S4x determined by DMA for sample PMA-3%DIC^{Bu}-0.15%BD corresponds to 2.36 MPa which yields a τ = 1.27×10^6 s. Using the Arrhenius fitting, we can solve for $T_v \approx$ 231 K ≈ - 42 °C.

However, based on the above analysis and Figure 3c, we observe that in PMA-3%DIC^{Bu}-0.15%BD, both T_v and the calorimetric T_g are below room temperature. However, under ambient conditions, the material cannot be reprocessed and exhibits almost no observable creep. The fact that theoretical T_v defined as the temperature at which the viscosity reaches 10^{12} Pa·s based on stress-relaxation analysis, was originally proposed for epoxy–acid vitrimers developed by Leibler group.[5] This value was borrowed from glass science, where a viscosity of conventionally defines the glass-transition point, and thus it is an empirical rather than universal criterion. Indeed, several studies have shown that this definition may not accurately reflect the processing behaviour of different vitrimer systems. For example, the Kalow group found that in a Meldrum's acid derived silicone dynamic network, T_v (−6 °C) estimated from stress-relaxation analysis is significantly lower than the processing temperature (150 °C).[7] They attributed this discrepancy to the large spatial separation between reactive partners, which hinders bond-exchange reactions and renders the network kinetically stabilized. Moreover, other studies have noted that commonly used methods to determine T_v (such as stress relaxation) are typically performed under applied stress or deformation, which can also lead to inconsistencies between measured T_v and the true onset of flow.[8,9]

Considering the above, we believe that BDS is a more suitable technique to investigate the intrinsic T_v of our material, since no deformation is applied during dielectric measurements. Across the same lines, the Ribes-Greus group determined the onset temperature for the bond exchange relaxation as the temperature at which they observe the bond exchange relaxation peak at the lowest measured frequency via BDS.[10] They support the idea that the T_v acts as the onset temperature where the timescales of the process become significant, originally proposed by Hubbard *et al.*[11]

Tensile test results

To validate the role of BD, control experiments were performed. The tensile properties of BD-free PMA-3%DIC samples were first measured and found to be comparable to those of PMA-3%DIC-0.15BD. The BD-free samples were then shredded, coated with 0.15 mol% BD uniformly across the fragment surfaces, and subjected to compression molding. After the first reprocessing, the samples exhibited inferior tensile properties, likely due to the inhomogeneous distribution of BD and the resulting defects. However, after a second reprocessing cycle, the tensile properties became comparable to those of the original samples. In contrast, shredded PMA-3%DIC samples without the addition of BD could not be effectively remolded, further supporting the role of BD as a bond exchange activator.

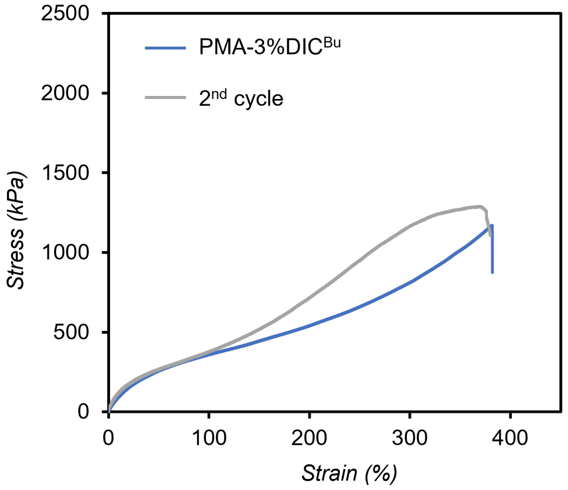

Figure S50. Tensile test result of reprocessed samples with BD.

Table S5. Mechanical properties of samples after compression molding. (Young's modulus was calculated from the slope within 5% strain)

Cycle	Max stress (KPa)	Strain at break (%)	Young's modulus (MPa)
PMA-3%DIC^{Bu}-0.15%BD	1038±134	386±14	1.03±0.12
1	1156±141	374±23	1.46±0.42
2	1071±84	351±20	1.12±0.38
3	995±42	324±10	1.47±0.11
PMA-3%DIC^{Bu}	1098±190	387±4	1.01±0.12
2	1235±56	377±33	1.36±0.18

After reprocessing at 120 °C, the enamine linkages likely underwent dissociation, as evidenced by the disappearance of the characteristic enamine peaks and the appearance of new peaks resembling those of polyketones in the IR spectra (Figure S4x). This dissociation is likely due to hydrolysis of the enamine moieties by trace moisture from ambient air during the melt-processing step. The reprocessed material exhibited a pronounced reduction in mechanical strength while becoming highly extensible (fracture strain ≈ 1800%, Figure S4x), collectively suggesting the formation of a predominantly linear polymer structure.

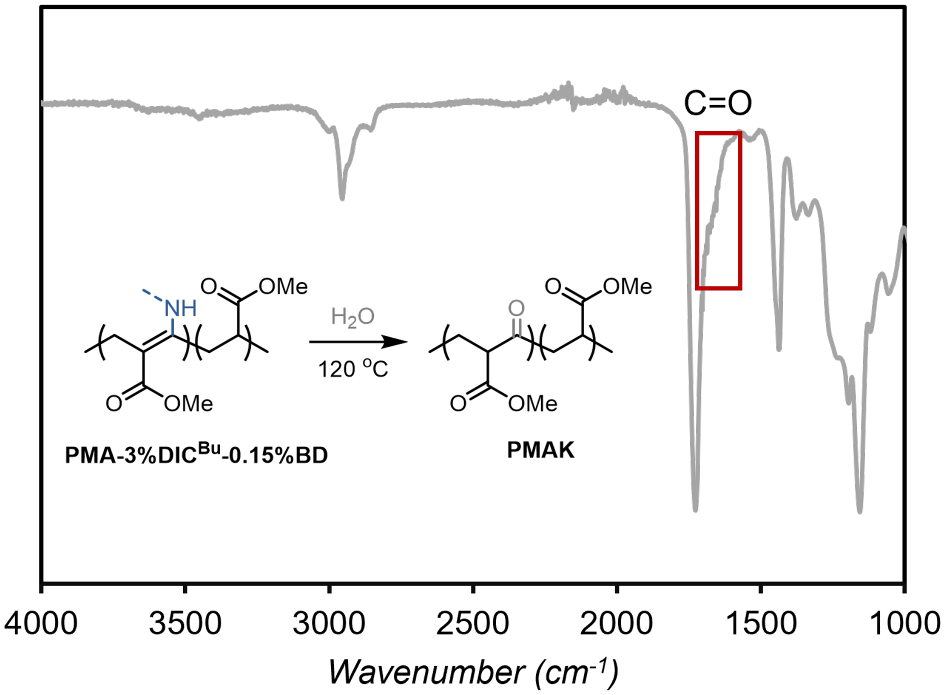

Figure S51. IR spectrum of PMA-3%DIC^{Bu}-0.15%BD reprocessed at 120 ^{o}C.

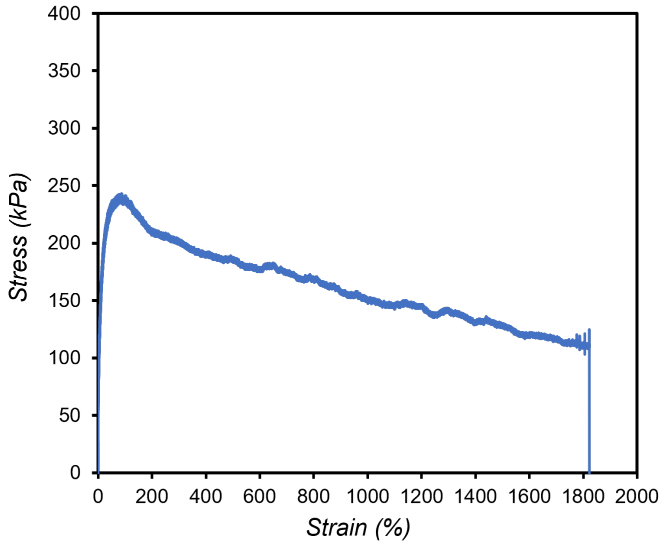

Figure S52. Tensile test result after reprocessing at 120 ^{o}C.

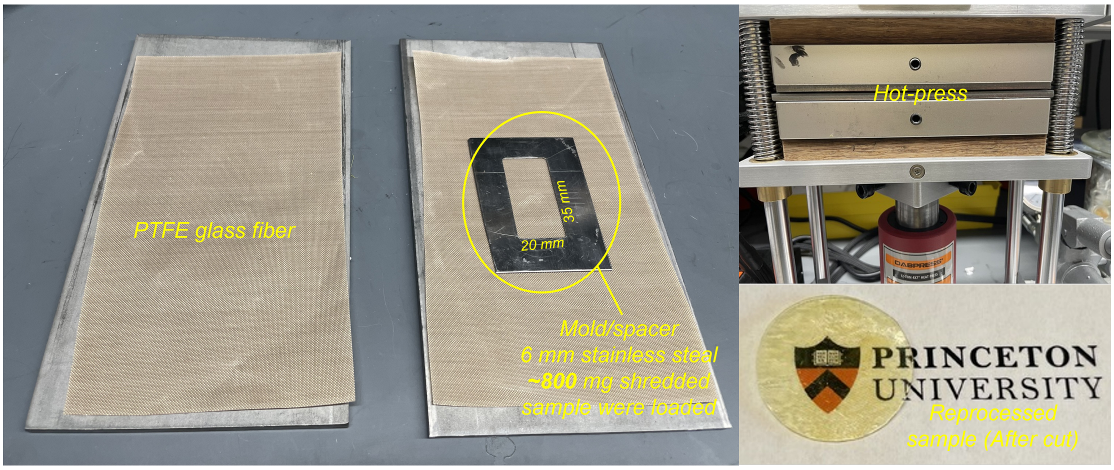

Figure S53. Compression molding set-up.

V. Supplementary BDS data

The experimentally obtained isothermal dielectric spectra were described by the Havriliak-Negami function[12] in its generalized complex permittivity form for *N* relaxation processes and conductivity contribution:

$$\varepsilon_{HN}^{*}(\omega) = \varepsilon_{\infty} + \sum_{i=1}^{N}\left[\frac{\Delta\varepsilon}{[1+(i\omega\tau_{HN})^{m}]^{n}}\right] + \frac{\sigma_0}{i\varepsilon_0\omega^{s}} \qquad (S1)$$

$$\tau = \tau_{HN}\left[\frac{sin\left(\frac{mn\pi}{2+2n}\right)}{sin\left(\frac{m\pi}{2+2n}\right)}\right]^{\frac{1}{m}} \qquad (S2)$$

where ω is the angular frequency, τ_{HN} corresponds to the HN characteristic relaxation time. $\Delta\varepsilon$ is the dielectric relaxation strength where $\Delta\varepsilon = \varepsilon_s - \varepsilon_{\infty}$ with ε_s and ε_{∞} being the static and infinite frequency permittivity values respectively ($\varepsilon_s = \lim_{\omega\to 0}\varepsilon'$ and $\varepsilon_{\infty} = \lim_{\omega\to\infty}\varepsilon'$) and ε_0 is the free space permittivity (8.854x10^{-12} F/m). Shape parameters *m* and *n* correspond to the symmetrical and asymmetrical distribution of relaxation times respectively and vary between $0 < m, m{*}n \leq 1$; for $m = n = 1$ the eq. S1 reduces to the Debye model for a single relaxation time[12]. When $n \neq 1$ the HN characteristic relaxation time of the dielectric process is not equal to the relaxation time τ reciprocal value of the angular frequency where the loss peak is observed, i.e., $\tau_{HN} \neq (\omega_{max})^{-1}$, as shown by eq. S2.[13] Examples of frequency-dependent HN fittings at 30 °C are presented in Figure S54.

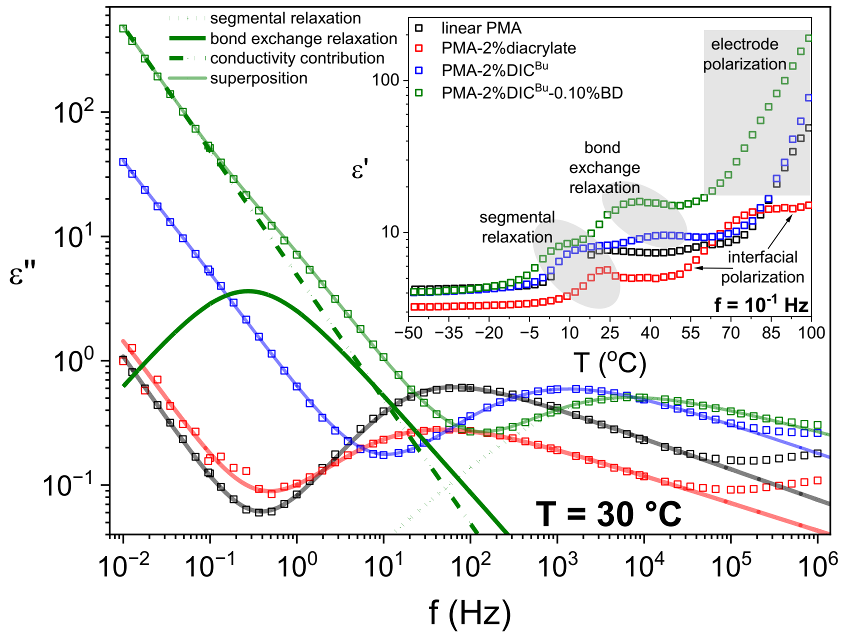

Figure S54. The imaginary part of dielectric permittivity as a function of frequency at 30 °C. The open symbols correspond to the experimental data whilst the solid lines are superpositions of the Havriliak-Negami fittings; the deconvoluted curves are shown for sample **PMA-2%DIC^{Bu}-0.10%BD**. The inset shows the real part of dielectric permittivity as a function of temperature at 10^{-1} Hz.

Table S6. Havriliak-Negami shape parameters. The values are averages over the temperature range and the errors correspond to the standard deviation. The large error values in the dielectric strength of the bond exchange relaxation are attributed to the gradual incorporation of the free BD.

Sample	Dielectric relaxation	Symmetrical shape parameter (Cole-Cole) *m*	Asymmetrical shape parameter (Davidson-Cole) *n*	*Δε*
linear PMA	*β*-relaxation	0.336±0.02	0.58	1.16
	segmental relaxation	0.844±0.009	0.226±0.04	3.224±0.2
PMA-2%diacrylate	*β*-relaxation	0.585±0.004	0.392±0.02	0.622±0.001
	segmental relaxation	0.701±0.03	0.280±0.04	1.473±0.1
	Interfacial polarization	0.957±0.01	1	8.438±0.8
PMA-2%DIC^{Bu}	*β*-relaxation	0.386±0.02	0.439±0.02	1.120±0.002
	segmental relaxation	0.619±0.06	0.362±0.03	3.256±0.1
	bond exchange relaxation	0.898±0.08	0.765±0.07	3.145±1
PMA-2%DIC^{Bu}-	*β*-relaxation	0.387±0.02	0.471±0.007	1.035±0.01
	segmental relaxation	0.550±0.06	0.462±0.1	2.897±0.8

0.10%BD	bond exchange relaxation	0.974±0.06	0.854±0.04	5.098±2
PMA-3%DIC^{Bu}-0.15%BD	β-relaxation	0.402±0.03	0.456±0.006	1.392±0.001
	segmental relaxation	0.568±0.03	0.442±0.06	4.098±0.5
	bond exchange relaxation	0.889±0.08	0.720±0.2	5.958±4
PMA-3%DIC^{Bu}-0.15%BD annealed	β-relaxation	0.394±0.02	0.447±0.01	0.630
	segmental relaxation	0.576±0.03	0.433±0.02	1.679±0.04
	bond exchange relaxation	0.845±0.02	1	3.716±0.3
PMA-5%DIC^{Bu}-0.25%BD	β-relaxation	0.402±0.03	0.407±0.006	1.24
	segmental relaxation	0.622±0.06	0.219±0.04	4.099±0.5
	bond exchange relaxation	0.803±0.08	0.750±0.02	9.218±10

To calculate the derivative permittivity, a simple mathematical treatment on the real part of dielectric permittivity is required, as seen below:

$$\varepsilon''_{der} = -\frac{\pi}{2}\frac{\partial \varepsilon'(\omega)}{\partial ln\omega} \approx \varepsilon''_{dipolar} \quad (S3)$$

As expected from a secondary dielectric relaxation, the temperature dependence of the β-relaxation follows the Arrhenius law, as seen in eq. S4.

$$\tau = \tau_0 e^{\frac{E_a}{k_B T}} \quad (S4)$$

Where τ_0 is a pre-exponential factor corresponding to the relaxation time value at infinite temperature, E_a and k_B are the activation energy and the Boltzmann constant, respectively, and T the absolute temperature.

The segmental dynamics exhibit the expected non-Arrhenius temperature dependence that is commonly described by the Vogel-Fulcher-Tammann equation in eq. S5.

$$\tau = \tau_0 e^{\frac{DT_0}{T-T_0}} \quad (S5)$$

Where τ_0 is a pre-exponential factor corresponding to the relaxation time value at infinite temperature and D is a dimensionless parameter that is associated with the fragility of the segmental relaxation and describes the deviation from the Arrhenius behavior. T_0 is the Vogel temperature and corresponds to the extrapolated temperature at which the relaxation time of the segmental process reaches infinity, i.e., when the segmental mobility completely freezes, and is about 30 – 70 °C lower than the calorimetric T_g.[14–16] Finally, T is the absolute temperature.

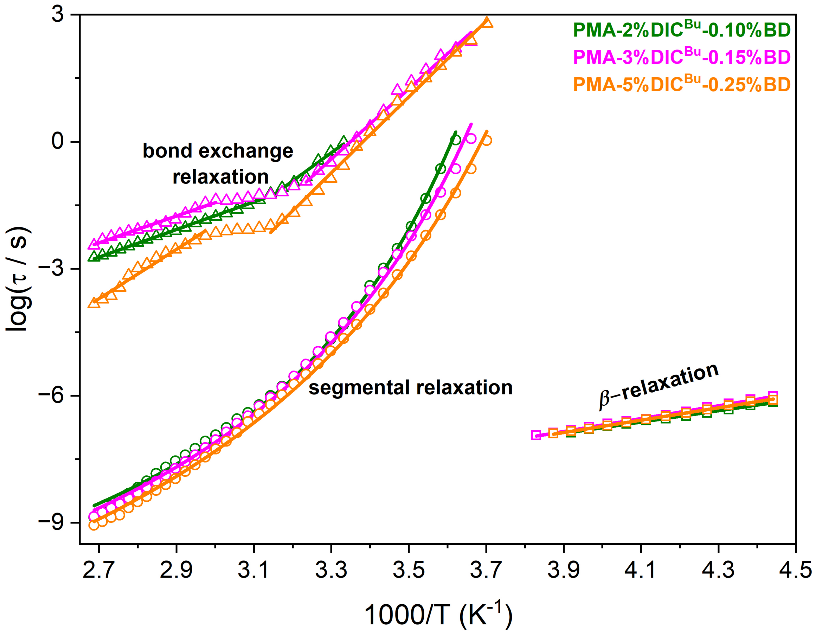

Figure S56. Relaxation map of BD-containing vitrimer samples at different cross-linking densities.

Similar to the temperature dependence of the bond exchange relaxation for samples **PMA-2%DIC^{Bu}** and **PMA-2%DIC^{Bu}-0.10%BD** (Figure 4d), we also observe a similar behavior in vitrimers with varying cross-linking densities (Figure S56). Considering that the measurements are performed on fresh-made samples, we regard the measurements as an *in situ* annealing process, resulting in the vitrimer network re-formation triggered by higher BD incorporation. The Arrhenius and VFT fitting parameters are provided below in Table S6.

Between samples **PMA-2%DIC^{Bu}-0.10%BD** and **PMA-3%DIC^{Bu}-0.15%BD**, we observe similar E_a indicating that the change in cross-linking density is not significantly affecting the bond exchange rate. However, **PMA-5%DIC^{Bu}-0.25%BD**, exhibits a much higher E_a, indicating that the increased cross-linking density causes a restriction in bond exchange, which can be further corroborated by the segmental relaxation dynamics (Figure S50). Although the T_0 and T_g values are decreasing, indicating generally faster segmental dynamics, at high temperatures –where the bond exchange relaxation becomes dominant– the relaxation time values of the **PMA-5%DIC^{Bu}-0.25%BD** are higher than the other vitrimer samples. According to Table S8, the vitrimer samples are characterized by low fragility values indicating almost strong behavior, attributed to the presence of cross-links that restrict segmental mobility. However, at higher temperatures we observe a slowing down of the segmental relaxation time with increasing DIC^{Bu}. This observation led us to believe that the high temperature response of our vitrimer systems is dominated by the increased cross-linking density, which is also supported by low fragility values.

Table S7. Arrhenius law and Vogel-Fulcher-Tammann fitting parameters for each dielectric relaxation recorded for all the samples under study.

Sample	Dielectric Relaxation				
	bond exchange relaxation		segmental relaxation		β-relaxation
	High-T	Low-T			
	E_a (kJ/mol)	E_a (kJ/mol)	D	T_0 (°C)	E_a (kJ/mol)
linear PMA	-	-	5.47	-28	27.3
PMA-2%diacrylate	-	-	5.36	-28	31.4
PMA-2%DIC^{Bu}	95.4	93.1	5.80	-38	26.6
PMA-2%DIC^{Bu}-0.10%BD	62.5	127.8	5.62	-41	25.7
PMA-3%DIC^{Bu}-0.15%BD	60.7	158.8	6.86	-49	29.1
PMA-3%DIC^{Bu}-0.15%BD annealed	108.2		8.81	-49	29.2
PMA-5%DIC^{Bu}-0.25%BD	111.5	171.6	8.85	-60	28.0

By employing the T_g values obtained from DSC (Figure S45) and the VFT fitting parameters presented in Table S7 [17] we can calculate the fragility m presented in Table S8 below:

$$m = \frac{DT_0T_g}{\left(T_g - T_0\right)^2 ln(10)} \qquad (S6)$$

Table S8. Fragility values for the samples under study.

Sample	Fragility m
PMA-2%DIC^{Bu}-0.10%BD	42.5
PMA-3%DIC^{Bu}-0.15%BD	49.8
PMA-5%DIC^{Bu}-0.25%BD	50.5

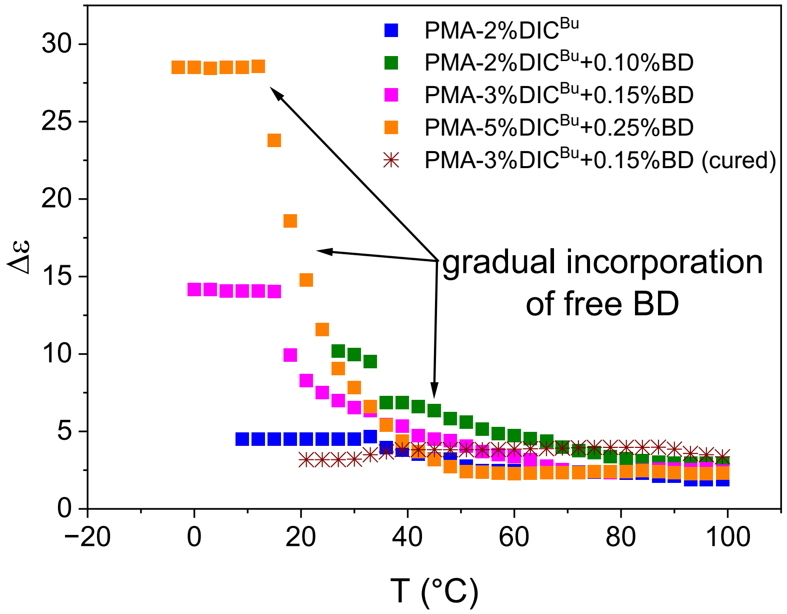

Figure S57. The dielectric strength of the bond exchange relaxation over temperature. Due to the presence of unincorporated BD in the samples, the gradual BD incorporation is apparent in the values that reach a plataeu in the order of Δε = 2 – 4, at high temperatures.

Apart from the dielectric relaxation dynamics of vitrimers, BDS analysis allows also an in-depth study of electrical conductivity that can reveal how vitrimers behave under the application of an electric field.[10] Therefore, to investigate the charge transport of our systems, we utilized the real and imaginary parts of the ac complex conductivity as a function of frequency ($\sigma^*_{ac} = \sigma'_{ac} + i\sigma''_{ac}$) as presented in Figure S58, The σ'_{ac} is defined as follows:

$$\sigma'_{ac} = \varepsilon_0 \omega \varepsilon''(\omega) \qquad (S7)$$

Going from high frequencies to low, the σ'_{ac} values follow the typical behavior with frequency, gradually decreasing until they reach a plateau value at an onset frequency, indicating the beginning of the σ_{dc} region, where the σ'_{ac} values are independent of frequency. With increasing temperature, the onset frequency increases, as well as the σ'_{ac} plateau values (σ_{dc}). As the temperature increases, the σ_{dc} values increase indicating the thermal-assisted nature of charge transport, which we attribute to hopping of charge carriers.[18]

It should be noted that, at the examined temperature range, the σ'_{ac} values do not exhibit a step-like behavior at frequencies lower than the plateau onset frequency, indicating that the contribution of electrode polarization is relatively weak. This can be further supported by the σ''_{ac} frequency dependence, i.e., at 99 °C designated with a red dotted line (Figure S58a), where the ratio between the σ''_{ac} peak value (~ 0.03 Hz) and the σ''_{ac} minimum value (~ 1 Hz) is very low, i.e., ~ 2. Therefore, the behavior we observe here is significantly different compared to systems with strong electrode polarization.[19]

To highlight the fact, we compared the recorded σ'_{ac} values at 0.01 Hz with the σ_0 values we calculated based on eq. (S8) presented later, as depicted in Figure S58b. It is apparent

that both formalisms yield almost identical values, indicating that the dc electrical conductivity contribution is much stronger than any polarization contributions in the σ'_{ac} values at 0.01 Hz. A minor divergence observed at low temperatures and is associated with dipolar phenomena that elevate the σ'_{ac} values compared to σ_0. Finally, in Figure S58c we present a comparison between the conductivity relaxation time, τ_c, calculated as the reciprocal angular frequency at which $\varepsilon' = \varepsilon''$ ($\tan\delta_{BDS} = 1$, also the frequency at which the imaginary part of electric modulus exhibits a peak associated with the conductivity relaxation), and the bond exchange relaxation time, τ. Figure S58c is complementary to the discussion with Figure 5 presented in the main text, indicating a correlation between the timescales of the two phenomena (electrical conductivity and bond exchange relaxation). However, the fact that there is no 1-1 correlation highlights that we have not misidentified the process, which is indeed associated with the bond exchange relaxation, also corroborated by the stress relaxation experiments presented earlier.

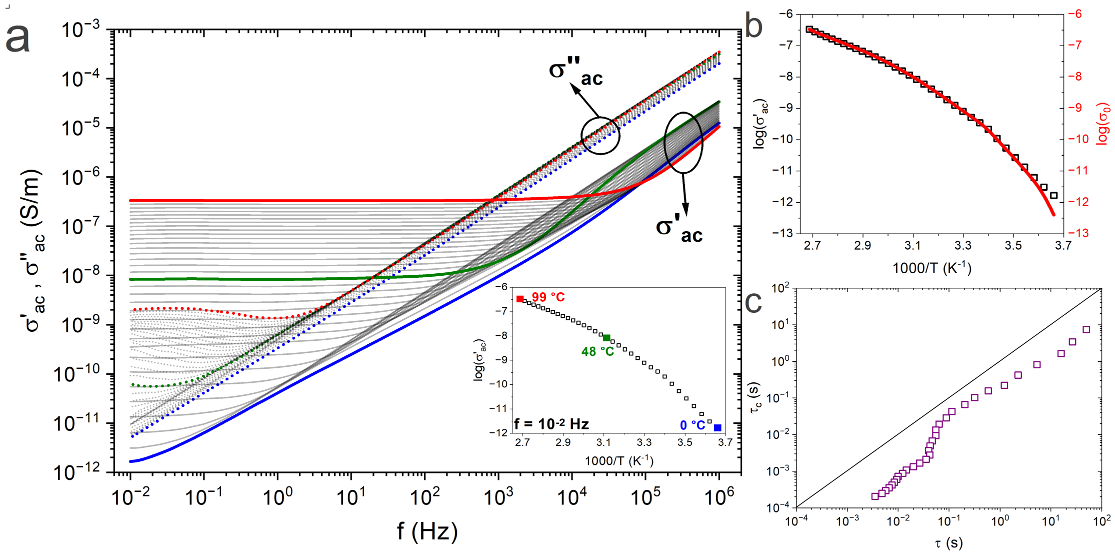

Figure S58. Electrical conductivity analysis of **PMA-3%DICBu-0.15%BD**. (a) The real and imaginary parts of ac conductivity as a function of frequency varying the temperature. As inset, the real part of ac conductivity at 0.01 Hz against reciprocal temperature. Three temperatures are highlighted with blue, green, and red, namely 0 °C, 48 °C, and 99 °C, respectively. (b) The σ'_{ac} values at 0.01 Hz are against the compared against σ_0 values calculated via eq. (S8), both as a function of reciprocal temperature. (c) Scaling of the conductivity relaxation time, τ_c, determined isothermally when $\varepsilon' = \varepsilon''$, with the bond exchange relaxation time, τ. The straight black line corresponds to a 1-1 relationship between the two values.

For s < 1, electrical conductivity is still dependent upon frequency, therefore is it represented by σ_0 which can be calculated from the frequency-dependent response according to eq. S8 that has been introduced and discussed elsewhere:[20,21]

$$\sigma_0 = \varepsilon_0 \omega^s [\varepsilon''(\omega) - \varepsilon''_{der}(\omega)] \qquad (S8)$$

Where σ_0 is the isothermal value of electrical conductivity and $\varepsilon''(\omega)$ and $\varepsilon''_{der}(\omega)$ correspond to the isothermal values of the experimentally obtained imaginary part of dielectric

permittivity, and the isothermal values of the derivative permittivity that was introduced earlier in eq. S3. Parameter s takes values between 0.5 and 1.0 and in this case was observed to be within the range of 0.9 to 1.0 indicating close to Ohmic conduction or long range diffusion. For s = 1, then $\sigma_0 = \sigma_{dc}$. By employing this equation, all the polarization effects (including electrode polarization) are taken into account towards a more accurate estimation of σ_0 values.[22] In order to explore the relationship between bond exchange relaxation and electrical conductivity, we employ the Barton, Nakajima and Namikawa (BNN) relation.[23] The BNN relation follows Equation (S9) as seen below:

$$\sigma = \frac{p\varepsilon_0 \Delta\varepsilon}{\tau} \qquad (S9)$$

where $\Delta\varepsilon$ and τ are the isothermal values for the relaxation dielectric strength and the relaxation time obtained for the bond exchange relaxation, respectively. Therefore, σ here corresponds to the electrical conductivity associated only with the bond exchange relaxation. Parameter p is a constant close to unity and in the present study it is assumed equal to one. The underlying physical explanation of the BNN relation lays its foundations on the random free-energy barrier model or symmetric hopping model[18,24] and has found application in ionically[25] and electronically[26] conductive polymers.

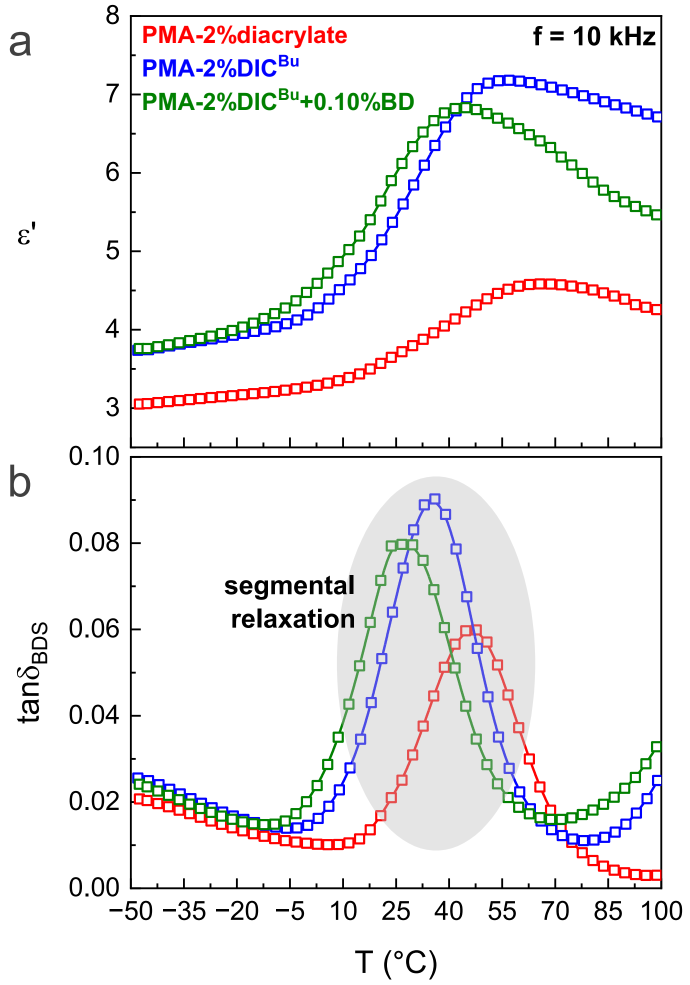

Figure S59. The dielectric performance of **PMA-2%DIC^{Bu}** with and without butyl diamine against non-dynamically crosslinked **PMA-2%diacrylate**: (a) the real part of dielectric

permittivity and (b) the dielectric loss tangent, both as a function of temperature at 10 kHz.

VI. Computational details

Table S9. Computed thermal correction to Gibbs free energies (TCG), single point energies (SPE) for the stationary points

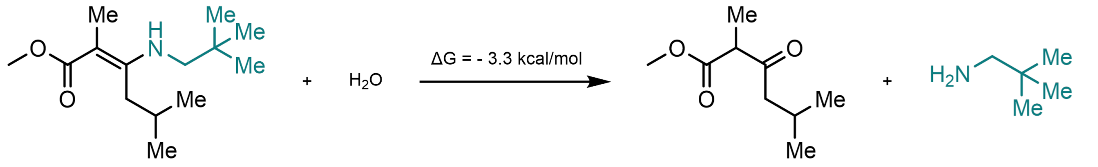

enamine **ketone** **free amine**

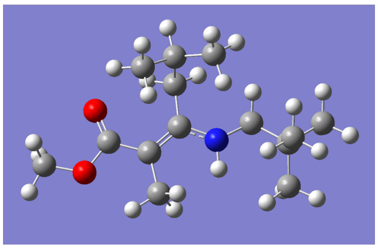

Intermediate	TCG (hartree)	SPE (hartree)
enamine	0.348435	-754.828433
H_2O	0.003262	-76.419718
ketone	0.147315	-577.990465
free amine	0.201472	-578.191937

All computations were performed in Gaussian 16 at the M06-2X/6-311G(d, p) level of theory. All discussed energy differences were based on Gibbs energies at 298 K (standard states are the hypothetical states at 1 mol/L). All calculations utilized the self-consistent reaction field in the conductor-like polarizable continuum model (CPCM) with acetonitrile as the solvent. All energies are given in kcal/mol.

Cartesian Coordinates

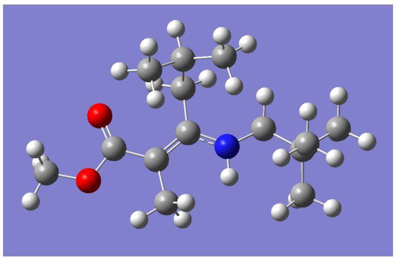

enamine

1	C	-2.806685	-0.435139	-0.201789
2	O	-3.154449	0.616895	-0.712342
3	O	-3.755433	-1.234779	0.354708
4	C	-5.094694	-0.914369	0.431154
5	H	-5.639880	-1.745117	0.924304
6	H	-5.513111	-0.766760	-0.587440

7	H	-5.231970	0.010322	1.031585
8	C	-1.366755	-0.899331	-0.227162
9	C	-0.276474	-0.149966	-0.657142
10	C	-1.148818	-2.327510	0.257506
11	H	-2.070809	-2.831749	0.606949
12	H	-0.437583	-2.331797	1.110519
13	H	-0.761400	-2.961214	-0.567647
14	N	1.018116	-0.796786	-0.734172
15	C	-0.433407	1.298351	-1.165831
16	H	-1.328401	1.342198	-1.809172
17	H	0.330420	1.517682	-1.940153
18	C	-0.452791	2.478518	-0.136265
19	C	-1.266013	2.218947	1.141462
20	H	-0.892521	3.353685	-0.664285
21	C	0.953348	2.935559	0.257699
22	H	-2.333976	2.054034	0.931948
23	H	-1.221012	3.107242	1.807467
24	H	-0.851021	1.350463	1.697480
25	H	1.469259	2.157558	0.833321
26	H	0.899523	3.847169	0.890943
27	H	1.544308	3.197187	-0.646519
28	H	1.068843	-1.818827	-0.688492
29	C	2.303623	-0.144540	-0.925250
30	H	2.197402	0.947733	-0.908722
31	H	2.693642	-0.409606	-1.932233
32	C	3.355243	-0.590072	0.144860
33	C	2.833100	-0.389947	1.584054
34	C	4.632042	0.252775	-0.045006
35	C	3.725729	-2.080377	-0.036040
36	H	4.410316	1.333337	0.094597
37	H	5.049286	0.111804	-1.065267
38	H	5.410592	-0.036240	0.694021
39	H	2.630987	0.671297	1.804535
40	H	3.589698	-0.730190	2.323545
41	H	1.903667	-0.974058	1.755921
42	H	2.861384	-2.751649	0.140314
43	H	4.515179	-2.374958	0.689319
44	H	4.114743	-2.266373	-1.060055

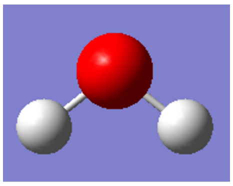

H_2O

O	0.000000	0.118905	0.000000
H	0.754519	-0.475619	0.000000
H	-0.754519	-0.475619	0.000000

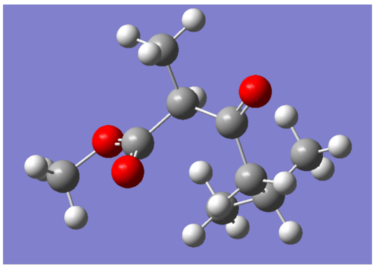

ketone

1	C	-1.295870	0.073066	0.228901
2	O	-1.304149	-0.198255	1.404295
3	O	-2.235546	-0.346371	-0.619407
4	C	-3.272919	-1.137523	-0.063735
5	H	-3.952417	-1.374322	-0.880143
6	H	-3.800193	-0.579637	0.713466
7	H	-2.861379	-2.056591	0.360603
8	C	-0.265849	0.939318	-0.457061
9	C	0.995315	1.133867	0.383094
10	C	-0.903616	2.275104	-0.859908
11	H	-1.765916	2.098206	-1.506330
12	H	-0.177420	2.881941	-1.402942
13	H	-1.229351	2.829981	0.024523
14	C	1.693788	-0.049527	1.024621
15	H	1.104786	-0.372942	1.889746
16	H	2.639125	0.345533	1.410491
17	C	1.978728	-1.276386	0.136547
18	C	0.741087	-2.144331	-0.128039
19	H	2.659807	-1.908615	0.719483
20	C	2.692563	-0.884128	-1.158547
21	H	0.278781	-2.448338	0.817444
22	H	1.032317	-3.051907	-0.667030
23	H	-0.021296	-1.646692	-0.733506
24	H	2.052946	-0.258219	-1.794407

25	H	2.964789	-1.774950	-1.733111
26	H	3.608823	-0.324527	-0.942479
27	H	0.039854	0.435005	-1.382134
28	O	1.445618	2.248291	0.523997

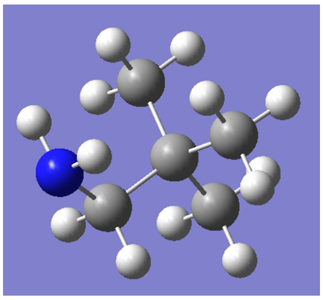

free amine

1	H	0.406634	-2.159533	-0.280660
2	C	0.468443	-1.251935	-0.888871
3	H	1.404971	-1.284445	-1.452362
4	H	-0.350860	-1.267524	-1.612619
5	C	0.415338	0.000000	-0.008792
6	C	-0.870881	0.000024	0.844938
7	C	1.609723	0.000042	0.949338
8	H	1.594604	-0.885668	1.591431
9	H	2.551949	0.000023	0.394567
10	H	1.594598	0.885802	1.591362
11	C	0.468430	1.251870	-0.888965
12	H	1.404957	1.284348	-1.452458
13	H	-0.350874	1.267394	-1.612713
14	H	0.406610	2.159513	-0.280822
15	H	-0.848572	-0.878950	1.498809
16	N	-2.154461	0.000002	0.143038
17	H	-0.848576	0.879038	1.498756
18	H	-2.215259	-0.811348	-0.465212
19	H	-2.215272	0.811330	-0.465239

VII. References

(1) Ricardo, M. G.; Vázquéz-Mena, Y.; Iglesias-Morales, Y.; Wessjohann, L. A.; Rivera, D. G. On the Scope of the Double Ugi Multicomponent Stapling to Produce Helical Peptides. *Bioorganic Chemistry* **2021**, *113*, 104987.

(2) Rivera, D. G.; Wessjohann, L. A. Supramolecular Compounds from Multiple Ugi Multicomponent Macrocyclizations: Peptoid-Based Cryptands, Cages, and Cryptophanes. *J. Am. Chem. Soc.* **2006**, *128* (22), 7122–7123.

(3) Čamdžić, L.; Stache, E. E. Controlled Radical Polymerization of Acrylates and Isocyanides Installs Degradable Functionality into Novel Copolymers. *J. Am. Chem. Soc.* **2023**, *145* (37), 20311–20318.

(4) Čamdžić, L.; Stache, E. E. Photo-Iniferter RAFT Synthesis of Versatile, Nonalternating Poly(Acrylate- *Co* -Isocyanides). *Macromolecules* **2024**, *57* (19), 9250–9256.

(5) Montarnal, D.; Capelot, M.; Tournilhac, F.; Leibler, L. Silica-Like Malleable Materials from Permanent Organic Networks. *Science* **2011**, *334* (6058), 965–968.

(6) Hrouz, J.; Ilavský, M.; Havlíček, I.; Dušek, K. Comparison of the Viscoelastic Penetration and Tensile Behaviour of Poly(Methyl Acrylate) and Poly(Ethyl Acrylate). *Collect. Czech. Chem. Commun.* **1979**, *44* (6), 1942–1948.

(7) Ishibashi, J. S. A.; Kalow, J. A. Vitrimeric Silicone Elastomers Enabled by Dynamic Meldrum's Acid-Derived Cross-Links. *ACS Macro Lett.* **2018**, *7* (4), 482–486.

(8) Kaiser, S.; Novak, P.; Giebler, M.; Gschwandl, M.; Novak, P.; Pilz, G.; Morak, M.; Schlögl, S. The Crucial Role of External Force in the Estimation of the Topology Freezing Transition Temperature of Vitrimers by Elongational Creep Measurements. *Polymer* **2020**, *204*, 122804.

(9) Yang, Y.; Zhang, S.; Zhang, X.; Gao, L.; Wei, Y.; Ji, Y. Detecting Topology Freezing Transition Temperature of Vitrimers by AIE Luminogens. *Nat Commun* **2019**, *10* (1), 3165.

(10) Pascual-Jose, B.; De La Flor, S.; Serra, A.; Ribes-Greus, A. Analysis of Poly(Thiourethane) Covalent Adaptable Network through Broadband Dielectric Spectroscopy. *ACS Appl. Polym. Mater.* **2023**, *5* (2), 1125–1134.

(11) Hubbard, A. M.; Ren, Y.; Konkolewicz, D.; Sarvestani, A.; Picu, C. R.; Kedziora, G. S.; Roy, A.; Varshney, V.; Nepal, D. Vitrimer Transition Temperature Identification: Coupling Various Thermomechanical Methodologies. *ACS Appl. Polym. Mater.* **2021**, *3* (4), 1756–1766.

(12) Havriliak, S.; Negami, S. A Complex Plane Representation of Dielectric and Mechanical Relaxation Processes in Some Polymers. *Polymer* **1967**, *8*, 161–210.

(13) Richert, R.; Stickel, F.; Fee, R. S.; Maroncelli, M. Solvation Dynamics and the Dielectric Response in a Glass-Forming Solvent: From Picoseconds to Seconds. *Chemical Physics Letters* **1994**, *229* (3), 302–308.

(14) Martinez-Garcia, J. C.; Rzoska, S. J.; Drozd-Rzoska, A.; Starzonek, S.; Mauro, J. C. Fragility and Basic Process Energies in Vitrifying Systems. *Sci Rep* **2015**, *5* (1), 8314.

(15) Angell, C. A.; Ngai, K. L.; McKenna, G. B.; McMillan, P. F.; Martin, S. W. Relaxation

in Glassforming Liquids and Amorphous Solids. *Journal of Applied Physics* **2000**, *88* (6), 3113–3157.
(16) Napolitano, S.; Glynos, E.; Tito, N. B. Glass Transition of Polymers in Bulk, Confined Geometries, and near Interfaces. *Rep. Prog. Phys.* **2017**, *80* (3), 036602.
(17) Böhmer, R.; Ngai, K. L.; Angell, C. A.; Plazek, D. J. Nonexponential Relaxations in Strong and Fragile Glass Formers. *The Journal of Chemical Physics* **1993**, *99* (5), 4201–4209.
(18) Dyre, J. C. The Random Free-energy Barrier Model for Ac Conduction in Disordered Solids. *Journal of Applied Physics* **1988**, *64* (5), 2456–2468.
(19) Serghei, A.; Tress, M.; Sangoro, J. R.; Kremer, F. Electrode Polarization and Charge Transport at Solid Interfaces. *Phys. Rev. B* **2009**, *80* (18), 184301.
(20) Drakopoulos, S. X.; Karger-Kocsis, J.; Psarras, G. C. The Effect of Micro-fibrillated Cellulose upon the Dielectric Relaxations and DC Conductivity in Thermoplastic Starch Bio-composites. *J Appl Polym Sci* **2020**, *137* (48), 49573.
(21) Drakopoulos, S. X. Revisiting the Dielectric Spectrum: Tricks and Treats of Analysis and Interpretation Around the Conductivity Relaxation. *IET Nanodielectrics* **2025**, *8* (1), e70020.
(22) Drakopoulos, S. X.; Cui, J.; Asandulesa, M.; Blom, P. W. M.; Nogales, A.; Asadi, K. Universal Scaling of DC Conductivity with Dielectric Interfacial Polarization in Conjugated Polymers. *Macromolecules* **2024**, *57* (6), 2661–2668.
(23) Namikawa, H. Characterization of the Diffusion Process in Oxide Glasses Based on the Correlation between Electric Conduction and Dielectric Relaxation. *Journal of Non-Crystalline Solids* **1975**, *18* (2), 173–195.
(24) Dyre, J. C.; Schrøder, T. B. Universality of Ac Conduction in Disordered Solids. *Rev. Mod. Phys.* **2000**, *72* (3), 873–892.
(25) Wang, Y.; Fan, F.; Agapov, A. L.; Saito, T.; Yang, J.; Yu, X.; Hong, K.; Mays, J.; Sokolov, A. P. Examination of the Fundamental Relation between Ionic Transport and Segmental Relaxation in Polymer Electrolytes. *Polymer* **2014**, *55* (16), 4067–4076.
(26) Li, B.; Randall, C. A.; Manias, E. Polarization Mechanism Underlying Strongly Enhanced Dielectric Permittivity in Polymer Composites with Conductive Fillers. *J. Phys. Chem. C* **2022**, *126* (17), 7596–7604.
